# Management of inflammatory cardiopulmonary manifestations in systemic lupus erythematosus: a systematic review

**DOI:** 10.1186/s42358-026-00551-1

**Published:** 2026-05-29

**Authors:** Yanjie Hao, Ricardo Montes, Shereen Oon, Mandana Nikpour

**Affiliations:** 1 The University of Melbourne at St Vincent’s Hospital, 29 Regent Street, Fitzroy, VIC 3065 Australia; 2 Department of Rheumatology, St. Vincent’s Hospital Melbourne, 41 Victoria Parade, Fitzroy, VIC 3065 Australia; 3 Rheumatology Department, Universidade Federal do Rio de Janeiro, Rio de Janeiro, Brazil; 4 University of Sydney Musculoskeletal Research Centre and School of Public Health, Charles Perkins Centre, Johns Hopkins Drive, Camperdown, NSW 2050 Australia; 5 Department of Rheumatology, Royal Prince Alfred Hospital, 57 Missenden Road, Camperdown, NSW 2050 Australia; 6 The University of Sydney School of Public Health, University of Sydney, Room 132, Edward Ford Building, Fisher Road, Sydney, NSW 2006 Australia

**Keywords:** Cardiac, Pulmonary, Immunosuppressive treatments, Biologics, Systemic lupus erythematosus

## Abstract

**Background:**

Cardiopulmonary involvement in systemic lupus erythematosus (SLE) refers to a group of serious manifestations, with a high prevalence for some, such as pericarditis and pleuritis, and high mortality in others, such as lupus myocarditis (LM) and diffuse alveolar haemorrhage (DAH). There is a paucity of systematic literature reviews on the management of inflammatory cardiopulmonary manifestations in SLE. We aimed to assess the available evidence in this area.

**Methods:**

A systematic literature search was conducted from January 1990 to June 2025 using keywords related to the cardiac and pulmonary systems, SLE, and treatments. Manifestations related to atherosclerosis or anti-phospholipid syndrome were excluded.

**Results:**

A total of 67 studies, covering pericarditis, LM, pulmonary arterial hypertension (PAH), pleuritis, acute lupus pneumonitis (ALP), DAH, interstitial lung disease, and shrinking lung syndrome (SLS) were included. Glucocorticoids (GCs) were utilized as first-line treatment for all these conditions, and GC pulse was commonly administered in serious manifestations such as pericardial tamponade, LM, ALP, and DAH. Cyclophosphamide was the most used immunosuppressant, with low-to-moderate evidence indicating its effectiveness. Low-to-moderate evidence supported rituximab as a second-line therapy for LM, DAH, and SLS, and plasma exchange was commonly used in DAH. For PAH, high-quality evidence from RCTs supported the use of PAH-targeted therapies, and initial PAH-targeted combination therapy showed more benefits than monotherapy. In addition, immunosuppressive therapy may be beneficial for SLE-PAH patients, especially those with early, mild PAH. Anti-fibrotic therapy, such as nintedanib and pirfenidone, had no evidence of effectiveness in SLE-ILD. Evidence for the benefit of belimumab and anifrolumab for cardiopulmonary manifestations was also minimal. Patients with some conditions, such as LM, ALP, and DAH, had poor prognoses under current therapies, with reported short-term mortalities of up to 40%-60%.

**Conclusions:**

GC and conventional IS currently remain the most evidence-based approaches for managing most cardiopulmonary manifestations in SLE. Despite aggressive immunosuppressive therapies, patients with some conditions, such as LM, ALP, and DAH, still experience high mortality. The quality of the existing evidence and the insights into novel therapies in these patients were limited, highlighting the urgent need for RCTs and optimized management in this area.

**Supplementary Information:**

The online version contains supplementary material available at 10.1186/s42358-026-00551-1.

## Introduction

Systemic lupus erythematosus (SLE) is an autoimmune disease with multi-organ involvement [1]. It can have high mortality due to its complex and heterogeneous nature, paucity of highly effective therapies and lack of evidence-based target-driven approaches to treatment [1, 2]. Mucocutaneous, musculoskeletal, renal, serosal, and haematological SLE manifestations are the most common and widely investigated [1]. In comparison, cardiac and pulmonary manifestations are less recognized and reported, although some are also relatively common, such as pericarditis and pleuritis [3–5], or can cause high morbidity and mortality, such as myocarditis and diffuse alveolar haemorrhage (DAH) [3, 4]. In the course of the disease, cardiac involvement occurs in > 50% of SLE patients [6], and the prevalence of pulmonary involvement in SLE ranges from 50% to 70% [4, 5, 7]. Those with cardiac or pulmonary manifestations have been shown to have significantly lower survival rates than those without [5, 8].

All components of the heart, including the pericardium, conducting system, myocardium, valves, and coronary arteries, can be involved in SLE patients [3]. Likewise, multiple parts of the respiratory system can be impacted, such as the airways, pleura, pulmonary parenchyma, pulmonary vessels, respiratory muscles, and diaphragm [9]. The pathogenesis of cardiopulmonary involvement in SLE is complex, heterogeneous, and not well defined. In general, autoimmune/inflammatory processes, accelerated atherosclerosis, and thromboembolism are thought to contribute [9, 10]. Endomyocardial biopsy has shown myocarditis, antimalarial cardiotoxicity, small-vessel thrombosis, calciphylaxis, endomyocardial fibrosis, giant cell myocarditis, and hypertrophic cardiomyopathy in SLE patients with myocardial dysfunction [10]. Pleuritis, pleural effusion, interstitial pneumonitis, interstitial fibrosis, vasculitis, alveolar wall necrosis, alveolar haemorrhage, hematoxylin bodies, edema, and hyaline membrane change were seen on pulmonary histology samples of SLE patients [9]. The manifestations of cardiopulmonary involvement in SLE range from more frequent conditions such as pleuritis, pericarditis, and coronary artery disease (CAD), to less common conditions such as myocarditis, endocarditis, pulmonary hypertension (PH) or pulmonary arterial hypertension (PAH), acute lupus pneumonitis (ALP), DAH, interstitial lung disease (ILD), and shrinking lung syndrome (SLS) [3, 7, 9, 11]. This systematic review mainly focused on treatment for autoimmune/inflammatory-mediated manifestations, including pericarditis, myocarditis, PAH, pleuritis, ALP, DAH, ILD, and SLS. Conditions mediated predominantly by atherosclerosis or thromboembolism, which are managed mainly with antiplatelet or anticoagulant agents, such as CAD, antiphospholipid antibody-related endocarditis, and pulmonary thromboembolism, were not included in the current review.

Challenges remain in relation to the management of cardiopulmonary manifestations to reduce morbidity and mortality [5, 8]. Firstly, there is a lack of high-quality evidence regarding treatments, such as randomized clinical trials (RCTs), for most of the cardiopulmonary manifestations of SLE. The existing RCTs in SLE recruited very few patients with these manifestations. For example, the BLISS-52 and 76 trials recruited a small number of patients with cardiovascular/respiratory involvement but did not report the specific presentations and treatment outcomes of these patients [12–14]. Moreover, to our knowledge, there is no comprehensive systematic literature review or standardized guideline regarding the treatment of cardiopulmonary manifestations in SLE. The aim of this study was to systematically and comprehensively review the literature on immunosuppressive, targeted, and adjunctive treatment for different inflammatory cardiopulmonary manifestations in SLE and provide evidence for the formulation of treatment guidelines in this area.

## Methods

### Literature search

The electronic databases OVID MEDLINE and EMBASE were searched from January 1990 to June 2025 to identify studies of cardiopulmonary manifestations in SLE. An additional search for clinical trials was conducted in EMBASE. The keywords for the search included ‘systemic lupus erythematosus’, ‘SLE’, ‘lupus’, and ‘cardiopulmonary’. The full search terms and search strategy are available in Supplementary File 1.

### Study selection

The search protocol was established by three authors (RW, SO, and MN). Subsequently, two authors, YH and SO, conducted independent screening of abstracts to determine potential relevance. Articles identified as potentially relevant were then subjected to full text, applying the following specific inclusion and exclusion criteria to guide the evaluation process. The third author (MN) was consulted if agreement was not reached. Author YH performed data extraction and conducted hand-searching for additional relevant studies.

### Inclusion/exclusion criteria

Clinical trials, observational studies, case series, and case reports with five or more patients investigating management for inflammatory cardiopulmonary manifestations of SLE and cardiopulmonary-specific treatment responses or outcomes were included in this study. Inflammatory cardiopulmonary manifestations were included if their etiology was based primarily on autoimmune-mediated inflammation, and immunosuppressive therapy was necessary. Many of the studies or trials on PAH or ILD broadly included patients with various CTDs, such as scleroderma (SSc), idiopathic inflammatory myopathies (IIM), mixed connective tissue disease (MCTD), Sjogren’s syndrome (SS) or rheumatoid arthritis (RA); they were included in this study provided SLE patients were studied. Cardiopulmonary manifestations secondary to atherosclerosis or anti-phospholipid antibodies/ anti-phospholipid syndrome and solely requiring antiplatelet or anti-coagulant therapy were excluded from this study. We also excluded studies reporting pediatric populations, not available in English, or not available in full text, and systematic reviews.

### Population

Adults with a confirmed SLE diagnosis meeting any recognized SLE classification criteria, i.e. 1982 American College of Rheumatology (ACR) [15], 1997 ACR [16], 2012 Systemic Lupus International Collaborating Clinics classification (SLICC) [17], and the 2019 European League Against Rheumatism (EULAR)/ACR criteria [18], who were treated for cardiac and/or pulmonary manifestations of SLE.

### Intervention

This study included pharmacological and non-pharmacological interventions but mainly focused on pharmacological therapy for inflammatory cardiopulmonary manifestations of SLE, including immunosuppressive medications (including biologics), specific therapy for PH and anti-heart failure medications. Non-pharmacological interventions included pericardiocentesis, pericardial window, extracorporeal membrane oxygenation (ECMO) and intra-aortic balloon pump counter pulsation (IABP).

### Comparator/control

No comparison or control group was required for inclusion in this review.

### Outcomes

#### Primary outcomes

In terms of response to treatment, there was no consensus on outcome measures for most cardiopulmonary manifestations of SLE. Most studies used clinician-reported outcomes to assess clinical response. For myocarditis-related studies, some studies used the New York Heart Association (NYHA) functional class (FC) or left ventricular ejection fraction (LVEF) as an objective parameter to assess treatment response. For PAH-related studies, some widely validated and accepted measures were used to evaluate treatment response, including NYHA or World Health Organization (WHO) FC, 6-minute walk distance (6MWD), brain natriuretic peptide (BNP) or N-terminal proBNP (NT-proBNP) serum levels, and some hemodynamic parameters such as pulmonary arterial pressure (PAP), cardiac output (CO), and cardiac index (CI). For other pulmonary manifestations, such as SLS or ILD, pulmonary function test (PFT) parameters, including forced vital capacity (FVC) and diffusing capacity of the lung for carbon monoxide (DLCO), were used to evaluate treatment response. Some studies used specific definitions for responders.

Overall response (%) is shown as the proportion of patients who achieved a response (partial or complete responses) to overall treatment according to the definition and outcome measure used in that study.

#### Secondary response measures

(1) Mortality or survival if reported; (2) Adverse events (AE) and serious adverse events (SAE) if reported; (3) Recurrence of manifestation within the reported follow-up duration.

### Statistical analysis

Due to the high heterogeneity between studies, we did not conduct a meta-analysis of outcomes in this review.

### Data collection and data items

Data collection was conducted using the Covidence systematic review management platform. Information extracted from the articles included study aim and design, demographic characterizations of the population, interventions and outcomes.

### Risk of bias in individual studies

A risk-of-bias assessment was performed for all studies included in the review. For all RCTs or non-randomized studies with a comparator group, we utilized the Covidence Risk of Bias template. This template evaluates the risk of bias across eight domains, categorizing it as “high,” “low,” or “unsure” (see Supplementary Table 1). Studies without comparison groups were classified as having a high overall risk of bias. YH conducted the risk-of-bias assessments, while SO resolved any uncertainties.

## Results

### Study selection

**Fig. 1:**
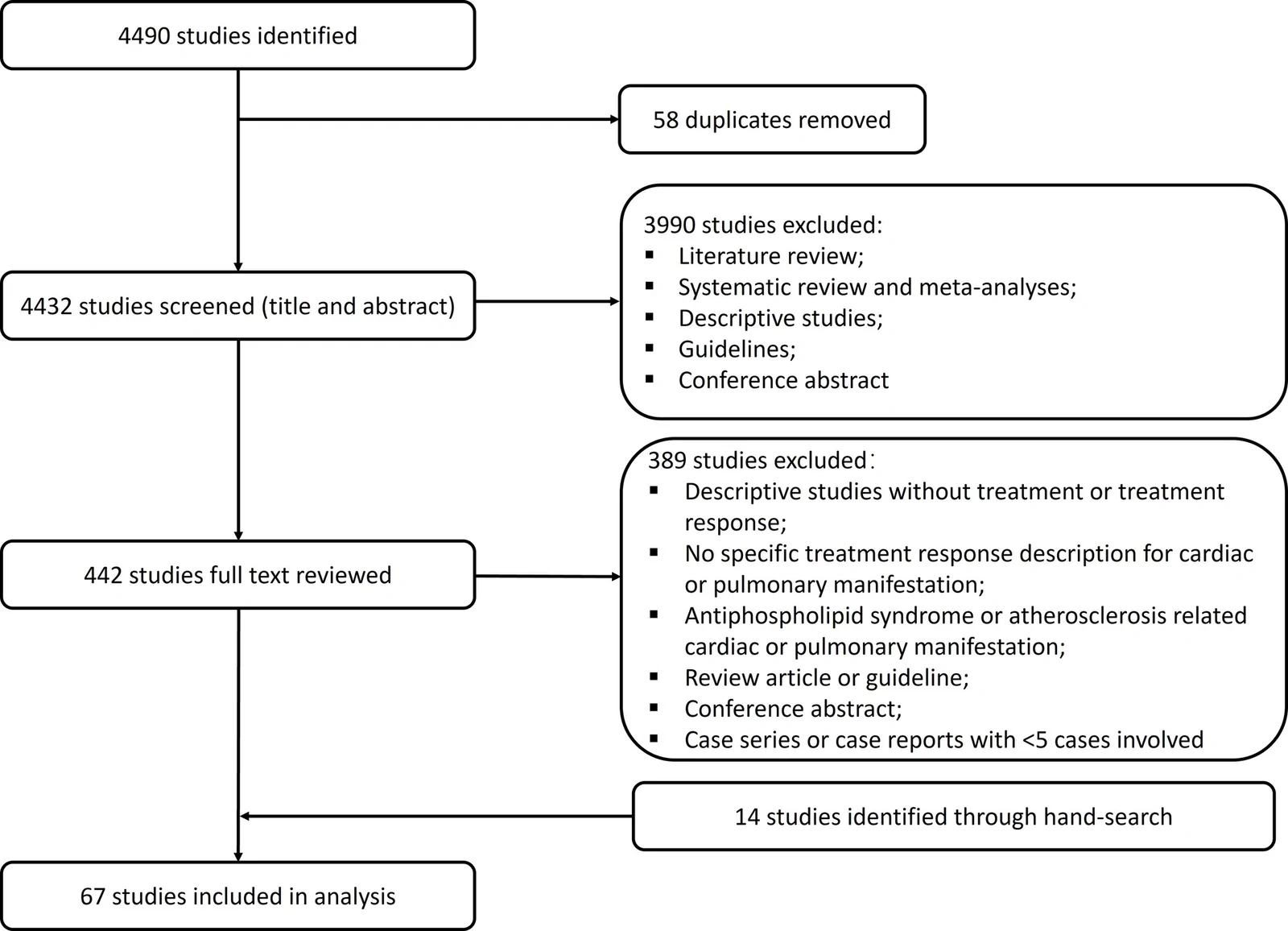
Flow diagram of study selection

The initial search identified 4,490 studies, from which 58 duplicates were removed. In the following abstract screening stage, 3,990 studies were excluded for the reasons outlined in Fig. 1. Subsequently, 442 studies were retrieved for full-text review, and an additional 388 studies were excluded. A hand search of relevant journals yielded another 14 articles. In total, 67 studies were ultimately included in this review.

### Study categories

The 67 studies included 2118 SLE patients, and they were categorized according to disease manifestations. Eight specific types of manifestations were included in this review, including pericarditis (9 studies), myocarditis (9 studies), PH/PAH (23 studies), pleuritis (1 study), pneumonitis (1 study), DAH (12 studies), ILD (6 studies), and SLS (5 studies). In addition, one RCT which reported treatment response in cardiopulmonary manifestations was also included (1 study).

### Study results

#### Pericarditis

There were nine studies with 246 patients reporting treatment for pericarditis in SLE, including six observational studies and three case series [19–27]. The summarized results are shown in Table 1, and the details of each study are listed in Supplementary Table 2. Among the 246 patients, 59 patients were reported as having tamponade. Treatment included glucocorticoids (GCs) (8 studies), conventional immunosuppressants (IS) (4 studies), biologics including rilonacept (1 study) and rituximab (RTX) (2 study), non-steroid anti-inflammatory drugs (NSAIDs) (3 studies), intravenous immunoglobulin (IVIG) (1 study), colchicine (2 studies), and interventional procedures including pericardiocentesis and pericardial window (3 studies). There was no comparator for treatment in the nine studies.

**Table 1:** Summary of included studies in pericarditis and myocarditis

Number and type of studies	Number of total patients	Treatments				Outcomes	
		GC	Conventional IS	Biologics	Other treatment	Primary outcomes	Secondary outcomes
***Pericarditis***							
6 observational studies 3 case series [19–27]	246	**Number**: reported in 8 studies, in 75%-100% of patients **Regimen**: 5 studies reported *GC alone* in 16.7%-100% of patients. **Dose**: ranged from low to high. *Pulsed methyl-PNL* was mainly for large pericardial effusion or cardiac tamponade.	**Type and Number**: − *CYC (oral or IV)*: 2 studies, in 5.4% and 83.3% of patients, respectively. All used in patients with cardiac tamponade.	**Type and Number**: − *Rilonacept* :reported in 1 case study, in 4 patients with refractory recurrent pericarditis and pleuritis; and 1 study reported − *RTX*: reported in 1 study, in 1 refractory patient	**Type and Number**: − *NSAIDs*: reported in 3 studies, in 2.6%, 35%, and 100% of patients, respectively. − *Colchicine*: 2 studies, in 100% and 75% ofpatients, respectively. − *IVIG (small dose)*: reported in 1 study, in 62 patients. − *Pericardiocentesis and pericardial window*: reported in 3 studies, in 90%-100% of patients with cardiac tamponade.	**Pericarditis resolution**: reported in 9 studies, in 60%-100% of patients	**Mortality**: reported in 3 studies, in 8.3%-25% of patients; **Recurrence**: reported in 5 studies, in 20%-40% of patients
**Myocarditis**							
5 observational studies 4 case series [28–36]	225	**Number**: reported in 9 studies, in 88%-100% patients **Regimen**: GC + IS± IVIG commonly used in 60%-100% of patients; GC alone reported in 6 studies, in 17.9%%-80% of patients. **Dose**: moderate to high dose in all studies, with pulsed methyl-PNL in 36.3%-100% of patients.	**Type and Number**: − *CYC (IV)*: reported in 8 studies, in 10%-83.3% of patients. − *MMF*: reported in 5 studies, in 6.9%-25% of patients. − *AZA*: reported in 4 studies, in 12.5%-40% patients − *Other IS*: CsA and Tac reported in in 1 study for 2.5% and 10.1% of patients, respectively	**Type and Number**: − *RTX*: reported in 3 studies, in 4.2%-23.1% patients	**Type and Number**: − *IVIG*: reported in 6 studies, in 4.2%-35.4% of patients − *PE*: reported in 3 studies, in 7.1%-13.8% of patients − *Anti-heart failure medications*: reported in 7 studies, in 20%-75% of patients − *Supportive therapy* such *as ventilation*,* ECMO*,* IABP*,* CVVH*: reported in 5 studies, in 20%-67.9% of patients	**LVEF improvement**: reported in 8 studies, in 50%-100% of patients	**Mortality**: reported in 8 studies, in 0%-42.9% of patients **Recurrence**: 2 studies, in 7.1%-7.7% of patients

Abbreviation: AZA: azathioprine; CsA: cyclosporin; CYC: cyclophosphamide; CVVH: continuous veno venous haemofiltration; ECMO: extracorporeal membrane oxygenation; IABP: intra-aortic balloon pump counter pulsation; IV: intravenous; IS: immunosuppressant; IVIG: intravenous immunoglobulin; LVEF: left ventricular ejection fraction; methyl-PNL: methylprednisolone; MMF: mycophenolate; NSAIDs: non-steroids anti-inflammatory drugs; PE: plasma exchange; RTX: rituximab; Tac: Tacrolimus

##### Glucocorticoids

As the most commonly used medication in these studies, GC usage was reported by eight studies for 97.8% of the patients [19, 20, 22–27]. In terms of specific regimen, the use of GC alone was reported by 5 studies in 16.7%-100% of patients [19, 20, 23–25], and GC in combination with other medications such as hydroxychloroquine (HCQ), IS, IVIG, or colchicine was reported by 7 studies [20–22, 24–27]. GC dose varied from low (range 2–10 mg/d) (1 study) [26], moderate (mean prednisolone (PNL) dose 33.6 ± 15 mg/d) (1 study) [20], to high (≥ 60 mg/d or ≥1 mg/kg/d) (4 studies) [19, 22, 24, 25] including three studies reporting pulsed methylprednisolone (methyl-PNL) mainly for patients with large pericardial effusion or cardiac tamponade [22, 24, 25]. GC-specific treatment response for pericarditis could be identified from three studies, and the overall response rate to GC was 60%--80% (6/10, 61/76, 6/7, respectively) [19, 23, 25], even for patients with cardiac tamponade.

##### Hydroxychloroquine

Two studies reported HCQ use in SLE patients with pericarditis. One observational study reported on 10 lupus-related pericarditis cases [22]. All ten patients received high-dose GC combined with HCQ (400 mg/d), including eight (80%) with oral prednisone (60 mg/d) and two with pulse methyl-PNL (500 mg/d for three days) followed by oral prednisone. All ten patients (100%, 10/10) achieved a complete response to treatment.

##### Conventional immunosuppressants

IS usage was not reported as commonly as GC for pericarditis in the included studies. Only two observational studies reported oral or intravenous (IV) cyclophosphamide (CYC) use on 5.4% (2/18) and 83.3% (20/24) of the patients, respectively; and all of these patients presented with tamponade [20, 24]. CYC was combined with GC in all cases, and the overall response to this combination therapy was 100% (35/35) and 95% (23/24), respectively, in these two studies [20, 24].

##### Biologics

A case series reported four pericarditis and pleurisy refractory to previous treatments, including NSAIDs, colchicine, HCQ, GC, and/or IS, that finally attained good response to rilonacept, an interleukin-1α (IL-1α) and IL-1β inhibitor that has been approved for idiopathic recurrent pericarditis. For two of the patients, treatment was initially switched to another IL-1 inhibitor, anakinra (ANK), before they received rilonacept. While ANK showed a favourable response, it could not be maintained due to significant injection site reactions for one patient and secondary failure for the other patients. Overall, all four patients achieved a complete response or significant improvement with rilonacept [27]. One study reported one patient (4.2%, 1/24) who had no response to GC pulse plus two doses of IV CYC was further treated with RTX. However, the response to RTX was not reported [24].

##### Other medications, including non-steroid anti-inflammatory drugs, intravenous immunoglobulin, and colchicine

In addition to GC and IS, four studies reported the efficacy of other medications [20, 21, 23, 26], including NSAIDs in three studies [20, 23, 27] and one study using NSAIDs alone for two patients with mild serositis (100% response, 2/2) [20], low-dose IVIG as an add-on therapy (60% response, 38/60) [21], and colchicine in two studies as an add-on therapy [26, 27], with one of them showing a 100% response to colchicine (10/10). There was no recurrence during colchicine treatment, but 20% of the patients (2/10) recurred after discontinuation of colchicine [26].

##### Interventional procedures

Interventional procedures, including pericardiocentesis and pericardial window, were conducted before or along with medications for cardiac tamponade in 41 patients, as reported by three studies [19, 24, 25]. These interventional procedures were usually reserved for the emergency management of tamponade and could resolve hemodynamically unstable situations caused by tamponade [19, 24, 25]. Compared with pericardial window surgery (5 patients), pericardiocentesis was more commonly conducted (37 patients).

In summary, the evidence for pericarditis indicates that moderate to high-dose oral GC alone was the most common regimen. GC pulse or a combination of GC with IS, such as CYC, was prescribed mainly for large pericardial effusion or tamponade. IL-1 inhibitor rilonacept showed effectiveness in case reports as a second-line therapy when high-dose GC and IS were ineffective. NSAIDs alone were solely used for mild pericarditis, and colchicine was effective for pericarditis in addition to immunosuppressive drugs, with good tolerance and apparent steroid-sparing effect. The overall response rate of lupus pericarditis was high; 80%--100% in overall patients and 60%-95.8% in patients with tamponade. However, recurrences were common in around 20%-40% of patients, but these cases responded to increased GC dose (100% response according to two studies) [20, 22].

#### Myocarditis

Nine studies reported lupus myocarditis (LM) in 225 patients, including five retrospective observational studies and four case series/reports (Table 1 and Supplementary Table 3) [28–36]. There were two categories of treatment for LM in these studies, including (i) immunosuppressive drugs such as GC (9 studies), IS (9 studies), IVIG (6 studies), RTX (4 studies), and immune-related therapy such as plasma exchange (PE) (3 studies), and (ii) anti-heart failure medications (7 studies) such as angiotensin-converting enzyme inhibitors (ACEI) or angiotensin receptor blockers (ARB), beta-blocker, diuretics, digoxin, inotropic drugs, and anti-heart failure interventions or auxiliary treatment such as ECMO (1 study), IABP (1 study), continuous veno-venous hemofiltration (CVVH) (2 studies) and ventilation (3 studies). No study had a treatment comparator.

##### Glucocorticoids

GC use was reported in all nine studies, with 88%-100% of patients receiving it, and all studies reported a proportion of patients (36.3%-100%) treated with pulse IV methyl-PNL. Two case series reported repeated pulse methyl-PNL courses due to resistant or relapsed LM [29, 34]. The most common regimen of methyl-PNL pulse therapy in these studies was 500-1000 mg/d for 1–3 days, followed by high-dose oral GC [28–30, 32–34, 36], and the average dose of oral GC was above 0.5 mg/kg/d (0.5-1 mg/kg/d in 3 studies, 1 mg/kg/d in 2 studies, 60 mg/d in 1 study, and 40–80 mg/d in 1 study). GC alone was reported by 6 studies in 17.9%%-80% of patients [28–33], and the combination of GC and IS and/or IVIG was used more commonly (60%-100%) in most included studies [28–32, 34–36], except one case series, where 80% (4/5) patients with LM were treated with high-dose GC alone [33]. The overall response to GC and IS combination therapy ranged from 43% to 89%, but the separate treatment outcomes from GC alone can only be identified in the case series study mentioned above, where 75% of the patients (3/4) with LM responded to high dose GC alone. However, one patient on GC alone died of relapsed LM six months after initial treatment [33].

##### Conventional immunosuppressants

IS use was reported in all nine studies [28–36]. CYC was the most commonly used IS, prescribed to 10%-83.3% of patients as the first-line IS for remission induction, and to two patients as the second-line therapy when first-line AZA was ineffective. Intravenous CYC pulse therapy dose varied from 300 to 750mg/m^2^ monthly for 3 to 12 months. AZA use was reported in 12.5%-40% patients in four studies as induction therapy or maintenance therapy. Five studies reported MMF use in 6.9%-25% patients, with most of them for induction therapy and one for maintenance therapy after CYC. Other less common IS agents reported included Tacrolimus (Tac) and Cyclosporin (CsA). The overall response to the combination of GC and IS was 43%-89%.

##### Rituximab

RTX use was reported in three studies. An observational study examined the efficacy of RTX for patients with LM from a single-center cohort [31]. Three out of 13 patients with LM in this cohort received RTX (375 mg/m^2^ fortnightly × 2 or weekly ×4) as an add-on therapy to high-dose GC and IS (AZA, CYC, or MMF) for refractory LM. All three patients (100%, 3/3) showed positive results, with NYHA FC improving from III or IV to I, and LVEF increasing in two patients [31].

##### Intravenous immunoglobulin

Six studies reported IVIG use for LM in 4.2%-35.4%, and most of them were refractory or relapsed cases. IVIG appeared to be effective as a combination therapy with GC ± ISs [28, 29, 33–36]. A case series reported one patient who had no improvement after GC pulse was treated with IVIG (0.4 g/kg/day for five days) and IV CYC, with a slow improvement after one month of this treatment [33]. Another case series reported a refractory patient who had no response to the initial course of GC pulse and CYC pulse. The subsequent treatment with repeated GC pulse, CYC, and three courses of IVIG (36 g/day for 4 days/course for three courses) resulted in a gradual improvement in cardiac function [34].

##### Plasma exchange

PE was reported in three studies involving 7.1%-13.8% of patients, with most treated with PE in combination with immunosuppressive therapy and one with PE alone [28, 29, 36]. The patient treated with PE alone died before initiating GC [28], and the outcomes of the other 12 patients treated with the combination of PE and other immunosuppressive therapy were not separately reported.

##### Anti-heart failure medications and other supportive therapy

Seven studies reported anti-heart failure medication use in 20%-75% of patients, including ACEI/ARB, beta-blockers, digoxin, diuretics, inotropic and vasopressors, in addition to immunosuppressive therapy [28–30, 32–35]. Other supportive therapies for critical patients, such as ECMO, IABP, CVVH, and ventilation, were reported in some studies [28, 29, 33, 34]. No specific treatment response to these supportive therapies was described, but these cases show that these measures can provide critical support during severe cardiovascular dysfunction.

In summary, the most commonly prescribed treatment regimen for LM was high-dose GC, including pulse methyl-PNL, often combined with IS or IVIG, and was effective. CYC was the most commonly used IS. RTX, IVIG, and PE had limited evidence, mainly as second-line therapy for refractory LM. The overall treatment response rate to the intensive combination therapy varied from 43% to 100%. The mortality rate was 11.5%-42.9% under immunosuppressive therapy, and 8.3%-20% of patients died of LM itself.

#### Pulmonary hypertension/pulmonary arterial hypertension

We included 23 PH/PAH-related studies in five study types: (i) two RCTs [37, 38] and six post-hoc or subgroup analyses of RCTs [39–44]; (ii) one non-randomized trial [45]; (iii) one prospective non-controlled cohort study [46]; (iv) twelve observational studies [47–58]; and (v) one case series [59] (Table 2 and Supplementary Table 4). Some studies investigated PH/PAH in various CTD types rather than SLE alone and often did not report SLE-specific outcomes [37, 39–44, 46, 48, 50, 52, 54, 56]. In total, 2364 patients with various CTDs, including 964 patients with SLE, were included in the 23 studies, wherein five studies made the PH diagnosis by echocardiography [38, 45–47, 53], and 18 studies confirmed the diagnosis according to right heart catheterization (RHC). Here, PAH diagnosis would be made if the hemodynamic characteristics fulfilled the criteria for precapillary PH [37, 39–44, 48–52, 54–59].

**Table 2:** Summary of included studies in pulmonary hypertension/pulmonary arterial hypertension

Number and type of studies	Number of total patients	Treatments			Outcomes	
		Comparator	Immunosuppressive therapy	PAH-targeted therapy	Primary outcome	Secondary outcome
**Studies investigating efficacy of immunosuppressive therapy**						
1 RCT in SLE-PAH [38]	34 SLE-PAH	Yes − IV CYC vs. oral enalapril	**Type and Number**: − *CYC vs. Enalapril*	**No**	**Reduction of eSPAP from baseline to 6** **months**: 80% vs. 66.7%, Subgroup (patients with eSPAP ≥ 35 at baseline): 100% vs. 30%	**FC improved from baseline to 6 months**: yes in CYC group vs. no in Enalapril group
6 observational studies, including 3 in SLE-PAH and 3 in CTD-PAH [47, 48, 51, 54, 55, 58]	429 CTD-PAH*, including 384 SLE-PAH	No	**Type and Number**: − *GC*: reported in 6 studies, in 78.6%-100% of patients, including pulsed methyl-PNL reported by 3 studies in 17.9%-44.4% of patients − *CYC*:reported in 6 studies, in 25%-100% of patients − *AZA*: reported in 2 studies, in 3.3%-21.4% of patients − *MMF*: reported in 2 studies, in 3.3%-14.3% of patients − *Tac*: reported in 1 study, in 3.3% of patients **Regimen**: − GC alone: reported in 2 studies, in 41.7%-66.7% of patients − GC + IS: reported in 6 studies, in 33.3%-100% of patients	Reported in 5 studies, in 66.7%-100% of patients	**Treatment responder in FC or hemodynamic measures**,** depending on specific studies**: reported in 6 studies, in 23.1%-87.5% of patients	**Mortality**: reported in 5 studies, in 8.3%-53% of patients
**Studies investigating efficacy of PAH-targeted therapy**						
1 RCTs 6 post-hoc studies or subgroup analyses of RCTs All 7 studies in CTD-PAH [37, 39–44]	914 CTD-PAH*, including 185 SLE-PAH	Yes − PAH-targeted medication vs. placebo; − PAH-targeted combination therapy vs. monotherapy	Reported in 4 studies, in 14%-70% of patients	**Type and Number**: − *Sildenafil* vs. placebo: reported in 1 study − *Riociguat* vs. placebo: reported in 1 study − *Bosentan* vs. placebo: reported in 1 study − *Sitaxsentan* vs. placebo: reported in 1 study − *Treprostinil* vs. placebo: reported in 1 study*Selexipag* vs. placebo: reported in 1 study − *Tadalafil+ Ambrisentan* vs. *Tadalafil* alone vs. *Ambrisentan* alone: reported in 1 study	**Change in FC**,** 6MWD**,** eSPAP**,** hemodynamic measures**,** or NT-proBNP at 12**,** 24**,** or 26 wee**ks: 6 studies in monotherapy: significantly higher improvement in PAH-targeted therapy group than placebo group. − 1 study in combination therapy: significantly more benefits from combination therapy than monotherapy	− **Change in HRQoL score at 12**,** 24**,** or 26 weeks**: reported in 3 studies, including 1 study showing significant benefits from PAH-targeted therapy and the other 2 studies showing no significant difference − **Mortality at 12**,** 24**,** or 26 weeks**: 5 studies, including 2 studies showing significantly lower mortality in PAH-targeted therapy group
1 non-randomised trial (strategy trial) in SLE-PAH [45]	22 SLE-PAH	Yes − proactive intense care vs. historical control^#^	mean PNL dose in proactive intense care vs. historical control groups: 44.4 ± 5.8 vs. 166.1 ± 1 20.8 (*p* = 0.354)	**Type and Number**: (proactive intense care vs. historical control) − *Sildenafil*: 4 (36.4%) vs. 1 (9.1%); − *Iloprost*: 8 (72.7%) vs. 0 (0.0%); − *Bosentan*: 8 (72.7%) vs. 1 (9.1%) − *Combination therapy*: 8 (72.7%) vs. 0 (0%). Regimen of combination therapy: Bosentan+Iloprost; Bosentan+Sildenafil, Bosentan+Iloprost+Sildenafil	**FC change at 3 months**: favored proactive intense care group	**Mortality at 3 months** (proactive intense care vs. historical control): 72.7% vs. 27.3%, *p* = 0.033
1 Prospective non-controlled study, 3 Observational studies, and 1 observational study [46, 49, 52, 56, 59]	670 CTD-PAH*, including 66 SLE-PAH	No	Reported in 2 studies, in 58.3%-83.3% of patients-	**Type and Number**: − *Bosentan/other ERA monotherapy*: reported in 3 studies, in 18%-100% of patients − *Epoprostenol*: reported in 3 studies, in 16.7%-100% of patients − *Sildenafil/other PDE5i*: reported in 2 studies, in 8.3%-44% of patients − *Combination therapy*: reported in 2 studies, in 27%-41.7% of patients Regimen of combination therapy: Bosentan+Epoprostenol; Bosentan+Treprotinil Bosentan/other ERA+ Sildenafil/other PDE5i	**FC improvement**: reported in 4 studies, in 66.7%-100% of patients **6MWD improvement**: reported in 2 studies, in 54%-85.7% of patients **mPAP level decrease**: reported in 4 studies, in 54.5%-100% of patients **CI/CO improvement**: reported in 3 studies, in 80%-100% of patients	**Mortality**: reported in 4 studies, in 0%-68.8% of patients
**Studies investigating efficacy of combination of immunosuppressive and PAH-targeted therapy**						
3 observational studies, including 2 in SLE-PAH and 1 in CTD-PAH [50, 53, 57]	283 CTD-PAH*, including 273 SLE-PAH	Yes in 1 study: immunosuppressive therapy alone vs. immunosuppressive therapy combined with pulmonary vasodilators	**Type and Number**: − *GC*: reported in 3 studies, in 95.3%-100% of patients, − *CYC/other IS*: reported in 3 studies, in 83.9%-100% of patients	**Type and Number**: − *Bosentan/other ERA*: reported in 2 studies, in 13.0%-50% of patients − *Epoprostenol/Treprostinil*: reported in 2 studies, in 2.1%-17.4% of patients − *Sildenafil/Tadalafil/other PDE5i*: reported in 2 studies, in 48.7%-83.3% of patients	**Treatment responder in FC or hemodynamic measures**,** depending on specific studies**: reported in 2 studies, in 45.8%-57.1% of patients Treatment responder in immunosuppressive therapy alone vs. immunosuppression and pulmonary vasodilators combination therapy: 50% vs. 57.1% [49]	**Mortality**: reported in 1 study, in 8.3% of patients

* Some studies investigated extensive CTD-PAH patients rather than solely SLE-PAH patients

# The core of the proactive intense care strategy was the appropriate initiation of PAH-targeted combination therapy, using at least two of the three drugs, iloprost, bosentan, and sildenafil. Key elements also included training courses on SLE-PAH for ED physicians; and implementing a SLE-PAH patient triage protocol with prompt specialist consultation and admission. A historical group of severe SLE-PAH patients without receiving this proactive care strategy was selected as control group

Abbreviation: AZA: azathioprine; CTD: connective tissue disease; CYC: cyclophosphamide; eSPAP: estimated systolic pulmonary arterial pressure; FC: functional class; IS: immunosuppressant; IV: intravenous; MMF: mycophenolate; mPAP: mean pulmonary arterial pressure; 6MWD: 6-minute walk distance; NT-proBNP: N-terminal pro brain natriuretic peptide; PAH: pulmonary arterial hypertension; PAP: pulmonary arterial pressure; SLE: systemic lupus erythematosus; Tac: tacrolimus

Among the 23 included studies, 13 studies (one RCT, six post-doc studies of RCTs, one non-randomized trial, one prospective non-controlled study, three observational studies, and one case series) focused on PAH-targeted therapy [37, 39–46, 49, 52, 56, 59], seven studies (one RCT and six observational studies) aimed to investigate the efficacy of immunosuppressive therapy [38, 47, 48, 51, 54, 55, 58], and three observational studies tried to evaluate the combination of PAH-targeted and immunosuppressive therapies [50, 53, 57].

The treatment response and patients’ outcomes in these included studies are shown in Table 2 and Supplementary Table 5. The commonly used measurements included NYHA or WHO FC, 6MWD, Borg dyspnea score, serum BNP or NT-proBNP levels, echocardiogram estimated systolic pulmonary arterial pressure (eSPAP), some hemodynamic parameters on RHC such as mean PAP (mPAP), pulmonary vascular resistance (PVR), CO, and CI, mortality, and some self-defined response definitions.

##### Immunosuppressive therapy

Seven studies, including one RCT [38] and six observational studies [47, 48, 51, 54, 55, 58], mainly aimed to evaluate the efficacy of immunosuppressive therapy for CTD-PAH or SLE-PAH patients (463 patients in total, including 384 SLE patients). The seven studies reported GC use in 78.6%-100% of the patients, including methyl-PNL pulse in three studies, oral moderate to high dose (40-60 mg/d) GC in two studies, and one CYC trial only allowing small dose GC (≤ 15 mg/d). IV CYC was the most common IS and was reported by all seven studies for 60% of the patients; the commonly used regimen was 500–600 mg/m^2^ monthly for 3–6 months.

The positive response rate to immunosuppressive therapy in CTD-PAH or SLE-PAH patients was reported as 23.1%-87.5% [38, 47, 48, 51, 54, 55]. Some patients were treatment-naive [48], and some patients were those who did not respond to conventional PAH therapy, such as calcium channel blockers, oral prostaglandin I2, anticoagulants, and diuretics [47]. One RCT compared IV CYC with oral enalapril (an angiotensin converting enzyme inhibitor), and found that CYC had significantly better efficacy in reducing systolic pulmonary artery pressure (SPAP) on transthoracic echocardiography, and improving NYHA FC, than enalapril [38]. In an observational study, intensive immunosuppressive therapy (IV CYC for one year and oral GCs 1 mg/kg/day) plus pulmonary vasodilator therapy significantly improved the haemodynamic parameters and long-term prognosis, compared to vasodilator therapy alone [51].

One observational study in CTD-PAH showed that patients with SLE and MCTD had treatment responses (38% in SLE patients (5/13) and 37.5% in MCTD patients (3/8)) to first-line immunosuppressive therapy (monthly IV CYC ± GC), but SSc patients had no response. Patients with SLE and MCTD who had better baseline NYHA function and pulmonary hemodynamics were more likely to derive significant benefits from immunosuppressive therapy [48]. Another observational study evaluated immunosuppressive therapy as first-line treatment (oral PNL 60 mg/day with or without IS) in CTD-PAH patients, including SLE, MCTD and pSS. A positive response was observed in 53% of the patients (16/30), and PAH-related mortality, with a mean follow-up of 5.5 ± 4.3 years, was 46.7% (14/30). The survival rates were best in patients with a simultaneous PAH and CTD diagnosis treated initially with a combination of GCs and IS [54]. Another observational study aimed to evaluate the reversibility of PAH in SLE patients after immunosuppressive treatment and the predictors of treatment response. It showed that 35.7% of these patients (10/28) achieved complete ‘reversion’ of PAH after a minimum of 6 months of immunosuppressive induction therapy. Compared to the ‘non-reversion’ group, the ‘reversion’ group had a significantly lower baseline SPAP, right ventricle dilatation and hypokinesia frequencies on echocardiogram, and also lower baseline BNP levels and risk stratification score [58].

Three studies aimed to evaluate the effectiveness of combining immunosuppressive and PAH-targeted therapy [50, 53, 57]. One study demonstrated that combination immunosuppressive and PAH-targeted therapy provided greater benefits than immunosuppressive therapy alone in improving function and decreasing PAP [50, 53, 57], and one study showed that immunosuppression may add to the effect of PAH-targeted therapy in SLE-PAH [53]. In addition, all three studies found that patients with mild PAH, such as patients with functional class I/II and/or a CI > 3.1 at baseline, or ‘borderline PAH’ patients with mPAP of 21–24 mmHg and PVR of 2–3 Wood units, were more likely to benefit from immunosuppression therapy alone [50, 53, 57].

##### PAH-targeted therapy

Thirteen studies, including one RCT, six post hoc studies/subgroup analyses of RCTs, one non-randomized trial, one prospective non-controlled study, three observational studies and one case series, focused on PAH-targeted therapies in three different mechanistic pathwaysࣧ endothelin, nitric oxide (NO), and prostacyclin pathways, covering endothelin receptor antagonists (ERA), phosphodiesterase type 5 (PDE-5) inhibitors/soluble guanylate cyclase stimulators (sGCs), and prostacyclin analogues (PCA)/prostacyclin receptor agonist (PRA) [37, 39–46, 49, 52, 56, 59]. Except for the non-randomized trial, one observational study, and one case series [45, 49, 59], all the other ten studies included various types of CTD patients rather than SLE alone, and SSc was usually the predominant type. The specific treatment response in SLE patients was not reported in most of the studies [37, 39–44, 46, 52, 56]. In general, the investigated PAH therapies targeting the three mechanistic pathways, showed efficacy in improving symptoms, physical function, and/or hemodynamic parameters in CTD-PAH patients.

Two medications targeting the endothelin pathway, bosentan and sitaxisentan, known as ERAs, were evaluated in CTD-PAH in two post hoc analyses using data from the original RCTs [39, 41]. Both the analyses from the Study 351 and BREATHE trials for bosentan [39], and the STRIDE trials for sitaxsentan [41]showed that CTD-PAH patients benefited from these two drugs compared to placebo in many aspects, such as 6MWD, FC, and haemodynamics. The analyses from the extension studies of these trials showed that the efficacy could be maintained for a long period of follow-up, and the long-term safety was also confirmed [39, 41]. In addition to these two RCTs, a prospective noncontrolled study evaluated the effects of bosentan on 13 CTD-PAH patients, including two SLE patients, most of whom did not respond to previous prostanoid therapy. After one year of treatment, more than half of the patients had improvements in 6MWD, mPAP, and RVSP. Those who did not show improvement were mostly patients with SSc [46].

The NO pathway included PDE-5 inhibitors and sGCs. Sildenafil is one of the first-generation drugs of PDE-5 inhibitors. One post hoc study analyzed the CTD-PAH subgroup in the SUPER-1 study, a trial of sildenafil [40]. The results showed that the patients who received different doses of sildenafil had significant improvement in 6MWD compared to placebo, and no significant difference in AE was observed between the two groups. Riociguat is a type of sGCs. A subgroup analysis of CTD-PAH patients from the PATENT-1 trial for riociguat showed that riociguat significantly increased 6MWD over 12 weeks of treatment compared with a decrease in the placebo group [44]. The proportion of patients with improvement in WHO FC, hemodynamics, and NT-proBNP was also higher in the riociguat group than in the placebo group. In the extension study, improvements in 6MWD and WHO FC were largely maintained for two years. In the trials, riociguat was well tolerated in CTD-PAH patients. It should be noted that no SLE-PAH specific data are available in both SUPER-1 and PATENT-1 trial.

In the prostacyclin pathway, two studies analyzed the subgroup of patients with CTD-PAH from two original PAH trials. The first trial was for continuous subcutaneous infusion of treprostinil, one of the PCAs, and the second trial was the GRIPHON trial of selexipag, an oral PRA. The Treprostinil trial indicated that treprostinil could improve CTD-PAH patients’ dyspnea symptoms and hemodynamic parameters [37]. Selexipag demonstrated a significant reduction in risk of composite morbidity/mortality and NT-proBNP for overall CTD-PAH patients. For SLE-PAH specific patients, selexipag showed a trend of greater improvement in 6MWD and NT-proBNP and reduced morbidity and mortality, compared to placebo, but it did not reach statistical significance [43]. The safety of these two drugs for CTD-PAH patients was also confirmed. In addition to the two RCTs, one observational study and one case series reported the efficacy of another prostacyclin analogue family member, IV epoprostenol, in CTD-PAH patients [52, 59].

Two clinical trials and one observational study found that combining PAH-targeted therapies targeting different pathways (combination therapy) yields better outcomes than monotherapy [42, 45, 56]. One study examined data from a subgroup of patients with CTD-PAH who participated in the AMBITION trial, where 187 treatment-naive CTD-PAH patients, including 17 SLE patients, were randomly assigned to receive either combination therapy with ambrisentan and tadalafil or monotherapy with ambrisentan or tadalafil [42]. The results showed that initial combination therapy had significantly more clinical benefits than monotherapy in reducing the risk of clinical failure (death, hospitalization, disease progression, or unsatisfactory long-term clinical response), increasing 6MWD, and decreasing NT-proBNP. Peripheral edema, the most common AE, was more frequent in the combination group than in the monotherapy group. A non-randomized clinical trial evaluated the efficacy of a proactive intensive care strategy in treating severe SLE-PAH patients with WHO FC of III or IV, and a historical group of patients with the same inclusion criteria was compared as a control. The core of the proactive intensive care strategy was the appropriate initiation of PAH-targeted combination therapy, using at least two of the three drugs, iloprost, bosentan, and sildenafil. Immunosuppressive therapy could be used as needed. The results demonstrated that compared with the historical group, where only 18.2% of the patients received PAH-targeted therapy, and none of them received PAH-targeted combination therapy, the proactive intensive care, where 72.7% of the patients were initiated with PAH-targeted combination therapy, significantly improved 3-month survival (proactive group vs. historical group, 72.7% vs. 27.3%, *P* = 0.033). In the pooling data, patients who received combination therapy showed significantly better survival than those receiving monotherapy (*P* = 0.0099) [45].

In summary, the treatment for SLE-PAH comprises two major components: immunosuppressive therapy and PAH-targeted therapy. Additionally, conventional supportive therapy for PAH, such as supplemental oxygen, anticoagulants, digoxin, and diuretics, may be utilized if necessary. Besides GCs, CYC had the most evidence as an immunosuppressive therapy. SLE-PAH patients could benefit or potentially achieve complete reversion from immunosuppressive therapy alone, especially in borderline PAH, early-stage PAH, simultaneous PAH and SLE diagnosis, and those with mild disease, as assessed by WHO FC, 6MWD, BNP/NT-proBNP, and pulmonary hemodynamics. However, in general, a combination of immunosuppressive and PAH-targeted therapies benefit SLE-PAH patients more than immunosuppressive therapy alone. For PAH-targeted medications, most evidence was from trials or studies in general CTD-PAH patients, and all three major pathogenetic pathway-related medications showed efficacy in improving function, pulmonary hemodynamics, and long-term prognosis. Although most evidence is for monotherapy, initial combination therapy showed better outcomes than monotherapy.

#### Pleuritis

Only one observational study about lupus pleuritis was included in this review (Table 3 and Supplementary Table 6) [60] This single-center retrospective study included 119 SLE patients with 127 episodes of pleural effusion. Of these, 93% were treated with GCs (the dose was not described) and 3% with NSAIDs. At the end of follow-up, 94% of patients (112/119) had a complete response to the initial treatment. However, recurrence happened in 14% (12/119).

**Table 3:** Summary of included studies in pleuritis, acute pneumonitis, diffuse alveolar haemorrhage, interstitial lung disease, and shrinking lung syndrome

Number and type of studies	Number of total patients	Treatments				Outcomes	
		GC	Conventional IS	Biologics	Other treatment	Primary outcomes	Secondary outcomes
**Pleuritis**							
1 observational study [60]	119 (127 episodes of pleural effusion including66 patients with lupus pleuritis, other etiology included TB, parapneumonic effusions, etc.)	**Number**: 93%;	ND	ND	**Type and Number**: − *NSAIDs*: 7%, 35%, and 100% of patients, respectively.	**Treatment response**: 94%; **non-response**: 1%; **unknown outcome**: 5%	**Recurrence**: 14% of patients
**Acute pneumonitis**							
1 case series [61]	5	**Number**: 100% of patients **Regimen**: GC combined with IS in 100% of patients **Dose**: high dose in 100% of patients	**Type and Number**: − *CYC (IV)*: 80% of patients. − *AZA*: 20% of patients.	**Type and Number**: ND	**Type and Number**: − *IVIG*: 60% of patients − *Ventilation*: 40% of patients	**Treatment response**: 60% of patients	**Mortality**: 40% of patients
**Diffuse alveolar hemorrhage**							
9 observational studies 3 case series [62–73]	398	**Number**: reported in all 12 studies, in 85.7%-100% of patients **Regimen**: 3 studies reported *GC alone* in 5.3%-88.2% of patients. 12 studies reported *GC combined with IS* and/or PE in 5.9%-100% of patients **Dose**: *high dose* in 100% of patients, including *Pulsed methyl-PNL* in 45%-100% of patients	**Type and Number**: − *CYC (IV)*: reported in 12 studies in 5.9%-100% of patients − *MMF*: reported in 1 study in 14.3% of patients − *AZA*: reported in 1 study in 26.3% of patients **Regimen**: *GC + CYC*,* GC + CYC+PE*,* GC + PE*,* GC + CYC+RTX + PE*,* GC + PE+IVIG*, etc.	**Type and Number**: − *RTX*: reported in 5 studies in 3.4%-45.7% of patients	**Type and Number**: − *PE*: reported in 12 studies in 5.9%-65.7% of patients − *IVIG*: 6 studies in 14%-71.3% of patients	**Mortality**: reported in 12 studies. 28.6%-61.8% of patients in 10 studies and 0% short-term mortality in 2 studies, with DAH-induced respiratory failure as the most common cause, followed by infection	**Infection**: reported in 2 studies in 72.6%-85.7% of patients
**Interstitial lung disease**							
1 prospective controlled cohort study, 5 observational studies, including 1 in SLE-PAH and 5 in CTD-PAH [74–79]	711 CTD-ILD patients, including 52 SLE-ILD patients	**Number**: reported in all 6 studies, in 94.6%-100% of patients **Regimen**: *GC alone* reported in 4 studies in 10%-80% of patients; *GC combined with IS/RTX* in 20%-100% of patients **Dose**: moderate to high dose in most of the studies. *Pulsed methyl-PNL* reported in 1 study in 40% of patients	**Type and Number**: − *CYC*: reported in 2 studies in 92.3%-100% of patients. 1 study compared oral with IV CYC − *MMF*: reported in 4 studies in 7.7%%-100% of patients. 1 study compared MMF with CYC. − *AZA*: reported in 1 study in 55.7% of patients. AZA was compared with MMF in this study. − *Tac*: reported in 1 study, in 21.4% of patients	**Type and Number**: − *Jak inhibitor*: reported in 1 study in 12.7% of patients.	**Type and Number**: − *anti-fibrotic agents*: reported in 2 studies. 1 prospective cohort study compared pirfenidone with placebo, and 1 case series reported pirfenidone use in 7.7% of patients.	**PFT improvement**,** including FVC and DLCO improvement**: − *Pirfenidone* vs. placebo: more improvement in pirfenidone group in SSc, IIM, and RA patients, not seen in SLE patients − *MMF* vs. *CYC*: more improvement in CYC group, but more AE also in CYC group − *AZA* vs. MMF: non-inferior efficacy in AZA group compared to MMF group. AE comparable in two groups. − *Oral CYC vs. IV CYC*: more improvement and less AE in IV CYC group	Comparison between AZA and MMF in 1 study: Mortality: 5.6% vs. 9.3%; Transplant rate: 3.7% vs. 2.3%); Respiratory hospitalization: 31.5% vs. 25.6%
**Shrinking lung syndrome**							
1 observational study and 4 case series [83–87]	56	**Number**: reported in all 5 studies, in 73%-100% of patients **Regimen**: all 6 studies reported *GC combined with IS* in 100% of patients **Dose**: *moderate to high dose* (30-60 mg/d) in most of the studies	**Type and Number**: − *AZA*: reported in 3 studies in 20%-57.1% of patients. − *MTX*: reported in 3 studies in 11.1%%-25% of patients. − *MMF*: reported in 2 studies in 15%-44.4% of patients. − *CYC*: reported in 1 study, in 28.6% of patients	**Type and Number**: − *RTX*: reported in 1 study in 55.6% of patients.	**Type and Number**: − *IVIG*: reported in 1 study in 11.1% of patients.	**Clinical improvement**: reported in 5 studies, in 11.1%-95% of patients. **PFT improvement**: reported in 5 studies in 22.2%-100% of patients	**Mortality**: reported in 1 study, in 20% of patients. **Imaging improvement**: reported in 1 study, in 40% of patients

Abbreviation: AE: adverse events; AZA: azathioprine; CTD: connective tissue disease; CYC: cyclophosphamide; DLCO: diffusing capacity of the lung for carbon monoxide; FVC: forced vital capacity; IIM: idiopathic inflammatory myositis; ILD: interstitial lung disease; IV: intravenous; IS: immunosuppressant; IVIG: intravenous immunoglobulin; methyl-PNL: methylprednisolone; MMF: mycophenolate; MTX: methotrexate; ND: not described; NSAIDs: non-steroids anti-inflammatory drugs; PE: plasma exchange; PFT: pulmonary function test; PNL: prednisolone; RA: rheumatoid arthritis; RTX: rituximab; SLE: systemic lupus erythematosus; SSc: systemic sclerosis; Tac: Tacrolimus

Apart from this study, one of the observational studies in the pericarditis section reported 30 episodes of pleuritis/ pleural effusion. The treatment response for pleuritis/pleural effusion was not reported separately from other forms of serositis. However, all patients’ serositis, including pleuritis, was resolved either with NSAIDs alone or with PNL or PNL plus IS [20, 24]. In addition, in the case series study in the pericarditis section reporting the efficacy of rilonacept, all patients also had pleuritis, which was completely resolved with treatment of the IL-1 inhibitor [27].

#### Acute lupus pneumonitis

One case series of five ALP cases was included in the present study (Table 3 and Supplementary Table 7) [61]. All five patients received a combination of high-dose GC and IS treatment, including one patient treated with high-dose GC followed by AZA and the other four with IV methyl-PNL and IV CYC. Two patients (40%, 2/5) responded well to treatment. However, three patients (60%, 3/5) developed respiratory failure despite GC pulse, IV CYC, and empirical antibiotics treatment. IVIG was added, and two received ventilatory support. One of these three severe patients subsequently improved, but the other two (40%, 2/5) died due to the ALP and secondary pneumonia.

#### Diffuse alveolar haemorrhage

We included 12 studies on DAH, including nine observational studies and three case series [62–73] with 398 patients in total (Table 3 and Supplementary Table 8). Intensive immunosuppressive therapy was used for almost all reported cases, including GC (12 studies), IS (12 studies), IVIG (6 studies), and RTX (5 studies). PE was also reported in all studies. In addition, prophylactic empirical antibiotic use was reported by five studies in 50%-94.1% of the patients [62, 64, 68, 71, 72], and ventilatory support was reported in nine studies [62–66, 68, 69, 72, 73].

##### Glucocorticoids

All but four patients (99.0%) in the 12 studies were treated with high-dose GC (≥ 1 mg/kg/d), including 71.1% of the patients who received pulse methyl-PNL as initial therapy. GC combined with IS and/or PE was the most common treatment regimen in 11 studies, accounting for 57.2%-100% of patients [63–73]. Only one observational study reported that most patients (88.2%) received high-dose GC alone without IS or other therapy. There were three GC treatment regimens in this study, including (i) oral prednisone (1 mg/kg/d); (ii) conventional pulse: IV methyl-PNL pulse (1000 mg/d for three days); (iii) ‘massive pulse’: IV methyl-PNL pulse (1000 mg/d for 4–8 days). The massive pulse regimen was used for those who did not respond to three-day pulses, with a 4-day interval between pulse courses. The mortality rate in this study was 61.8%, the highest among the 12 studies. Regarding the specific mortality by different GC regimens in this study, all nine patients (100%) on oral prednisone died; 69.2% of the patients (9/13) with conventional pulse therapy died; and 25% of the patients (3/12) on massive pulse regimen died [62].

Although on intensive immunosuppressive treatments, the mortality in these studies was 14.3%-61.8%, and most of the deaths were due to DAH-induced acute respiratory failure or infection [62–67, 69, 71–73]. Only two studies reported no short-term mortality, with all patients surviving through the DAH episodes [68, 70]. An observational study reported the influence of infection on patients with SLE-DAH, showing that 46.8% of the patients (58/124) with SLE-DAH developed bacterial infection after DAH occurrence, and 25.8% of the patients (32/124) developed fungal and/or viral infection. The patients with bacterial infection had significantly worse survival than those without infection or with non-bacterial infection, with 60.3% of mortality within 180 days (35/124). More than 50% of the patients received GC pulse and CYC therapy, regardless of infection status, and there was no significant difference in immunosuppressive or prophylactic antibiotic therapy between the bacterial, fungal/viral, and non-infection groups [71].

##### Conventional immunosuppressants

All 12 included studies reported conventional IS use in combination with GC, in 5.9%-100% of the patients [62–73]. CYC was the only conventional IS reported in eight studies [62, 65–69, 72, 73], and four studies reported other IS, including MMF, AZA, Tac, CsA, MTX, LEF [64, 67, 70, 71]. Four studies showed that the proportion of CYC use in survivors was higher than the deceased patients [63, 65, 66, 73], but one study showed an opposite result [72]. Two studies reported that 45.5% (5/11) and 57.1% (4/7) of the patients, respectively, were not responding to high-dose GC and CYC and required PE [68, 69].

##### Rituximab

Five studies reported RTX use for 3.4%-45.7% of the patients [65, 70–73], all published in the recent decade. The indications for RTX use were reported in one study, including recurrent DAH (75%, 3/4) or incipient DAH (25%, 1/4) as well as multi-organic flare (100%, 4/4). The schedule was 375mg/m^2^ fortnightly for two courses or weekly for four courses. These patients also received high-dose GC ± CYC while RTX was used, and all survived [65]. An observational study reported a high proportion of RTX use (45.7%) among 35 SLE-DAH patients (16/35), and in most cases (87.5%, 14/16) RTX was combined with GC, CYC, and PE. More RTX use was observed in the survivors compared to the dead patients, but it did not reach statistical significance (48.1% vs. 37.5%) [73].

##### Plasma exchange

PE was reported in all 12 studies, and overall, 37.9% of the patients received PE. Three studies reported the number of courses of PE, which varied between 1 and 28 courses for each patient [62, 63, 68, 73]. PE was combined with GC and/or IS as a first-line therapy or when DAH was persistent despite high-dose GC and CYC. Although the specific treatment response to PE was difficult to identify from the combination regimens, two studies reported that PE was used for the patients who did not respond to high-dose GC and CYC, and 100% (5/5) and 75% (3/4) of these patients on rescue PE therapy survived, respectively [68, 69]. However, one study showed that patients on PE alone had a significantly lower survival and shorter time to death than patients on triple therapy (CYC + RTX+PE) (time to death: median 14 vs. 162 days, *p* = 0.0026). Both groups also received high-dose GC [73].

##### Intravenous immunoglobulin

Six studies reported IVIG use for DAH in 14.3%-71.3% of patients. Most of these studies did not report specific outcomes of treatment with IVIG [64, 70–73]. Two studies showed that the proportion of patients who used IVIG was slightly higher among survivors than among those who died [66, 73].

##### Ventilation

Ventilatory support was reported for a high proportion (36.3%-100%) of patients in most of these studies [62–66, 68, 69, 72, 73]. In addition, the patients requiring ventilation support had higher mortality than those not on this therapy in five studies [62, 65–67, 73].

In summary, DAH is a serious condition with a high risk of respiratory failure and ventilatory requirements, and high mortality. Pulse methyl-PNL was commonly used and could be repeated if a conventional three-day pulse was non-responsive. Most patients were treated with a combination of GC and IS, and CYC was the most commonly used and studied IS. PE was also commonly used in these studies and showed benefits in some, especially for those with a limited response to GC and IS. RTX was used more in the recent decade, and limited evidence showed its effectiveness, especially patients with recurrent DAH. In addition, empirical antibiotics were often used concomitantly with immunosuppressive therapy. Despite aggressive treatment, patients with this condition had high recurrence and mortality (14.3%-61.8%) in most of the studies, especially the study wherein most patients were on GC only [62]. DAH itself with secondary respiratory failure and infection were two main causes of death.

#### Interstitial lung disease

Six studies with 711 CTD-ILD patients, including 52 SLE-ILD patients, were included in this review (Table 3 and Supplementary Table 9). Five of them reported different types of CTD-ILD patients [74–78], and only one study focused on SLE-ILD patients [79]. One prospective controlled cohort study evaluated the efficacy of pirfenidone combined with IS in CTD-ILD patients [74], and five observational studies reported the effectiveness of immunosuppressive treatment for CTD-ILD or SLE-ILD [75–79]. Four of the six studies did not limit the types of ILD, and the diagnosis of ILD was typically made on the integration of clinical symptoms, pulmonary function tests, and computed tomographic (CT) scans [74, 75, 77, 78]. However, one observational study only included patients with fibrotic ILD, which was defined as ‘the presence of reticulation with traction bronchiectasis or subpleural honeycombing’ [76]. The prospective controlled cohort study and three of the observational studies set comparators for treatment, and FVC% and DLCO% change were the most used outcome [74, 76–78].

##### Glucocorticoids

All six studies reported GC use in combination with IS. The median dose of prednisone was low to moderate in three studies (median dose 15–20 mg/d) [74–76] and moderate to high (20-80 mg/d) in two studies [77, 78]. The observational study focusing on fibrotic CTD-ILD patients showed that combining low to moderate doses of GC with IS (AZA or MMF) could help stabilize pulmonary function [76]. The other two observational studies in CTD-ILD patients demonstrated that using moderate to high doses of GC in combination with IS (MMF or CYC) significantly improved pulmonary function compared to baseline, especially for patients with non-UIP pattern [75, 77], and stabilized the pulmonary function decline for the subgroup with UIP pattern [75].

##### Conventional immunosuppressants

All four observational studies in CTD-ILD reported on IS treatment, including MMF, AZA, and CYC, and the results suggested that these ISs are effective for CTD-ILD, although SLE-specific outcomes were difficult to separate out. One study compared AZA (median 125 mg/d) with MMF (median 2000 mg/d) in treatment for fibrotic CTD-ILD patients, including SLE. Low-to-moderate doses of GC were combined in both groups. Although both AZA and MMF treatments were associated with pulmonary function stability over four years, the AZA group showed a marginal improvement compared with the MMF group. These findings remained unchanged after adjustment for some coexisting factors, including the UIP pattern and concurrent prednisone dose. The overall adverse outcomes (death, transplant and respiratory hospitalization) and therapy discontinuation rates were comparable between the two groups. But it is worth mentioning that the median percentage area of fibrosis changes in HRCT in the two arms was 15%-20%, indicating the patients included had mild-moderate disease [76]. One study retrospectively compared MMF (500 mg twice daily) with oral CYC (50 mg once every other day) in CTD-ILD patients, including SLE. Moderate to high-dose GC was combined in both groups. At the end of the study (2 years for the MMF group and 1 year for the CYC group), significant improvements in FVC% and DLCO% were observed in both groups, with greater improvement in FVC% in the CYC group. However, AE and all-cause mortality were observed more frequently in the CYC group [77]. Another study retrospectively compared oral CYC (1-2 mg/kg/d for one year, every 2 weeks of treatment followed by one week of discontinuation) with IV CYC (400-600 mg fortnightly×3 months and then prolonged to monthly for one year) in CTD-ILD patients, including SLE. The two groups received comparable doses of prednisolone and had comparable HRCT and lung function at baseline. They found that the IV CYC group showed significantly more improvement in HRCT Warrick score, FVC, DLCO, and 6MWD in 6 months, than the oral CYC group. Additionally, the cumulative CYC dose and the total AEs rate were lower in the IV group than in the oral group [78].

##### Anti-fibrotic agents

The prospective controlled cohort study evaluated the efficacy of pirfenidone combined with immunosuppressive therapy in CTD-ILD, with IIM, SSc and RA as the major CTD types and fewer cases in other CTD types, including SLE. Patients received a stable dose of GC and/or IS for at least four weeks before being assigned to either the pirfenidone group or a control group for 24 weeks. In the results, the benefits of pirfenidone in pulmonary function improvement were not confirmed in SLE-ILD patients, although there were some benefits in SSc, IIM, and RA-related ILD patients [74].

RCTs for anti-fibrotic agents, including nintedanib and pirfenidone, were excluded from this review because no SLE-ILD patients were included in these trials [80–82].

In summary, the safety and effectiveness of the combination therapy of GC and IS, including MMF, CYC, and AZA, were confirmed in CTD-ILD patients. Patients with fibrotic ILD and patients with UIP patterns can also benefit from immunosuppressive therapy, which may stabilize the disease. AZA showed comparable AE and non-inferiority to MMF from one study [76]; and CYC presented superiority in improving FVC%, but was associated with higher AE and mortality compared to MMF [77]. IV CYC regimen showed superior efficacy and fewer adverse effects compared to oral CYC [78]. Based on very limited evidence, the efficacy of pirfenidone was not confirmed in SLE-ILD patients. The mortality of SLE-ILD patients was reported as 5.6%-9.3% and 1%-6%, respectively, in two studies [76, 77].

#### Shrinking lung syndrome

Five studies on SLS were included in this review, including one observational study and four case series with a total of 56 patients (Table 3 and Supplementary Table 10) [83–87]. SLS was confirmed mainly by PFT showing a restrictive ventilatory defect and radiological evaluation showing an elevated hemidiaphragm. Immunosuppressive therapy, including GC (5 studies), IS (5 studies), and RTX (2 studies), was primarily reported as a treatment for SLS in these studies. Other treatments, such as antimalarials (2 studies), IVIG (1 study), and colchicine (1 study) were also reported. In addition, *β-*agonists and theophylline were used as adjuvant therapy in four studies. No comparator was included in these studies.

##### Glucocorticoids

All studies reported GC use in 73%-100% of patients. Most patients took oral GC with an average initial dose of 30-60 mg/d, and only two patients with severely impaired pulmonary function received pulse GC [85]. In terms of regimen, GC alone was used as initial treatment for 10–80% of patients from four studies [83, 85–87], and 53.4%-100% of patients used a combination of GC with IS/RTX. In response to these treatments, 25%-95% of the patients had clinical improvement [83–85, 87], and 33.3%-87.5% had spirometric improvement [83–87]. Only one study reported 20% (4/20) mortality, with one patient dying of respiratory failure and the other three dying of other causes [83]. In the total of eleven cases from three studies that were managed solely with moderate to high doses of GC, 63.6% (7/11) showed significant improvements in clinical symptoms and pulmonary function tests, and 18.2% (2/11) stabilized [85–87].

##### Conventional immunosuppressants

IS medications, including AZA, MMF, CYC, and MTX, were presented for 20%-100% of patients in the five studies as first-line combination therapy with GC, rescue therapy, or as a steroids-sparing agent [83–87]. AZA or MMF were more commonly used IS, and most of the IS use was combined with GC ± RTX, with an improvement frequency varying from 33.3%-95% [83–87].

##### Rituximab

There were two included studies reporting RTX use in combination with GC ± IS as a rescue or first-line therapy for seven SLS cases in total, and 57.1% of the patients (4/7) showed improvement in clinical, spirometric, and/or imaging aspects, and 42.9% (3/7) stabilized [86, 87].

##### Intravenous immunoglobulin

IVIG use was only reported in one study for one patient, in combination with GC, MMF, and RTX. This patient’s restrictive ventilatory defect was stabilized by the treatment [86].

##### Colchicine

Colchicine was used solely for one patient in one study, leading to clinical improvement in pleuritic chest pain but not in dyspnea, as well as stabilization of PFT and radiology.

##### β-agonists and theophylline

Four studies reported β-agonists and/or theophylline as adjuvant therapy to immunosuppressive drugs for a proportion of patients (13.3%-77.8%) [83, 84, 86, 87]. Most patients were treated with either β-agonists or theophylline, but one patient was on a combination of the two, and all of them attained improvement or stabilization. No indication of β-agonist or theophylline use was reported in these studies.

In summary, GC alone or combined with IS was the primary treatment for SLS in the above studies. Moderate to high-dose (30-60 mg/d) GC was commonly prescribed, and AZA and MMF were used more often than other ISs. RTX has been reported in more recent studies and showed promising efficacy as a rescue or first-line combination therapy with GC and IS. β-agonists and/or theophylline were also commonly reported as adjuvant therapy to immunosuppressive drugs. The treatment outcomes were promising, with an overall clinical improvement rate of 57.1%-100%. However, spirometric improvement was less frequent than clinical improvement, and most patients could only achieve partial improvement in restrictive ventilatory defects. Mortality was rarely reported.

#### RCTs for new SLE treatment

Some RCTs for new SLE treatment reported inclusion of patients with cardiopulmonary involvement, such as the phase II/III EXPLORER trial for RTX [88], phase III BLISS 52 and 76 trials for belimumab (BEL) [12, 13], phase III SLE-BRAVE-I and II trials for baricitinib [89, 90], phase II PAISLEY trial for deucravacitinib [91], phase III EMBODY 1& 2 trials for epratuzumab [92, 93], and phase III ALLEVIATE 1, 2, and open-label trials for epratuzumab [92, 93]. However, we found that none of these trials stated which specific cardiopulmonary manifestations were present. We only included the ALLEVIATE trial for epratuzumab in this review, as this is the only trial which reported treatment response of cardiopulmonary manifestations. The ALLEVIATE trials were discontinued prematurely because of interruption in drug supply. The pooled exploratory data showed a greater change in overall BILAG score at week 48 from baseline in the epratuzumab group than in the placebo group. However, this did not identify superiority of epratuzumab in the cardiopulmonary domain (Supplementary Table 11) [93].

## Discussion

**Fig. 2:**
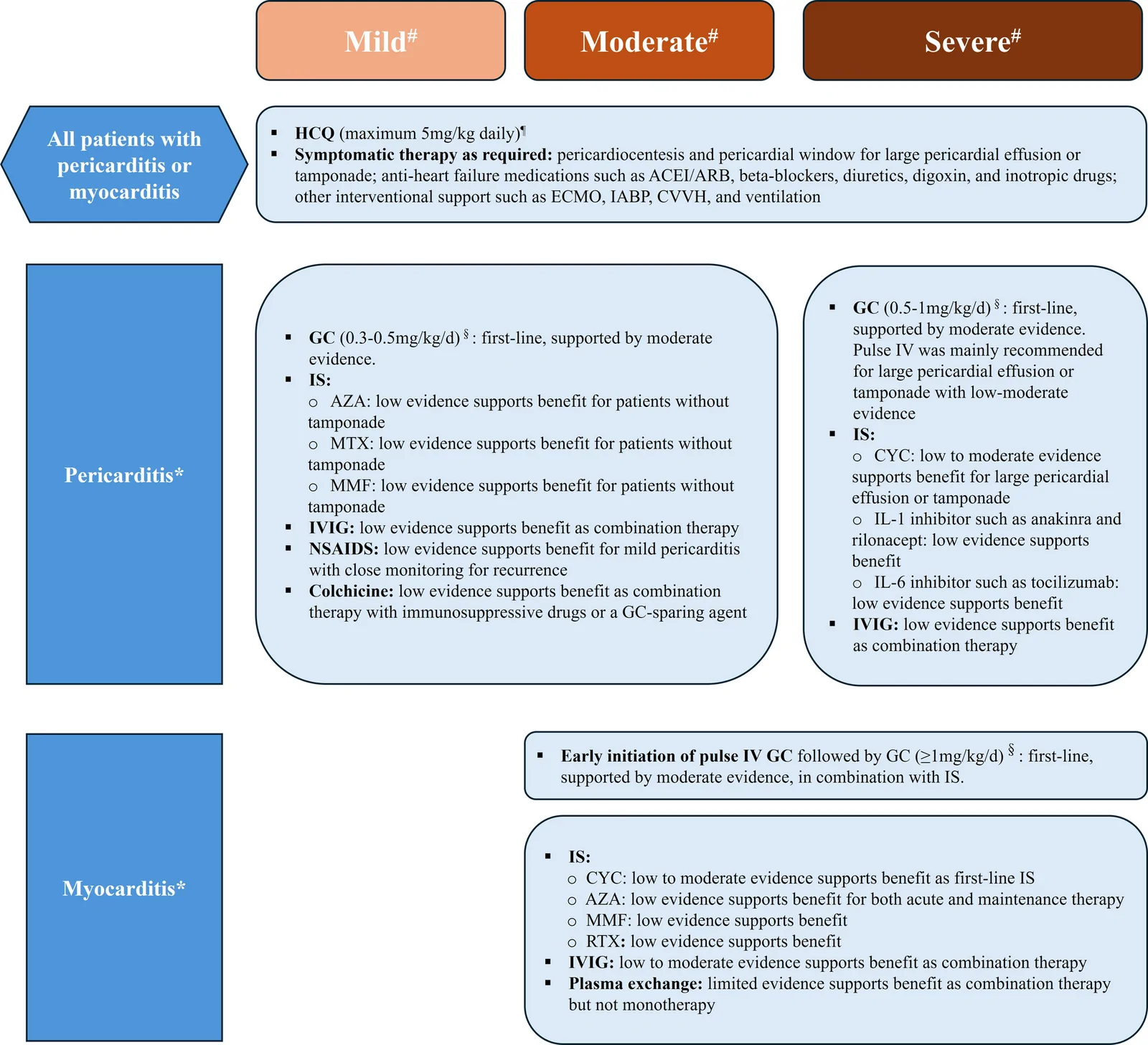
Treatment algorithms for pericarditis and myocarditis in SLE patients. # **Mild**: new onset or persistent mild pericarditis. **Moderate**: new onset, persistent, or worse manifestations of moderate pericarditis and mild myocarditis; **Severe**: new onset, persistent, or worse manifestations of cardiac tamponade and myocarditis with cardiac failure. ¶ HCQ is recommended as background/baseline therapy for all SLE patients unless contraindicated, according to current guidelines. §The historical data often used uniform, higher initial GC doses. However, the current best practice requires stratifying GC dosing by disease severity. * Very low evidence also supports the benefit from BEL, TCZ, and ANR. Abbreviation: ACEI/ARB: angiotensin-converting enzyme inhibitor/angiotensin receptor blocker; ANR: anakinra; AZA: azathioprine; BEL: belimumab; CVVH: continuous veno venous hemofiltration; CYC: cyclophosphamide; ECMO: extracorporeal membrane oxygenation; GC: glucocorticoids; HCQ: hydroxychloroquine; IABP: intra-aortic balloon pump counterpulsation; IS: immunosuppressant; IV: intravenous; IVIG: intravenous immunoglobulin; MMF: mycophenolate; MTX: methotrexate; NSAIDS: nonsteroidal anti-inflammatory drugs; PO: per os; RTX: rituximab; SLE: systemic lupus erythematosus; TCZ: tocilizumab

**Fig. 3:**
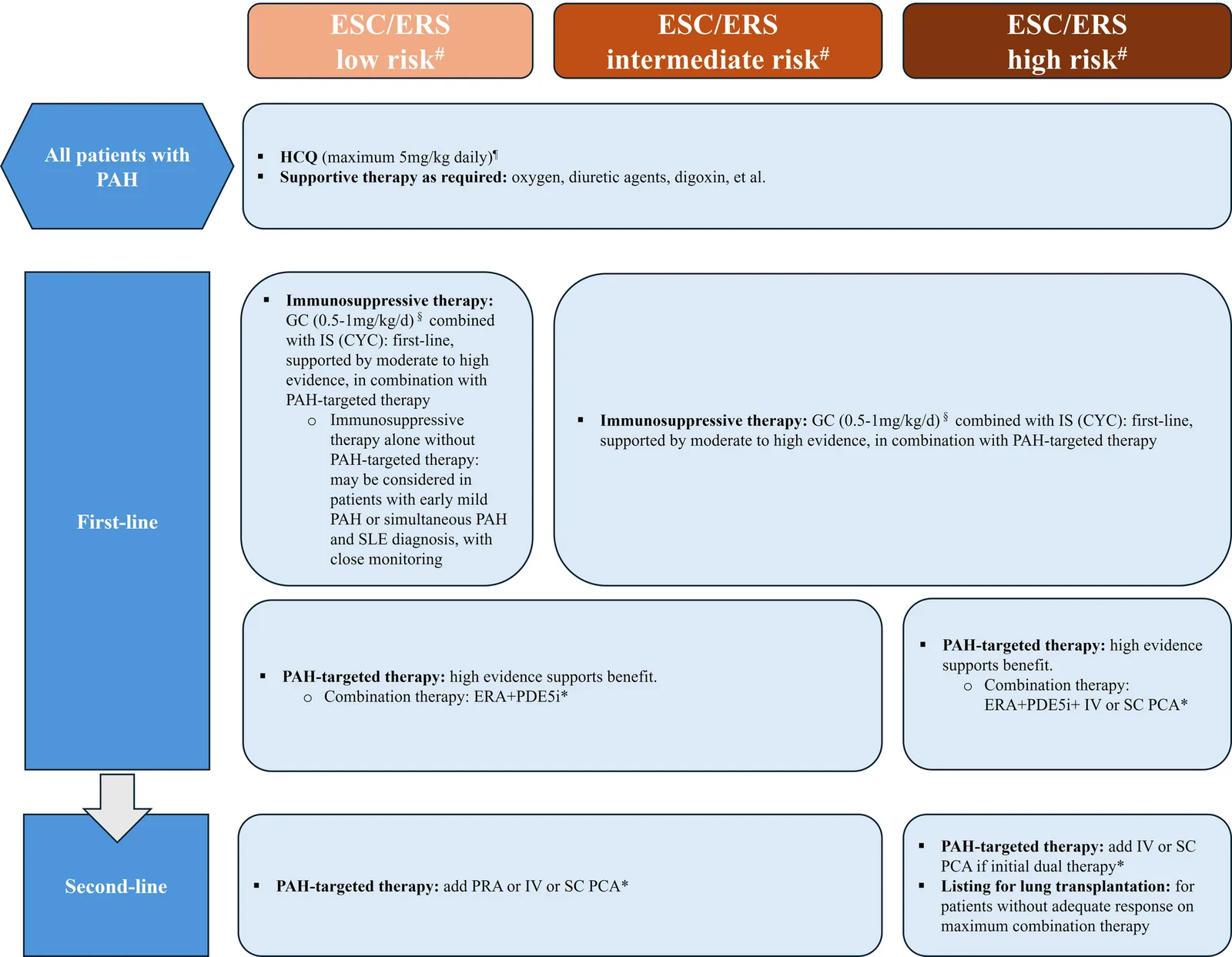
Treatment algorithms for PAH in SLE patients. # The risk stratification is based on a multiparametric approach according to the 2022 ESC/ERS PAH recommendations [105] and the PAH treatment algorithm from the 7th World Symposium on PH [106]. The comprehensive model includes clinical symptoms, WHO-FC, 6MWD, echocardiography, cardiac MRI, and hemodynamics, and the simplified model includes WHO-FC, 6MWD, and BNP/NT-proBNP [105]. * Combination strategy refers to the literature review, the 2022 ESC/ERS Guidelines for the diagnosis and treatment of PH [105], and the PAH treatment algorithm from the 7th World Symposium on PH [106]. ¶ HCQ is recommended as background/baseline therapy for all SLE patients unless contraindicated, according to current guidelines. §The historical data often used uniform, higher initial GC doses. However, the current best practice requires stratifying GC dosing by disease severity. Abbreviation: CYC: cyclophosphamide; ESC: European Society of Cardiology; ERA, endothelin receptor antagonist; ERS: European Respiratory Society; GC: glucocorticoids; HCQ: hydroxychloroquine; IV: intravenous; PAH: pulmonary arterial hypertension; PCA: prostacyclin analogs; PDE5i, phosphodiesterase 5 inhibitor; PH: pulmonary hypertension; PO: per os; PRA, prostacyclin receptor agonist; PVR: pulmonary vascular resistance; SC: subcutaneous; sGCs, soluble guanylate cyclase stimulator; SLE: systemic lupus erythematosus

**Fig. 4:**
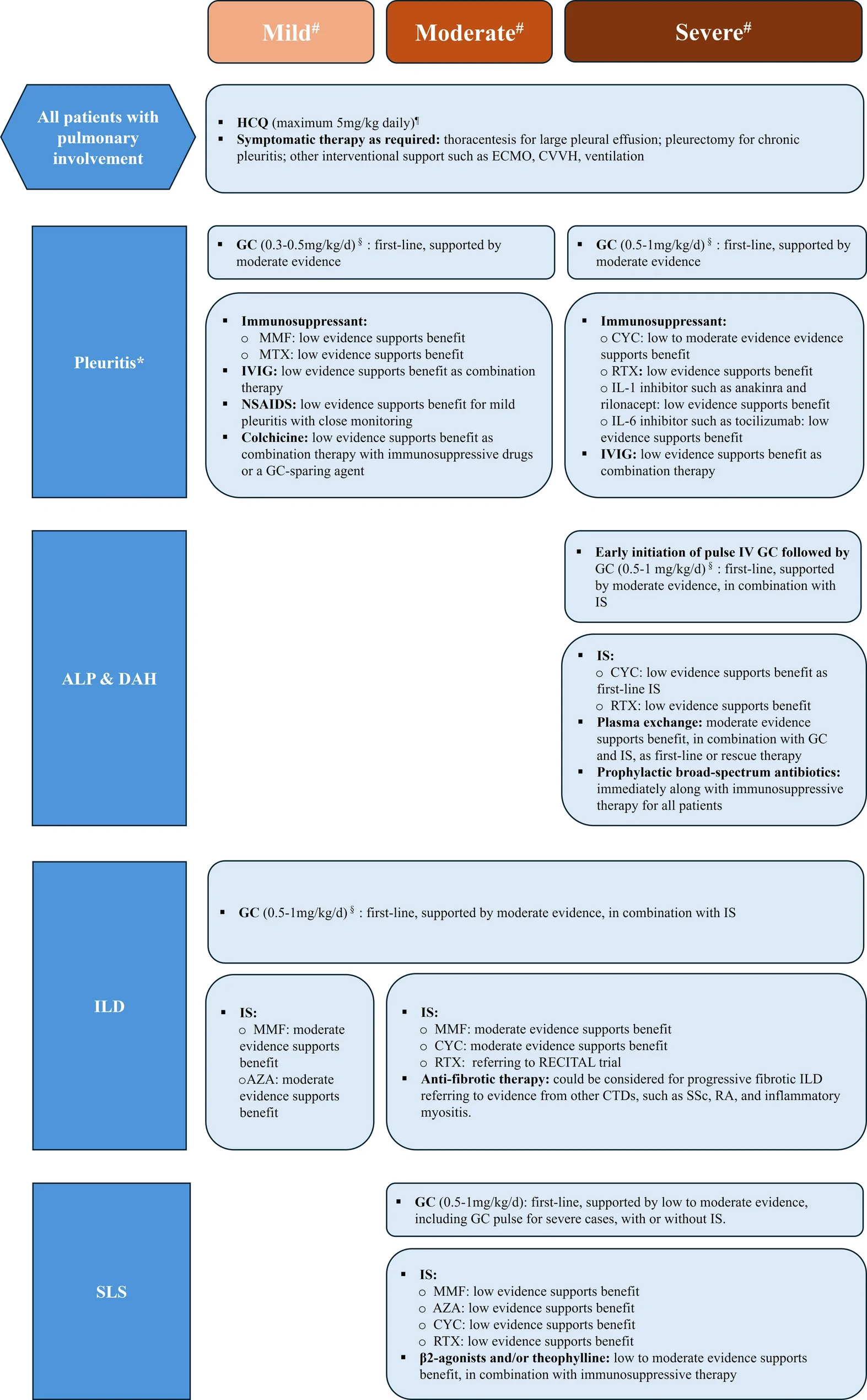
Treatment algorithms for pleuritis, ALP, DAH, ILD, and SLS in SLE patients. # **Mild**: new onset or persistent mild pleuritis or ILD. **Moderate**: new onset, persistent, or worse manifestations of moderate pleuritis, moderate ILD, and mild SLS; **Severe**: new onset, persistent, or worse manifestations of pleural effusion with dyspnoea, SLS with dyspnoea, DAH, ALP, and severe or progressive ILD. ¶ HCQ is recommended as background/baseline therapy for all SLE patients unless contraindicated, according to current guidelines. §The historical data often used uniform, higher initial GC doses. However, the current best practice requires stratifying GC dosing by disease severity. * Very low evidence also supports the benefit from BEL and TCZ. Abbreviation: ALP: acute lupus pneumonitis; AZA: azathioprine; BEL: belimumab; CTD: connective tissue disease; CVVH: continuous veno venous hemofiltration; CYC: cyclophosphamide; DAH: diffuse alveolar haemorrhage; ECMO: extracorporeal membrane oxygenation; GC: glucocorticoids; HCQ: hydroxychloroquine; ILD: interstitial lung disease; IS: immunosuppressant; IV: intravenous; IVIG: intravenous immunoglobulin; MMF: mycophenolate; NSAIDS: nonsteroidal anti-inflammatory drugs; PO: per os; RTX: rituximab; SLE: systemic lupus erythematosus; SLS: shrinking lung syndrome; TCZ: tocilizumab

This study systematically and comprehensively reviewed the literature on the treatment of inflammatory cardiopulmonary manifestations of SLE, including treatment indications, medication types and doses, treatment regimens, efficacy, patient outcomes, and side effects. We found that for most manifestations, GCs were used in the acute setting, with higher doses generally used when patients had more severe disease. Various IS were described; for more severe manifestations such as myocarditis, PAH, and DAH, CYC was most commonly used; others included AZA and MMF. In refractory disease, low-to-moderate evidence supported RTX as second-line therapy for some conditions, such as pericarditis, LM, DAH, and SLS. Other therapies, such as PE, were found to be efficacious for certain manifestations such as DAH. Regarding the treatment of PAH in SLE patients, PAH-targeted therapy can provide additional benefits to immunosuppressive therapy. For ILD in SLE patients, immunosuppressive therapy can improve the disease or stabilize progression. Based on the evidence we have reviewed, several therapeutic strategies for managing inflammatory cardiac and pulmonary manifestations of SLE are summarized in Figs. 2, 3 and 4.

Pericarditis is the most common cardiac manifestation of SLE, with a prevalence of 11–54%. Some autopsy studies have shown that up to 83% of SLE patients have pericardial involvement [3], but cardiac tamponade rarely occurs with an incidence of 1.0%-2.5% [94]. According to the data we have assessed, moderate evidence supports moderate-to-high dose (0.5-1 mg/kg/d) GC as first-line therapy for most cases with lupus pericarditis [19, 20, 22–26]. GC pulse or a combination of GC with IS, such as CYC, was mainly recommended for large pericardial effusion or tamponade. In the 2015 guidelines of the European Society of Cardiology (ESC) for pericardial diseases, IL-1 inhibitors such as ANK were recommended as a third-line therapy option for refractory GC-dependent recurrent pericarditis [95]. For lupus pericarditis, only one case report showed effectiveness of ANK in a refractory case [96]. In 2020, a phase-III RCT demonstrated that rilonacept led to rapid resolution of idiopathic recurrent pericarditis [97], and the FDA has approved rilonacept to treat recurrent pericarditis and reduce the risk of recurrence since 2021. The case report included in the present study demonstrated the effectiveness of rilonacept in refractory SLE-pericarditis cases [27]. Based on the above evidence, rilonacept and ANK could be considered in refractory SLE-pericarditis patients. Colchicine could be prescribed as first-line combination therapy with immunosuppressive drugs or as a GC-sparing agent [26]. Although a positive response to treatment is likely when immunosuppressive agents are utilized in pericarditis, close monitoring is needed due to a high recurrence rate. In addition to the studies included, some new therapies were reported in some case reports. For example, Kamata et al. and Ocampo et al. both reported that tocilizumab, a recombinant monoclonal antibody against the interleukin-6 (IL-6) receptor, successfully treated refractory pericarditis in SLE patients [98, 99]. BEL, a recombinant monoclonal antibody against B Lymphocyte Stimulator (BlyS), also had some evidence in treatment for lupus serositis, although there was no direct evidence for pericarditis in particular. For example, in the original clinical trials of BEL, 3% of the enrolled patients presented with serositis [12]. In addition, one case from Mukkera et al. showed the efficacy of BEL for refractory peritoneal serositis [100], and one case from Jüptner et al. showed its effectiveness for refractory lupus pleuritis [101].

LM is not a common manifestation, presenting in around 7–10% of SLE patients [6], but it is potentially life-threatening. According to our study, RCTs for treatment in LM are lacking. Based on the currently available low to moderate evidence from observational studies and case series, we recommend initial pulse methyl-PNL (500-1000 mg/d for three days) followed by high-dose GC (≥ 1 mg/kg/d) for all patients with LM, in combination with IS±IVIG. Repeated pulses could be considered in refractory cases. Among IS agents, IV monthly CYC has the most evidence, followed by oral CYC, AZA, and MMF. RTX with routinely recommended regimens of 100 mg fortnightly × 2 or 375 mg/m2 weekly ×4, was recommended as second-line therapy for refractory cases based on limited evidence [31]. In addition to immunosuppressive treatment, anti-heart failure medications such as ACEI/ARB, beta-blockers, diuretics, digoxin, and inotropic drugs, as well as interventional support such as ECMO, IABP, CVVH, and ventilation, should be considered if required [28–30, 32–35].

PAH is a severe complication that has a poor prognosis without prompt treatment. The reported prevalence of PAH in the SLE population was approximately 2%-5% [4, 5, 102, 103]. Immune and inflammatory mechanisms play a crucial role in the development of PAH in SLE patients. Vasculitis or capillaritis of the pulmonary vessels has been shown to lead to vascular remodelling, damage, and subsequent elevated pulmonary vascular resistance [104]. Treatments for CTD-PAH, including SLE-PAH, mainly include immunosuppressive therapy and PAH-targeted therapy [37–59]. The present study reviewed 23 studies, including 8 RCTs, of which 13 were conducted in CTD-PAH patients rather than SLE-PAH alone. Therefore, more high-quality evidence for specific SLE-PAH treatment is still needed. Based on the available evidence, we recommend immunosuppressive therapy combined with PAH-targeted therapy for SLE-PAH patients. Immunosuppressive therapy alone without PAH-targeted therapy might be considered, with close follow-up, in patients who are at an early stage of PAH, develop PAH simultaneously with CTD or within a short interval, and have ‘low risk’ based on stratification. The most common immunosuppressive therapy regimen was a combination of moderate-to-high dose GC (0.5-1 mg/kg/d) and IV CYC. GC pulse could be considered in severe cases. There was very limited evidence of the benefits of other IS such as AZA, MMF, MTX, and Tac. Regarding PAH-targeted medications, while monotherapy could benefit SLE-PAH patients, initial combination therapy targeting different pathways could yield better outcomes. According to the updated ESC and the European Respiratory Society (ERS) PAH management guidelines [105] and the updated treatment algorithm from the 7th World Symposium on PH in 2024 [106], the PAH-targeted treatment strategy should be based on risk stratification using a multidimensional risk assessment approach. The risk-stratification tools recommended by ESC/ERS have shown prognostic value in CTD-PAH patients, including SLE-PAH patients [107–109]. Based on these guidelines and our review of the evidence, we recommend stratifying the treatment strategy by prognostic risks. Patients with low or intermediate risk PAH are recommended to start with dual therapy, and patients with high-risk PAH are suggested to initiate triple therapy [105]. In addition to the medications targeting the three classic pathways, sotatercept is a novel first-in-class activin signaling inhibitor, approved for the treatment of PAH in 2023. By restoring the balance between pro- and anti-proliferative signaling in pulmonary vasculature, the safety and efficacy of sotatercept in PAH has been assessed in multiple clinical trials [110–114]. According to the PAH treatment algorithm from the 7th World Symposium on PH, sotatercept could be considered as a second-line add-on therapy for patients who do not reach a low-risk status after initial treatment with dual oral PAH therapy [106]. Although CTD-PAH patients were included in these sotatercept trials, the reports did not present specific results in CTD patients [115]. They also did not report whether SLE-PAH patients were included or how many of them were included. Therefore, these trials were not included in the present review. Overall, there is a lack of strong evidence, particularly RCTs, specifically targeting treatment for SLE-PAH.

Pleuritis is the most common pulmonary manifestation in SLE patients [5]. The prevalence of this was reported to be 14%-43% among SLE patients [5, 116, 117]. Based on very limited and low-level evidence and evidence from pericarditis, we recommend that most patients with lupus pleuritis are managed with moderate to high-dose (0.5-1 mg/kg/d) GC after excluding the possibility of infectious pleuritis and other causes. Close follow-up with maintenance therapy should be conducted due to the high recurrence rate. Some previous case reports with less than five cases showed positive results for IS such as CYC [20], MMF [118], and MTX [119], biologics, such as RTX [120], tocilizumab [99], and BEL [101], and IVIG [121, 122] for refractory lupus pleuritis. The above-mentioned case series study of rilonacept for refractory SLE-pericarditis also demonstrated its effectiveness for refractory pleuritis [27].

ALP is a rare but potentially life-threatening pulmonary manifestation in SLE patients, with a prevalence of 1%-4% [5, 61] and up to 50% of mortality [123]. Respiratory failure, pulmonary haemorrhage, secondary infection, and chronic interstitial pneumonitis are common complications of ALP [61, 123]. Based on limited evidence, we recommend initiating high-dose (≥ 1 mg/kg/d) GC, including pulse methyl-PNL combined with IS, especially IV CYC, for patients with ALP after excluding infections. IVIG could also be considered as first-line combination therapy or for refractory cases. Immediate empirical broad-spectrum antibiotics are also highly recommended, given the high infection incidence. Beyond this case series, some case reports showed the efficacy of PE in fulminant ALP [124–126].

DAH is another rare but potentially catastrophic manifestation of SLE. The reported incidence varied from 0.5% to 9.0%, and the mortality in most studies was above 30% with a higher level at 60%-80% [5, 61]. According to current low-moderate evidence, we advocate early management with the combination of methyl-PNL pulse therapy and IV CYC, along with empirical antibiotics. PE should be considered in combination with GC and CYC, especially for patients with limited response to conventional therapy, and RTX is also potentially beneficial for refractory and recurrent patients. Patients who experience severe respiratory failure and require respiratory and circulatory support, such as mechanical ventilation and ECMO, are at higher risk of poor prognosis [62, 65–67, 73]. Six included studies reported IVIG therapy for DAH patients, and some of them showed potential benefits [66, 73]. However, regarding the efficacy of IVIG for DAH, there are contradictory reports. A case report from Claridge et al. reported that the deterioration of DAH in an SLE patient was associated with IVIG use. The fluid load from the IVIG infusion was suspected of exacerbating the pulmonary haemorrhage, and caution was suggested for treatments such as IVIG, that requires a large amount of fluid load for DAH [127].

ILD is a common pulmonary manifestation in CTD patients, with different progression patterns, such as nonprogressive, chronically progressive, or rapidly progressive. The prevalence of ILD in SLE patients was reported to be 2%-9% [4, 5, 7], which was lower than that in other CTDs, such as SSc, PM/DM, or RA. Different histological patterns of ILD, such as nonspecific interstitial pneumonia, UIP, organizing pneumonia, lymphocytic interstitial pneumonia, follicular bronchiolitis, and nodular lymphoid hyperplasia, can be seen in CTD patients, with most of them being reported in SLE patients. In general, severe cases and rapidly progressive ILD are unusual in SLE patients [7, 128]. There have been limited RCTs conducted for treating CTD-ILD, with most data from SSc-related ILD [80–82]. The present study summarized four studies in the treatment of CTD-ILD, all of which involved SLE-ILD patients [74–77]. Based on the available moderate evidence, a combination of moderate to high doses (0.5-1 mg/kg/d) GC and IS, such as MMF, CYC, and AZA, is advocated, especially for those patients with non-UIP patterns. CYC might have higher AEs, such as infections, and consequently higher mortality than the other IS agents [77]. The newly published international expert consensus on the management of rare SLE manifestations did not recommend AZA for the treatment of SLE-ILD [11]. However, based on the evidence from one study in our review showing that AZA had non-inferior efficacy to MMF in stabilising lung function progression in mild-moderate fibrotic CTD-ILD patients [76], we recommended that AZA could be considered as a treatment option in mild SLE-ILD patients. Beyond this evidence, a recently published RCT (RECITAL trial) compared the efficacy and safety of RTX and CYC for severe or progressive CTD-ILD, including IIM, SSc, and MCTD patients, and found that RTX was not inferior to CYC to treat patients with CTD-ILD and was associated with fewer AE [129]. This trial was excluded from the present study because no SLE patients were enrolled. However, referring to this evidence, RTX could also be considered a therapeutic alternative to CYC or a second-line therapy for progressive ILD in SLE patients. Regarding anti-fibrotic agents, most published RCTs of anti-fibrotic agents in ILD, such as the INBULD and RELIEF trials, included a proportion of CTD patients with SSc, RA, and IIM and suggested potential benefits for progressive fibrotic ILD [80–82]. However, no SLE patients were included in these trials. In the absence of evidence for SLE-ILD, anti-fibrotic agents may be discussed on an individual case basis for SLE patients with progressive fibrotic ILD.

SLS is a rare manifestation of SLE, with a prevalence of approximately 0.5–1.5% [4, 5, 86]. The pathophysiology of SLS remains poorly understood, although various mechanisms, such as respiratory muscle weakness, diaphragmatic myopathy, and phrenic nerve dysfunction, have been investigated or postulated [4, 7]. In terms of treatment for SLS, based on low to moderate evidence, moderate-to-high dose (0.5-1 mg/kg/d) GC alone or in combination with IS, such as MMF, AZA, CYC, and MTX, are recommended. GC pulse therapy could also be considered in severe cases. RTX could be used as rescue therapy for refractory cases or first-line combination therapy with GC and conventional IS. In addition to immunosuppressive treatment, β2-agonists and/or theophylline could be added as adjuvant therapy to improve lung capacity by strengthening the diaphragm muscle. Besides the evidence from the included studies, a recent case report demonstrated the effectiveness of sequential therapy with RTX and BEL in a lupus-related SLS, which was refractory to methyl-PNL pulse therapy [130].

There are several strengths in the present study. To our knowledge, this is the first comprehensive, systematic and detailed literature review of the treatment of inflammatory cardiopulmonary manifestations of SLE. Here, we have reported on the medications and treatment regimens and reviewed the treatment response and prognosis of patients with these conditions. Secondly, we have provided specific treatment recommendations and pragmatic treatment algorithms for different conditions. Thirdly, our review has identified gaps in the treatment of inflammatory cardiopulmonary manifestations of SLE and highlighted the paucity of high-quality studies in most cardiopulmonary conditions in SLE.

There are also some limitations. Firstly, the efficacy of individual therapies is hard to assess in most included studies, as most cases were managed with multiple treatment modalities. Secondly, there is a lack of high-quality data. Most studies were observational or case series, conferring a high risk of publication bias. Thirdly, the number of included studies was very limited for some conditions, such as pleuritis and ALP, as most searched studies in these areas were case reports with < 5 cases. Fourthly, for PAH and ILD, most studies targeted a broad group of CTD patients rather than SLE patients alone and did not report separate treatment regimens and responses of SLE patients. Fifth, due to the high heterogeneity across manifestations and different studies, it is difficult to make specific recommendations. Finally, more than 50% of the studies included in this review were published more than 10 years ago. The treatment strategies for SLE have changed over time. We can see from the included studies that an initial high dose of GC (such as a 1 mg/kg/day or 0.5-1 mg/kg/day strategy) was commonly used, especially in studies published in earlier eras. However, current guidelines recommend a lower initial GC dose and rapid tapering to a low dose for maintenance [131]. This strategy change in GC dosing was based on evidence of long-term damage from GC use, equivalent efficacy of lower GC doses, and enhanced use of other GC-sparing agents. This ‘temporal heterogeneity’ in GC dosing is a limitation of the present study, and the contemporary applicability of the pooled dosing findings should be adjusted in keeping with modern treatment strategy and specific circumstances. In addition, some new medications have been approved and used in SLE patients in the recent decade, such as BEL and anifrolumab. However, the evidence was very limited regarding the use of these newly approved medications in patients with cardiopulmonary manifestations. In addition, most of the included studies did not report the use of antimalarials. Even in those studies reporting antimalarial use, it was mostly used as baseline or combination therapy, and its separate efficacy could not be assessed.

## Conclusion

The present study systematically reviewed the literature and provided a comprehensive review of available evidence in the management of inflammatory cardiopulmonary manifestations of SLE, including GC, conventional IS agents, biologics, targeted therapies, antifibrotic therapies, other adjuvant therapies, and interventional procedures. GC and conventional IS currently remain the most evidence-based approaches for managing most cardiopulmonary manifestations in SLE. Despite the availability of aggressive immunosuppressive therapies, patients with some conditions, such as LM, ALP, and DAH, still have high mortality. Based on current evidence, we have developed a series of possible treatment algorithms for each cardiopulmonary manifestation. However, there were some study limitations, including the limited quality of evidence due to the paucity of RCT data. This review highlights the need to optimize the management of cardiopulmonary manifestations in SLE patients to improve their prognosis, and the need for well-designed RCTs targeting particular SLE cardiopulmonary conditions.

## Supplementary Information

Below is the link to the electronic supplementary material.

Supplementary Material 1

## Data Availability

All data generated or analyzed during this study are included in this published article. Additional data is available from the corresponding author on reasonable request.

## Abbreviations

ACEI: Angiotensin-converting enzyme inhibitors
ACR: American College of Rheumatology
AE: Adverse event
ALP: Acute lupus pneumonitis
ANR: Anakinra
ARB: Angiotensin receptor blocker
AZA: Azathioprine
BEL: Belimumab
BNP: Brain natriuretic peptide
CAD: Coronary artery disease
CCB: Calcium channel blockers
CI: Cardiac index
CO: Cardiac output
CsA: Cyclosporin
CTD: Connective tissue disease
CYC: Cyclophosphamide
CVVH: Continuous veno-venous haemofiltration
DAH: Diffuse alveolar haemorrhage
DLCO: Diffusing capacity of the lung for carbon monoxide
ECMO: Extracorporeal membrane oxygenation
ED: Emergency department
ERA: Endothelin receptor antagonist
ERS: European Respiratory Society
ESC: European Society of Cardiology
eSPAP: Estimated systolic pulmonary arterial pressure
EQ-5D: EuroQol five dimensions questionnaire
EULAR: European League Against Rheumatism
FC: Functional class
FVC: Forced vital capacity
GC: Glucocorticoids
HCQ: Hydroxychloroquine
HR: Hazard ratio
IABP: Intra-aortic balloon pump counter pulsation
ICU: Intensive care unit
IIM: Idiopathic inflammatory myopathies
IL-6: Interleukin-6
ILD: Interstitial lung disease
IQR: Interquartile range
IS: Immunosuppressant
IV: Intravenous
IVIG: Intravenous immunoglobulin
LEF: Leflunomide
LM: Lupus myocarditis
LVEF: Left ventricular ejection fraction
MCTD: Mixed connective tissue disease
methyl-PNL: Methylprednisolone
MMF: Mycophenolate
mPAP: Mean pulmonary arterial pressure
MTX: Methotrexate
NSAIDs: Non-steroids anti-inflammatory drugs
NO: Nitric oxide
NT-proBNP: N-terminal pro brain natriuretic peptide
NYHA: New York Heart Association
PAH: Pulmonary arterial hypertension
PAP: Pulmonary arterial pressure
PBC: Primary biliary cirrhosis
PCA: Prostacyclin analogues
PDE-5: Phosphodiesterase 5
PE: Plasma exchange
PFT: Pulmonary function testing
PH: Pulmonary hypertension
PM/DM: Polymyositis/dermatomyositis
PNL: Prednisolone
PRA: Prostacyclin receptor agonist
pSS: Primary SjÖgren’s syndrome
PVR: Pulmonary vascular resistance
RA: Rheumatoid arthritis
RCT: Randomized clinical trial
RHC: Right heart catheter
RTX: Rituximab
RVSP: Right ventricular systolic pressure
SAE: Rerious adverse events
SF-36: Rhort form (36) health survey
sGCs: Soluble guanylate cyclase stimulator
SLE: Systemic lupus erythematosus
SLEDAI: Systemic Lupus Erythematosus Disease Activity Index
SLICC: Systemic Lupus International Collaborating Clinics classification
SLR: Systematic literature reviews
SLS: Shrinking lung syndrome
SSc: Systemic sclerosis
Tac: Tacrolimus
TCZ: Tocilizumab
UIP: Usual interstitial pneumonitis
UK: United Kingdom
WHO: World Health Organization
6MWD: 6-minute walk distance
95% CI: 95% confidence interval
