# Supplementary material for: Management of inflammatory cardiopulmonary manifestations in systemic lupus erythematosus: a systematic review
Source: Adv Rheumatol. 2026 May 29;66:47. doi: 10.1186/s42358-026-00551-1 (PMC13488853; doi:10.1186/s42358-026-00551-1)
Supplement: Supplementary file 1 [file 42358_2026_551_MOESM1_ESM.docx]

**Supplementary File 1. Full Search Strategy**

1. (lupus or systemic lupus erythematosus or SLE).mp.[mp=title, abstract, original title, name of substance word, subject heading word, floating sub-heading word, keyword heading word, organism supplementary concept word, protocol supplementary concept word, rare disease supplementary concept word, unique identifier, synonyms]

2. (pericardial effusion or pericarditis or myocarditis or arrhythmias or endocarditis or heart valve disease or cardiac conduction system disease or cardiac output or tamponade or cardiomegaly or cardiomyopathies or heart aneurysm or heart arrest or heart failure or pleuritis or pneumonitis or interstitial lung disease or pulmonary hypertension or pulmonary arterial hypertension or shrinking lung syndrome or alveolar hemorrhage or lung hemorrhage or pulmonary hemorrhage or cardiovascular or pulmonary or respiratory or lung or cardiopulmonary or cardiorespiratory).mp. [mp=title, abstract, original title, name of substance word, subject heading word, floating sub-heading word, keyword heading word, organism supplementary concept word, protocol supplementary concept word, rare disease supplementary concept word, unique identifier, synonyms]

3. (treatment OR therapy OR medication OR drug OR management)

4. (prednisolone or prednisone or steroids or corticosteroid or methylprednisolone or cyclophosphamide or methotrexate or leflunomide or azathioprine or mycophenolate mofetil or mycophenolic acid or tacrolimus or cyclosporin or non-steroidal anti-inflammatories or NSAIDS or hydroxychloroquine or colchicine or intravenous immunoglobulin or IVIG or antibiotics or rituximab or belimumab or anifrolumab or tocilizumab or anakinra or biologics or tofacitinib or baricitinib or Upadacitinib or jak inhibitors or plasma exchange or endothelin-1 antagonists or ambrisentan or bosentan or macitentan or tadalafil or sildenafil or phosphodiesterase type 5 inhibitor or riociguat or iloprost or Treprostinil or epoprostenol or selexipag or prostacyclin agonists) or nintedanib or pirfenidone or antifibrotic, .mp. [mp=title, abstract, original title, name of substance word, subject heading word, floating sub-heading word, keyword heading word, organism supplementary concept word, protocol supplementary concept word, rare disease supplementary concept word, unique identifier, synonyms]

4. #1 AND #2

5. # 3 or #4

6. #4 and #5

6. limit 5 to (English language, humans, adult (≥ 18 years old), and yr="1990-June 2025")

Additional search for clinical trials:

Search strategy: Above search strategy AND (clinical trial or randomized clinical trial or controlled clinical trial)

**Supplementary Table 1. Risk of bias assessment for all included studies.**

| **First author, published year (Trial/Study name)** | **Comparator** | **Sequence generation** | **Allocation concealment** | **Blinding of participants and personnel** | **Blinding of outcome assessment** | **Incomplete outcome data** | **Selective reporting** | **Overall** |
| --- | --- | --- | --- | --- | --- | --- | --- | --- |
| **Kahl et al, 1992^†^** | N/A | N/A | N/A | N/A | N/A | N/A | N/A | High |
| **Man et al, 2005^†^** | N/A | N/A | N/A | N/A | N/A | N/A | N/A | High |
| **Sherer et al, 2008^†^** | N/A | N/A | N/A | N/A | N/A | N/A | N/A | High |
| **Ngandeu et al, 2009^†^** | N/A | N/A | N/A | N/A | N/A | N/A | N/A | High |
| **Buppajamrntham et al, 2014^†^** | N/A | N/A | N/A | N/A | N/A | N/A | N/A | High |
| **Goswami et al, 2018^†^** | N/A | N/A | N/A | N/A | N/A | N/A | N/A | High |
| **Weich et al, 2005^†^** | N/A | N/A | N/A | N/A | N/A | N/A | N/A | High |
| **Morel et al, 2015^†^** | N/A | N/A | N/A | N/A | N/A | N/A | N/A | High |
| **Fava, et al, 2024^†^** | N/A | N/A | N/A | N/A | N/A | N/A | N/A | High |
| **Thomas et al, 2017^†^** | N/A | N/A | N/A | N/A | N/A | N/A | N/A | High |
| **DuToit et al, 2017^†^** | N/A | N/A | N/A | N/A | N/A | N/A | N/A | High |
| **Tanwani et al, 2018^†^** | N/A | N/A | N/A | N/A | N/A | N/A | N/A | High |
| **Wang et al, 2018^†^** | N/A | N/A | N/A | N/A | N/A | N/A | N/A | High |
| **Liu et al, 2022^†^** | N/A | N/A | N/A | N/A | N/A | N/A | N/A | High |
| **Law et al, 2005^†^** | N/A | N/A | N/A | N/A | N/A | N/A | N/A | High |
| **Chung et al, 2008^†^** | N/A | N/A | N/A | N/A | N/A | N/A | N/A | High |
| **van der Laan-Baalbergen et al, 2009^†^** | N/A | N/A | N/A | N/A | N/A | N/A | N/A | High |
| **Zawadowski et al, 2012^†^** | N/A | N/A | N/A | N/A | N/A | N/A | N/A | High |
| **Oudiz et al, 2004^§^** | Low | Low | Low | Low | Low | Low | Low | - |
| **Gonzalez-Lopez et al, 2004^§^** | Low | Low | High | High | Low | Low | Low | - |
| **Denton et al, 2006 (study 351 and BREATHE-1)^*^** | Low | Low | Low | Low | Unsure | Low | Low | - |
| **Badesch et al, 2007 (SUPER-1)^*^** | Low | Low | Low | Low | Unsure | Low | Low | - |
| **Girgis et al, 2007 (STRIDE-1 and STRIDE-1X)^*^** | Low | Low | Low | Low | Unsure | Low | Low | - |
| **Coghlan et al, 2017 (AMBITION)^*^** | Low | Low | Low | Low | Low | Low | Low | - |
| **Gaine et al, 2017 (GRIPHON)^*^** | Low | Low | Low | Low | Low | Low | Low | - |
| **Humbert et al, 2017 (PATENT-1 and 2)^*^** | Low | Low | Low | Low | Unsure | Low | Low | - |
| **Chen et al, 2015^#^** | Low | N/A | N/A | N/A | Unsure | Unsure | Unsure | - |
| **Cozzi et al, 2006^†^** | N/A | N/A | N/A | N/A | N/A | N/A | N/A | High |
| **Tanaka et al, 2002^†^** | N/A | N/A | N/A | N/A | N/A | N/A | N/A | High |
| **Sanchez et al, 2006^†^** | N/A | N/A | N/A | N/A | N/A | N/A | N/A | High |
| **Heresi et al, 2007^†^** | N/A | N/A | N/A | N/A | N/A | N/A | N/A | High |
| **Jais et al, 2008^¶^** | Low | High | High | High | Unsure | Unsure | Unsure | - |
| **Miyamichi-Yamamoto et al, 2011^¶^** | Low | N/A | N/A | N/A | Unsure | Unsure | Unsure | - |
| **Shirai et al, 2013^¶^** | Low | N/A | N/A | N/A | Unsure | Unsure | Unsure | - |
| **Kommireddy et al, 2015^†^** | N/A | N/A | N/A | N/A | N/A | N/A | N/A | High |
| **Yasuoka et al, 2018^†^** | N/A | N/A | N/A | N/A | N/A | N/A | N/A | High |
| **Qian et al, 2019^†^** | N/A | N/A | N/A | N/A | N/A | N/A | N/A | High |
| **Distler et al, 2024^†^** | N/A | N/A | N/A | N/A | N/A | N/A | N/A | High |
| **Li et al, 2024^†^** | N/A | N/A | N/A | N/A | N/A | N/A | N/A | High |
| **Luppino-Assad et al, 2025^†^** | N/A | N/A | N/A | N/A | N/A | N/A | N/A | High |
| **Robbins et al, 2000^†^** | N/A | N/A | N/A | N/A | N/A | N/A | N/A | High |
| **Palavutitotai et al, 2014^†^** | N/A | N/A | N/A | N/A | N/A | N/A | N/A | High |
| **Wan et al, 2016^†^** | N/A | N/A | N/A | N/A | N/A | N/A | N/A | High |
| **Barile et al, 1997^†^** | N/A | N/A | N/A | N/A | N/A | N/A | N/A | High |
| **Badsha et al, 2004^†^** | N/A | N/A | N/A | N/A | N/A | N/A | N/A | High |
| **Kazzaz, et al. 2015^†^** | N/A | N/A | N/A | N/A | N/A | N/A | N/A | High |
| **Andrade et al, 2016^†^** | N/A | N/A | N/A | N/A | N/A | N/A | N/A | High |
| **Wang et al, 2018^†^** | N/A | N/A | N/A | N/A | N/A | N/A | N/A | High |
| **Sun et al, 2020^†^** | N/A | N/A | N/A | N/A | N/A | N/A | N/A | High |
| **Li et al, 2023^†^** | N/A | N/A | N/A | N/A | N/A | N/A | N/A | High |
| **Garcia-Villanueva, et al. 2024^†^** | N/A | N/A | N/A | N/A | N/A | N/A | N/A | High |
| **Law, et al. 2023^†^** | N/A | N/A | N/A | N/A | N/A | N/A | N/A | High |
| **Zamora et al, 1997^†^** | N/A | N/A | N/A | N/A | N/A | N/A | N/A | High |
| **Santos-Ocampo et al, 2000^†^** | N/A | N/A | N/A | N/A | N/A | N/A | N/A | High |
| **Canas et al, 2007^†^** | N/A | N/A | N/A | N/A | N/A | N/A | N/A | High |
| **Wang et al, 2022^Δ^** | Low | High | High | High | Unsure | Unsure | Unsure | - |
| **Fischer et al, 2013^†^** | N/A | N/A | N/A | N/A | N/A | N/A | N/A | High |
| **Oldham et al, 2016^¶^** | Low | N/A | N/A | N/A | Unsure | Unsure | Unsure | - |
| **Wang et al, 2023^¶^** | Low | N/A | N/A | N/A | Unsure | Unsure | Unsure | - |
| **Feng, et al. 2024^¶^** | Low | N/A | N/A | N/A | Unsure | Unsure | Unsure | - |
| **Liu, et al. 2024** | N/A | N/A | N/A | N/A | N/A | N/A | N/A | High |
| **Deeb et al, 2018^†^** | N/A | N/A | N/A | N/A | N/A | N/A | N/A | High |
| **Karim et al, 2002^†^** | N/A | N/A | N/A | N/A | N/A | N/A | N/A | High |
| **Oud et al, 2005^†^** | N/A | N/A | N/A | N/A | N/A | N/A | N/A | High |
| **Borrell et al, 2016^†^** | N/A | N/A | N/A | N/A | N/A | N/A | N/A | High |
| **Duron et al, 2016^†^** | N/A | N/A | N/A | N/A | N/A | N/A | N/A | High |
| **Wallace et al, 2013**^§^ | Low | Low | Low | Low | Low | Low | Low | - |

† Studies without comparison were deemed at high risk of bias overall.

§ RCTs

* Post-hoc study or subgroup analysis from RCTs, the risk of bias assessments for these studies were based on their original RCTs.

\# Non-randomized trial. The comparator was from a historical group of patients.

¶ Retrospective cohort study with comparator. The comparator was from historical data or cohort.

Δ Prospective controlled cohort study.

Abbreviations: N/A: not applicable

**Supplementary Table 2 Pericarditis**

| **Study** | **Population** | **Tampon-ade** | **Intervention** | | | **Response** | **Recurrence within follow-up duration** |
| --- | --- | --- | --- | --- | --- | --- | --- |
|  |  |  | **Immunosuppressive therapy** | **Other therapy** | **Maintenance** |  |  |
| **Observational studies** | | | | | | | |
| Kahl et al., 1992  USA [19] | N=10  Age: range 18-68.6  F: 80% | 10 (100%) | Prednisone ≥60mg/d  Regimens: GC alone (10, 100%) | Pericardiocentesis: 5 (50%);  Pericardial window: 4 (40%) | ND | Resolved: 6 (60%); recurrent: 2 (20%); Pericardial thickening: 2 (20%); Death: 1 (10%) | 2 (20%) patients had recurrence at 6 and 2 months, respectively. Treatment for recurrence: Prednisone dose increased (Pulse mmethyl-PNL for one patient). |
| Man et al., 2005  HongKong [20] | N=37 in total with serositis including 18 with pericarditis  Age: 31.8±13.3  F: 89% | 5/18 (27.8%) | i) NSAIDs: 13/37 (35%) including 2 with NSAIDs alone;  ii) PNL (mean dose: 33.6±15 mg/d): 35/37 (94.6%);  iii) Oral CYC (for tamponade): 2/37 (5.4%) for 3 months.  Regimens: NSAIDs alone (2, 5.4%), GC alone (33, 89.2%), GC+IS (2, 5.4%) | ND | ND | Resolved by NSAIDs alone: 2/2 (100%); Resolved by PNL or PNL combined other therapy: 35/35 (100%) | 9 patients (24%) had recurrence including one patient with pericarditis within 46±40 months. Treatment for recurrence: NSAIDs or PNL (small to moderate doses) with 100% response. No restrictive pericarditis or pericardial fibrosis. |
| Sherer et al., 2008  Israel [21] | N=62 SLE patients in total including patients with pericarditis (number ND)  Age: 40±15  F: 88.7% | no | i) IVIG as add-on therapy: 62 (100%) (approximal 0.5 g/kg/each course for mean 6±6 courses with 5±2 weeks of interval between courses);  ii) Other immunosuppressive treatment: ND | ND | ND | Response to IVIG in patients with pericarditis: 60% | ND |
| Ngandeu et al., 2009  Cameroon [22] | N=10  Age: 26 (range 13-65)  F: 100% | no | i) Prednisone (60 mg/d): 8 (80%);  ii) Pulsed methyl-PNL (500 mg/d for 3 days) followed by oral prednisone (60 mg/d): 2 (20%);  iii) HCQ (400m/d): 10 (100%)  Regimens: GC+HCQ (10, 100%) | ND | ND | Resolved: 10 (100%) | 4 (40%) patients had recurrence at 3, 5, 6, 24 months, respectively. Treatment for recurrence: prednisone dose increased with 100% rapid response. |
| Buppajamrntham et al., 2014  Thailand [23] | N=81 (82 episodes including 76 episodes due to active SLE, 4 episodes due to suspected TB, and 2 episodes with inconclusive diagnosis)  Age: 29.3±11  F: 92% | 13 (16.0%) | Treatment for lupus pericarditis (n=76):  i) NSAIDs: 2/76 episodes (2.6%);  ii) GC: 74/76 episodes (97.4%) (dose ND)  Regimens for lupus pericarditis: NSAIDs alone (2, 2.6%), GC alone (74, 97.4%)  Treatment for TB pericarditis (n=4): anti-TB agents: 4/4 (100%) | ND | ND | Outcomes of patients with lupus pericarditis:  i) good response: 61/76 (80%);  ii) inconclusive: 12/76 (16%);  iii) unknown: 3/76 (4%)  Outcomes of patients with TB pericarditis:  good response: 4/4 (100%) | ND |
| Goswami et al., 2018  India [24] | N=24  Age: 27.6±9.5  F: 94.9% | 24 (100%) | i) Oral PNL (1 mg/kg/d): 12 (50%);  ii) Pulsed methyl-PNL (500 or 1000mg/d): 12 (50%);  iii) IV CYC monthly (750–1000mg/dose): 20 (83.3%)  Regimens:  Group 1-oral GC alone (4, 16.7%); Group 2-oral GC+ CYC (8, 33.3%); Group 3-pulsed GC followed by oral GC+ CYC: 12 (50%). One patient in Group 3 was further treated with RTX due to worsening clinical condition. | Pericardiocentesis: 24 (100%);  Surgical intervention: 1 (4.2%) | Reduced PNL (mean 9.7±3.5 mg/d at 6 months) | Response: 23/24 (95.8%);  Mortality: 2/24 (8.3%) with cause not reported. | Recurrence was not reported with median 18 (interquartile range 12–24) months of follow-up. Three patients were lost to follow-up within the first year. |
| **Case series and case reports** | | | | | | | |
| Weich et al., 2005  South Africa [25] | N=8  Age: 29.5±10.7  F: 87.5% | 7 (87.5%) | i) Pulsed methyl-PNL for tamponade followed by prednisone: 2 (25%);  ii) Prednisone (30mg-60/day): 6 (75%);  iii) CYC: 1 (12.5%) (complicated with lupus nephritis)  Regimens: GC alone (7, 87.5%), GC+IS (1, 12.5%) | Pericardiocentesis: 8 (100%) | ND | Good response: 6 (75%);  Mortality: 2 (25%) including one died at five days without describing cause and one died of probable pneumonia at one month, respectively | no recurrence happened within follow-up duration for the 5 survivors. |
| Morel et al., 2015  France [26] | N=10  Age: 35±12  F: 90% | no | i) Colchicine (1mg/d) added on previous treatment (prednisone (7, 2-30mg/d), HCQ (7), AZA (3), MTX (2), MMF (1), and low-dose aspirin): 10 (100%);  ii) Corticosteroids dose increased (9mg/d and 15mg/d, respectively): 2 (20%)  Regimens: colchicine +immunosuppressive drugs (10, 100%) | ND | Colchicine continuation: 6 (60%) for a median 12.3 months (range: 15 days-4.5 years) | Resolved: 10 (100%) | no recurrence during colchicine treatment;  2 (20%) patients had recurrence after Colchicine discontinuation. Treatment for recurrence: Colchicine resumed with 100% response. |
| Fava, et al, 2024. USA [27] | N=4  Age: : range 26-44  F: 100% | no | Previous ineffective treatments:  NSAIDs: 4 (100%)  Colchicine: 3 (75%)  Hydroxychloroquine: 4 (100%)  GC: 3 (75%)  IS: 4 (100%), including methotrexate, azathioprine, mycophenolate  Belimumab: 2 (50%)  Rituximab: 2 (50%)  Anakinra: 2 (50%)  Switched to  Rilonacept (160mg weekly): 4 (100%) | ND | ND | Resolved: 3 (75%);  Significantly improved: 1 (25%) | Recurrence was mentioned in one patient (25%) after stopping rilonacept. |

**Abbreviation:** AZA: azathioprine; CYC: cyclophosphamide; F: female; HCQ: hydroxychloroquine; IV: intravenous; IVIG: intravenous immunoglobulin; methyl-PNL: methylprednisolone; MMF: mycophenolate; MTX: methotrexate; ND: not described; NSAIDs: non-steroids anti-inflammatory drugs; PNL: prednisolone.

**Supplementary Table 3.** **Myocarditis**

| **Study ID** | **Population** | **Intervention** | | | **Response** | **Recurrence** |
| --- | --- | --- | --- | --- | --- | --- |
|  |  | **Immunosuppressive therapy** | **Other therapy** | **Maintenance** |  |  |
| **Observational studies** | | | | | | |
| Thomas et al., 2017  France [28] | N=29  Age: 30 (range 16-57)  F: 89.6% | i) Pulsed methyl-PNL (500-1000mg/day for 1-3 days): 20 (69%) followed by oral prednisone (0.5-1mg/kg/day);  ii) Prednisone (0.5-1mg/kg/day): 9 (31.0%);  iii) IVIG: 8 (27.6%) including 7 with single course 2g/kg in 3-5 days and 1 with repeated infusions monthly for 6 months;  iv) CYC (500-1300mg IV monthly for 2-10 months): 16 (55.2%);  v) MMF: 2 (6.9%);  vi) PE: 4 (13.8%) including one with PE alone and the other three in combination with other therapy.  Regimens: oral GC alone (9, 31.0%); pulse GC+IS±IVIG (18, 62.1%); pulse GC+ long course IVIG (1, 3.4%); PE alone (1, 3.4%) | ICU admission: 25 (86%);  Vasopressors and/or inotropic support (epinephrine, norepinephrine, and/or dobutamine): 11 (37.9%);  CVVH: 4 (13.8%);  ECMO: 3 (10.3%) | Prednisone (<=10mg/day): 17/25 (68%);  Prednisone (>10mg/day): 2/25 (8%);  MMF: 10/25 (40%);  HCQ: 22/25 (88%) | Proportion of patients with LVEF ≥ 55% (primary outcome) improved from 6/29 (21%) at baseline 10/23 (43%) at 1 month;  LVEF ≥ 55% at the end of follow-up (median 37 months, range 4-115 months): 21/26 (81%);  Final LVEF improved compared with the baseline (p < 0.0001);  Troponin levels normalised at the end of follow-up: 26/26 (100%);  Mortality during initial treatment: 3/29 (10.3%), including one with PE alone therapy who died before initiating GC.  Mortality during long-term follow-up: 1/26 (3.8%). This patient died at 4 years from an unknown cause. | One patient relapsed at 2 months after the end of CYC treatment without maintenance therapy, and improved again with pulsed methyl-PNL |
| DuToit et al., 2017  South Africa [29] | N=28  Age: 28.3±11.3  F: 92.9% | i) Pulsed methyl-PNL (500-1000mg/day for 3 days): 19 (67.9%) including 4 receiving repeated pulse for resistant or relapsing LM;  ii) Prednisone (1mg/kg/day): 27 (96%);  iii) CYC: 21 (75%)±  additional IS (AZA, IVIG, and RTX): 4 (14.3%);  iv) Combination of prednisone, MTX and PE for concomitant thrombotic thrombocytopenic  purpura: 2 (7.1%)  Regimens: GC alone (5, 17.9%); GC+IS (21, 75%), GC+IS+PE (2, 7.1%) | ICU admission with ventilatory support: 19 (67.9%);  Anti-failure therapy included ACEI (71.4%), diuretics (75%), beta-blockers (60.7%), and inotropes (14.3%). | AZA: 14 (50%) or MMF: 1 (3.6%) | Final LVEF improved to ≥40% within median 563 days (range 4-1740 days) of follow-up: 14 (50%);  Mortality: 12 (42.9%) at a median of 115 days (5 died of LM. One died of multisystem involvement; five died of treatment related complications)  AE: 13 (46.4%) including  bone marrow suppression (25%), septicaemia  (32%) and opportunistic infections (25%). | 2 (7.1%) patients had recurrence at 568 and 135 days, respectively. The second patient relapsed again at 447 days. |
| Tanwani et al., 2018  Canada [30] | N=30  Age: 34.8±12.9  F: 83.3% | i) Pulsed methyl-PNL (500mg/d–1000mg/day for 3 days): 3 (10%);  ii) Oral prednisone (median 35mg/d): 30 (100%)  iii) IS: 21 (70%) including AZA  (12, 40%), MMF (6, 20%) and  CYC (3, 10%).  iv) Antimalarial: 18 (60%)  Regimens: GC alone (9, 30%); GC+IS (21, 70%) | Anti-failure therapy included diuretics: 15 (50%); ACEI or ARB: 16 (53.3%);  beta-blockers: 17 (56.7%); calcium-channel blockers: 7 (23.3%)  Antiplatelet/  anticoagulant agents: 18 (60%) | ND | Outcomes within a median 16±8.1 months of follow-up:  Completely resolved: 15 (50%)  Partially resolved: 8 (26.7%)  All-cause mortality: 7 (23.3%). Two died of cardiogenic shock at 3 and 17 months, respectively; one died of other lupus organ involvement; and four died of dilated cardiomyopathy and congestive heart failure at 5, 12, 18, and 19 years respectively. | ND |
| Wang et al., 2018  Taiwan [31] | N=13  Age: 31.9±10.7  F: 100% | Regimens:  i) High-dose GC alone: number ND;  ii) Pulsed methyl-PNL followed by high-dose GC, and/or IS (AZA, CYC, or MMF): number ND;  iii) RTX 375 mg/m2 fortnightly × 2 or weekly ×4 in addition to high-dose GC and IS: 3 (23.1%) including 1 high-dose oral GC+AZA+RTX, 1 pulse methyl-PNL+IV CYC+RTX, and 1 high-dose oral GC+MMF+RTX | no | ND | For the 3 patients receiving RTX therapy:  NYHA FC improved from III/IV at baseline to I: 3 (100%)  LVEF increased from 19.0% to 62.8% at 5 months for one patient, and from 38.5% to 59.6% at 5 months for another patient;  The cardiac MRI repeated for one patient: improved LVEF and delayed gadolinium enhancement on left ventricle. | ND |
| Liu et al, 2022 China [36] | N=79  Age: 32.4±13.6  F: 89% | i) Pulsed methyl-PNL (500mg/d–1000mg/day for 3 days): 52 (91.2%);  ii) Oral prednisone: 22 (27.8%) (0.5-1.0 mg/kg/day); 3 (3.8%) (<0.5mg/kg/d)  iii) IS: 79 (100%), including CYC (58, 73.4%), MMF (16, 20.2%), Tac (8, 10.1%), and CsA (2, 2,5%).  iv) Antimalarial: 47 (59.5%)  v) IVIG: 28 (35.4%)  vi) PE: 7 (8.9%)  Regimen: GC+IS (51, 64.6%); GC+IS+IVIG/PE (28, 35.4%) | ND | ND | Improvement in LVEF before discharge: 14/24 (58%);  Improvement in LVEF after discharge: 18/22 (82%)  Mortality during hospitalization: 10 (13%) | ND |
| **Case series and case reports** | | | | | | |
| Law et al., 2005  Singapore [32] | N=11  Age: 27±10  F: 100% | i) IV High-dose GC: 9 (81.8%) including 4 (36.3%) from IV hydrocortisone switched to pulse methyl-PNL (500mg/day for 3 days);  ii) Oral PNL (1mg/kg/d): 10 (90.9%);  iii) IV CYC (500-700 mg/m2 monthly): 7 (63.6%) including 4 for 6 doses, 1 for 3 doses and 2 for 1 dose (patients receiving ≥3 courses of CYC had concomitant involvement of other major organs)  iv) Oral CYC followed by AZA: 1 (9.1%)  Regimens: GC alone (3, 27.3%); GC+IS (8, 72.7%) | ACEI, diuretic, and digoxin | low dose PNL (≤7.5mg/day), HCQ, AZA alone or combination: 8/9 (88.9%); off treatment 1/9 (9.1%) | LVEF improved to normal at 6 months or later: 8/9 (88.9%);  Mortality: 2 (18.2%) with long-term SLE duration died of infection | no recurrence for 9 patients within a median 4 (2.5-10.1) years |
| Chung et al., 2008  South Korea [33] | N=5  Age: range 23-63  F: 80% | i) Pulsed methyl-PNL (1000mg for 3 days): 2 (40%);  ii) Oral PNL (1mg/kg/d): 5 (100%);  iii) IV CYC (monthly for 12 doses): 1 (20%);  iv) IVIG (0.4g/kg/day for 5 days): 1 (20%)  Regimens: GC alone (4, 80%); GC+IVIG+IS (1, 20%) | mechanical ventilation, digoxin, diuretics, beta-blocker, and ACEI: 1 (20%) | tapered prednisone: 4 (80%);  CYC (IV monthly for 12 doses): 2 (40%) | LVEF improved: 4 (80%) (from 22-40% at baseline to 54-72% at the last follow-up);  Mortality: 1 (20%) died of LM | no recurrence for 4 patients within up to 33 months of follow-up |
| van der Laan-Baalbergen et al., 2009  UK [34] | N=6  Age: range 19-40  F: 100% | i) Pulsed methyl-PNL (1000mg for 3 days): 6 (100%) (including one patient with two courses and one patient switched from oral prednisone 60mg to pulsed methyl-PNL due to no improvement) followed by oral prednisone;  ii) AZA: 3 (50%) (2 of them were switched to CYC due to no improvement);  iii) CYC (750mg/m^2^ monthly for 7-12 doses): 5 (83.3%) including two patients switched from AZA;  iv) IVIG (36g/day for 4 days/course for 3 courses): 1 (16.7%)  Regimens: GC+IS (5, 83.3%); GC+IS+IVIG (1, 16.7%) | diuretics, ACEI, oxygen, inotropic when required;  IABP: 1 (16.7%); CVVH: 1 (16.7%) | ND | LVEF improved within 77 months (range 43 to 113 months) of follow-up: 6 (100%) (increased from 23%-37% at baseline to 42%-60%);  no improvement to oral prednisone: 1 (16.7%);  no improvement to AZA: 2 (33.4%) | no recurrence within 4-10 years |
| Zawadowski et al., 2012  USA [35] | N=24  Age: 47.6±20.4  F: 79% | i) Pulsed methyl-PNL (500-1000mg for 1 to 5 days): 9 (37.5%) followed by dose tapering;  ii) IV or oral methyl-PNL (40-80mg/day or equivalent for 5 to 10 days): 12 (50%) followed by dose tapering;  iii) MMF (500mg twice daily): 6 (25%);  iv) CYC (300-600 mg/m^2^ monthly for 3 to 6 months): 3 (12.5%);  v) AZA (100mg/day): 3 (12.5%);  vi) RTX (every 2 weeks for 4 weeks, dose ND): 1 (4.2%);  vii) IVIG (for 5 days): 1 (4.2%)  Regimens: ND | ICU admission: 8 (33%);  beta-blocker: 18 (75%);  ACEI or ARB: 17 (71%);  Diuretic: 14 (58%);  Digoxin: 7 (29%);  inotropic medication: 2 (8.3%) | ND | LVEF improved after a mean 7.2 months: 16 (67%) (from 33.8% at baseline to 49.5%);  Mortality: 3 (12.5%) (1 died of LM during hospitalisation, 1 died of heart failure one year after hospitalisation; and 1 died of withdrawal of dialysis one year after hospitalisation) | ND |

**Abbreviation:** ACEI: angiotensin-converting enzyme inhibitors; ARB: angiotensin receptor blocker; AZA: azathioprine; CsA: cyclosporin; CYC: cyclophosphamide; CVVH: continuous veno venous haemofiltration; ECMO: extracorporeal membrane oxygenation; F: female; FC: functional class; HCQ: hydroxychloroquine; IABP: intra-aortic balloon pump counter pulsation; ICU: intensive care unit; IV: intravenous; IS: immunosuppressant; IVIG: intravenous immunoglobulin; LM: lupus myocarditis; LVEF: left ventricular ejection fraction; methyl-PNL: methylprednisolone; MMF: mycophenolate; MTX: methotrexate; ND: not described; NSAIDs: non-steroids anti-inflammatory drugs; NYHA: New York Heart Association; PE: plasma exchange; PNL: prednisolone; RTX: rituximab; Tac: Tacrolimus.

**Supplementary Table 4.** **Pulmonary hypertension/pulmonary arterial hypertension study design and treatment^#^**

| **Study ID** | **Population** | **Comparator**  **(patient number in each group)** | **Intervention** | | | | **Endpoints** |
| --- | --- | --- | --- | --- | --- | --- | --- |
|  |  |  | **Immunosuppressive therapy** | **PAH-targeted therapy** | **Other therapy** | **Maintenance** |  |
| **RCTs or Post-hoc study/ subgroup analysis of RCTs** | | | | | | | |
| Oudiz et al., 2004  Multinational [37] | N=470 in total (CTD-PAH: 90 including 45 SSc, 25 SLE, and 20 MCTD/overlap  Age (CTD-PAH patients): 54±2 vs 48±2  F (CTD-PAH patients): 93% vs 88% | Treprostinil vs placebo  ---CTD-PAH patients: 41 vs 49;  ---SLE-PAH patients: 7 vs 18 | ND | Treprostinil (Initiated from 1.25 to 2.5 ng/kg/min. and doses up-titrated in increments of 1.25 to 2.5 ng/kg/min every 1 to 2 weeks up to 22.5 ng/kg/min) vs placebo | PAH conventional therapy (ie, anticoagulants,  oral vasodilators, diuretic agents, cardiac glycosides, and  supplemental oxygen) | ND | Changes in 6MWD, composite score of  signs and symptoms of PAH, dyspnea-fatigue rating, Borg dyspnea  score, quality-of-life scores, and hemodynamic variables from baseline to 12 weeks |
| Gonzalez-Lopez et al., 2004  Mexico [38] | N=34 (SLE-PH)  Age: 36±10 vs 41±12)  F: 100% vs 100% | IV CYC vs oral enalapril (16 vs 18) | CYC (IV 500mg/m2 monthly) vs oral Enalapril (10mg/day) for 6 months  GC (≤15mg/d) and antimalarial were allowed | No | ND | ND | Primary: reduction of SPAP from baseline to 6 months # |
| Denton et al., 2006 (Study 351 & BREATH)  Multinational [39] | N=66 in the RCTs including 52 SSc, 8 SLE, 4 overlap, and 2 unclassified CTD; 64 of them entered the extension trial  Age (CTD-PAH patients): 57.7±12.6 vs 49.7±12.7  F (CTD-PAH patients): 86.4% vs 77.3% | Bosentan vs placebo ---CTD-PAH patients: 44 vs 22;  ---SSc-PAH patients: 37 vs 15  ---SLE-PAH patients: 5 vs 3 | ND | Bosentan (62.5 mg  twice daily for 4 weeks, and then to  125 or 250 mg twice daily) vs placebo | ND | Bosentan for all patients in the open-label extension trial including 40 remained on Bosentan  monotherapy, 1 received Prostanoids in addition to Bosentan  and 19 discontinued during follow-up | RCTs: 6MWD, WHO FC and time to clinical worsening at 12 or 16 weeks$;  Extension trial:  status at the last visit (alive/dead, without documentation  of reason for death), lung transplantation and initiation of prostanoid |
| Badesch et al., 2007 (SUPER-1)  Multinational [40] | N=278 in total (CTD-PAH: 84 including 38 SSc, 19 SLE, 12 CREST syndrome, 8 MCTD, 4 pSS, 1 Still’s disease, 1 PBC, and 1 RA)  Age (CTD-PAH patients): 53±15  F (CTD-PAH patients): 83.3% | Sildenafil (20mg, 40mg, or 80mg group) vs placebo  ---CTD-PAH patients: 62 (21, 20, 21 at the 20mg, 40mg, and 80mg group, respectively) vs 22 | ND | Sildenafil (20mg, 40mg, or 80mg three times daily) vs placebo | PAH background therapy (e.g., anticoagulants, digoxin, calcium channel  blockers, diuretics, and/or supplemental oxygen) | ND | Primary: change in 6MWD from baseline to 12 weeks;  Secondary: change in WHO FC and hemodynamic  Measures from baseline to 12 weeks |
| Girgis et al., 2007 (STRIDE-1 and STRIDE-1X)  USA & Canada [41] | N=178 in total (CTD-PAH: 42 including 19 SSc, 16 SLE, 7 MCTD or overlap syndrome; 42 of them entered the extension trial)  Age (CTD-PAH patients): 50±11 vs 43±18  F: 94% vs 89% | Sitaxsentan (100mg or 300mg group) vs placebo  ---CTD-PAH patients: 33 (16 and 17 at 100mg and 300mg group, respectively) vs 9 | ND | Sitaxsentan (100mg or 300mg) vs placebo | Warfarin, diuretics, calcium channel blockers, digoxin | Sitaxsentan (100mg or 300mg) for all patients in the open-label extension trial | Changes in 6MWD and other continuous  variables (SF-36 and haemodynamics) from baseline to Week 12 |
| Coghlan et al., 2017 (AMBITION)  Multinational [42] | N=500 in total (CTD-PAH: 187 including 118 SSc, 23 MCTD, 17 SLE, 3 overlap, and 26 other CTDs)  Age (CTD-PAH patients): 58.4±12.3 vs 57.9±13.1  F (CTD-PAH patients): 86% vs 90% | Combination therapy (Ambrisentan plus  Tadalafil) vs Monotherapy (Ambrisentan or Tadalafil)  ---CTD-PAH patients: 103 vs 84;  ---SLE-PAH patients: 11 vs 6 | GC: 18/103 (17%) vs 17/84 (20%);  IS: 14/103 (14%) vs 13/84 (15%); | Combination therapy: Ambrisentan 10mg/day plus  Tadalafil 40 mg/day vs monotherapy (Ambrisentan 10 mg/day or Tadalafil 40 mg/day ) | ND | ND | Primary: the time from randomisation to first  adjudicated clinical failure¶;  Secondary: change from baseline at week  24 in NT-proBNP, satisfactory clinical response to therapy, 6MWD, Borg dyspnoea index and WHO FC‡. |
| Gaine et al., 2017 (GRIPHON)  Multinational [43] | N=1156 in total (CTD-PAH: 334 including 170 SSc, 82 SLE, 82 MCTD/other CTD)  Age (CTD-PAH patients): 51.8±14.1 vs 52.8± 15  F (CTD-PAH patients): 92.8% vs 87.4% | Selexipag vs placebo  ---CTD-PAH patients: 167 vs 167;  ---SLE-PAH patients: 45 vs 37 | Baseline treatment:  GC: 31/45 (68.9%) vs 26/37 (70.3%);  IS: 9/45 (20%) vs 10/37 (27%) | Baseline treatment: Calcium channel blockers: 4/45 (8.9%) vs 7/37 (18.9%); ERA: 12/45 (26.7%) vs 8/37 (21.6%); PDE-5i: 9/45 (20.0%) vs 13/37 (35.1%); ERA+PDE-5i: 9/45 (20.0%) vs 6/37 (16.2%); and  trial medication: Selexipag vs placebo | ND | ND | Primary: time to first morbidity/mortality event*;  Secondary: change in 6MWD from baseline to Week 26, and all-cause death |
| Humbert et al., 2017 (PATENT-1 and 2)  Multinational [44] | N=443 in total in PATENT-1 trial (CTD-PAH: 111 including 66 SSc, 18 SLE, 11 RA, 10 MCTD, and 6 unspecific CTD); 94 CTD-PAH patients completed PATENT-1 and entered PATENT-2 trial.  Age (CTD-PAH patients): 57±14  F (CTD-PAH patients): 88% | Riociguat (Maximum- 2.5 mg group)  vs placebo  ---CTD-PAH patients: 71 vs 25 | Immunomodulating agents: 27 (24.3%) including NSAIDs, AZA, LEF, MTX, mizoribine, MMF/MPA, Tac, and CYC | Ricociguat  (Maximum 2.5 mg  three times daily) vs placebo (CTD-PAH patients: 71 vs 25) | ND | All patients in PATENT-2: Riociguat adjusted up to a maximum of 2.5 mg tid  until all patients transition to commercially available Riociguat. | PATENT-1: Primary: change in 6MWD from baseline to end of Week 12;  Secondary: change in PVR, NT-proBNP, WHO FC, time to clinical worsening†, Borg dyspnoea score and quality of life scores.  PATENT-2: Safety, tolerability, and 6MWD and WHO FC change in long-term Ricociguat. |
| **Non-randomised trial** | | | | | | | |
| Chen et al., 2015  China [45] | N=22 (SLE-PH)  Age: 31.3±7.5 vs 37.5±2.0  F: 100% vs 90.9% | Proactive intense care^⁑^ vs historical control group (11 vs 11) | mean PNL dose 44.4±5.8 vs 166.1±1 20.8 (p=0.354) | Sildenafil: 4 (36.4%) vs 1 (9.1%);  Iloprost: 8 (72.7%) vs 0 (0.0%);  Bosentan: 8 (72.7%) vs 1 (9.1%);  Combination therapy: 8 (72.7%) vs 0 (0%).  Regimen of combination therapy: 4 Bosentan+Iloprost; 1 Bosentan+Sildenafil, and 3 Bosentan+Iloprost+Sildenafil. | ND | ND | survival rate in 3 months and WHO functional class change |
| **Prospective non-controlled study** | | | | | | | |
| Cozzi et al., 2006  Italy [46] | N=13 CTD-PH including 9 SSc, 2 SLE, and 1 MCTD    Age (CTD-PAH patients): range 34-75  F (CTD-PAH patients): 84.6% | no | ND | Bosentan: 13 (100%), mostly nonresponders to prostanoids therapy | ND | Bosentan for 1 year | Right ventricular systolic pressure, PAP, 6MWD |
| **Observational studies** | | | | | | | |
| Tanaka et al., 2002  Japan [47] | N=12 (SLE-PH)  Age: 38.3±14.2  F: 91.7% | no | i) Oral PNL (35-60mg/d): 8 (66.7%);  ii) pulsed methyl-PNL (500-1000 mg/day for 3 days) followed by oral PNL (40-60mg/d): 4 (33.3%);  iii) CYC (oral 50mg per day or every other day or IV 300mg for 4 doses): 3 (25%)  Regimens: oral GC alone (40-60mg/day) (5, 41.7%); pulsed GC followed by oral GC (4, 33.3%); oral GC (35-40mg/d) + CYC: 3 (25%) | calcium channel blocker and oral prostaglandin I^2^ for 8 (66.7%) patients with right heart failure | anticoagulants and/or diuretics for 8 (66.7%) patients with right heart failure | ND | Response to treatment for PH** |
| Sanchez et al., 2006  France [48] | N=28 CTD-PAH including 13 SLE  Age (CTD-PAH patients): 28.9±7.7  F (CTD-PAH patients): 100% | no | i) Pulsed methyl-PNL (500mg/day for 3 days) followed by oral prednisone (0.5-1.0 mg/kg/day): 5/28 (17.9%);  ii) oral prednisone (0.5-1.0 mg/kg/day): 17 (60.7%);  iii) CYC (IV 600 mg/m^2^ monthly for ≥3 doses): 28 (100%) including 13 SLE patients  Regimen: CYC alone (6, 21.4%); pulse GC followed by oral GC+CYC (5, 17.9%); oral GC+CYC (17, 60.7%) | no PAH-targeted therapy as first line therapy | anticoagulation, diuretics: 13/13 (100%) for SLE patients; 28/28 (100%) in total | ND | Responders to immunosuppressive therapy## |
| Heresi et al., 2007  USA [49] | N=12 (SLE-PAH)  Age: 43±10  F: 100% | no | Prednisone: 7 (58.3%);  HCQ: 8 (66.7%);  IS (MTX, CYC, or MMF): 4 (33.3%)  Regimens:  GC alone (2, 16.7%); HCQ alone (2, 16.7%);  GC + HCQ: 2 (16.7%); IS+HCQ: 1 (8.3%); GC ± HCQ +IS (MTX, CYC, or MMF): 3 (33.3%); None: 2 (16.7%) | Monotherapy therapy at start: 12 (100%) including epoprostenol (8,66.7%), bosentan (2, 16.7%), treprostinil (2, 16.7%)  After a mean  follow-up of 41±25 months, monotherapy: 7 (58.3%) including epoprostenol (2,16.7%), bosentan (4, 33.3%), and sildenafil (1, 8.3%);  combination therapy: 5 (41.7%) including epoprostenol+ bosentan (3, 25%), treprostinil+ bosentan (1, 8.3%), bosentan+sildenafil (1, 8.3%) | Warfarin: 9 (75%) | ND | 6MWD, WHO functional class, RVSP, mPAP |
| Jais et al., 2008  France [50] | N=23 CTD-PAH including 13 SLE, 10 MCTD  Age (CTD-PAH patients): 31±10 vs 38±9  F (CTD-PAH patients): 87.5% vs 85.7% | first-line immunosuppressive therapy alone vs immunosuppressive therapy combined with pulmonary vasodilators  ---CTD-PAH patients: 16 vs 7 | Prednisone (0.5-1 mg/kg/d for 4 weeks): 16 (100%) vs 7 (100%);  CYC (IV 600 mg/m2 monthly for 6 months): 16 (100%) vs 7 (100%). | PAH target therapy: 0 (0%) vs 7 (100%) including Bosentan (3, 42.9%), Epoprostenol (3, 42.9%), and SC Treprostinil (1, 14.2%) | anticoagulants, diuretics | Tapered prednisone: 16 (100%) vs 7 (100%);  MMF 2 (8.7%, group ND) | Responder and hemodynamic improvement§ |
| Miyamichi-Yamamoto et al., 2011  Japan [51] | N=21 CTD-PAH including 8 SLE, 4 MCTD, 4 pSS, 3 SSc, 1 RA, and 1 DM.  Age: 42±8  F: 92.3% | Intensive immunosuppressive therapy (IIT)  vs historical control ----CTD-PAH patients: 13 vs 8;  ---SLE-PAH patients: 7 vs 1 | IIT group:  Oral GC (1 mg/kg/day in the first month, followed  by tapering by 5-10 mg/day every 2-4 weeks  to a maintenance dose of 5-10 mg/day): 13 (100%);  CYC (500 mg IV for 10 times in a year once a month for 3 months followed by once every 1-3 months): 13 (100%)  Regimens of IIT:  oral GC+CYC (13, 100%)  Historical control group: no immunosuppressive therapy | Oral beraprost (12, 57.1%), or IV epoprostenol (5, 23.8%) during  hospitalization, bosentan (7, 33.3%), sildenafil, or tadalafil (6, 28.6%) as monotherapy or in combination for both groups | ND | IIT group: CYC, low dose glucocorticosteroids (5-10mg/day), and vasodilator therapy;  Historical control group: vasodilator therapy | Hemodynamic parameters and survival.  Hemodynamic responder was defined as mPAP reduction <25 mmHg or >10% reduction of baseline value. |
| Shirai et al., 2013  Japan [52] | N=32 CTD-PAH including 12 SLE, 10 MCTD, 8 SSc, and 2 pSS.  Epoprostenol group includes 6 SLE, 5 MCTD, 4 SSc, and 1 pSS patients    Age (CTD-PAH patients): 43±14 vs 36±18  F (CTD-PAH patients): 100% vs 100% | Epoprostenol group vs control group:  ---CTD-PAH: 16 vs 16;  ---SLE-PAH: 6 vs 6 | Immunosuppressive therapy in two groups was matched | Epoprostenol group:  Epoprostenol monotherapy 12 (75%), Epoprostenol combined with Sildenafil 2 (12.5%), Epoprostenol combined with Sildenafil and Bosentan 2 (12.5%)  Except for Epoprostenol, all the other PAH-targetd therapy were matched in two groups | ND | All 16 patients in Epoprostenol group continued Epoprostenol;  Sildenafil and Bosentan was added to Epoprostenol for 4 and 1 patients, respectively | Short-term efficacy of epoprostenol including changes of FC, BNP, and hemodynamic  parameters from baseline to 6 months;  Long-term survival of epoprostenol and control group in 52 ± 27 (range 9-90) months |
| Kommireddy et al., 2015  India [53] | N=24 (SLE-PH)  Age: 25.4±8.0  F: 100% | no | Oral GC (0.5-1 mg/kg for 4 weeks): 24 (100%);  IV CYC 500-1000 mg/m2 monthly (23, 95.8%) or fortnightly (1, 4.2%) for 6 doses: 24 (100%)  Regimen: GC+IS (24, 100%) | Sildenafill or Tadalafil: 20 (83.3%) | ND | Tapered steroids and Sildenafill/ Tadalafil | Responder was defined as a decrease in PASP of 15 mmHg from baseline along with improvement in NYHA functional class. |
| Yasuoka et al., 2018  Japan [54] | N=30 CTD-PAH including 13 SLE, 14 MCTD, and 3 pSS  Age (CTD-PAH patients): 34.7±12.9  F (CTD-PAH patients): 100% | no | Regimens:  i) PNL (60mg/day with rapid tapering protocol) alone: 20 (66.7%);  ii) PNL with CYC (IV 600g/m2 monthly for 6 doses) followed by AZA: 8 (26.7%);  iii) PNL with AZA: 1 (3.3%);  iv) PNL with Tac and MMF: 1 (3.3%)  In summary, GC alone (20, 66.7%), GC+IS (10, 33.3%) | Oral prostanoids: 3;  Parenteral prostanoids: 5;  Endothelin receptor antagonists: 5;  Phosphodiesterase 5 inhibitors: 6 | ND | Tapered PNL and AZA (2mg/kg/day) | Short-term therapeutic response^&^ , all cause and PAH related death |
| Qian et al., 2019  China [55] | N=310 (SLE-PAH)  Age: 35.0±10.1  F: 99.4% | no | i) GCs: 308 (99.4%);  ii) IS: 287 (92.6%) including 191 (61.7%) on CYC;  137 (44.3%) on≥2 ISs | PAH-targeted therapy: 215 (69.4%) including 176 (56.7%) on ERA; 180 (58.1%) on PDE5i; 21 (7.0%) on Prostacyclin analogue;  ≥2 PAH-targeted therapy: 60 (19.5%) | ND | ND | mortality and treatment goal achievement (TGA)^Δ^ |
| Distler et al, 2024 Europe [56] | N=607 CTD-PAH including 390 SSc, 90 other CTDs, 60 UCTD, 34 SLE, and 33 MCTD  Age (SLE-PAH patients): 51±18  F (SLE-PAH patients): 79.4% | no | ND | SLE-PAH patients:  Initial ERA monotherapy: 6 (18%);  Initial PDE5i monotherapy: 15 (44%);  Initial combination of ERA and PDE5i: 9 (27%) | ND | ND | Overall mortality;  Estimated survival rate at 1, 3, and 5 years;  Mean survival time |
| Li et al, 2024 China [57] | N=236 SLE-PAH  Age: 35.3±8.3  F: 97.5% | no | GC: 225 (95.3%);  IS: 198 (83.9%) | PAH-targeted therapy: 173 (73.3%)  ERA: 118 (50%);  PDE5i: 115 (48.7%);  PCA: 5 (2.1%) | ND | ND | Survival |
| Luppino-Assad et al, 2025  Brazil [58] | N=28 SLE-PAH  Age: median (interquartile range) 39.1 (25.1-44.9) in the reversion group, and 36.3 (29.5-41.6) in the non-reversion group^§§^  F: 92.9% | no | GC: 28 (100%), including GC pulse 12 (42.9%)  CYC: 19 (67.9%);  AZA: 6 (21.4%);  MMF: 4 (14.3%)  Reversion group vs non-reversion group^§§^:  GC: 100% vs 100%  GC pulse: 44.4% vs 42.1% (p=1.000);  CYC: 60.0% vs 46.4% (p=0.678);  AZA: 20% vs 22.2% (p=0.800)  MMF: 20% vs 11.1% (p=0.667) | PAH-targeted therapy: 21 (75%), and all of them used PAH-targeted combination therapy  Reversion group vs non-reversion group^§§^:  PAH-targeted therapy: 40% vs 94.4% (p=0.007) | ND | ND | PAH reversal, defined as normalisation of sPAP (<40mmHg) on TTE or of mPAP<20mmHg on RHC after IS therapy. |
| **Case series and case report** | | | | | | | |
| Robbins et al., 2000  USA [59] | N=6 (SLE-PAH)  Age: 26-34 (range)  F: 100% | no | PNL alone: 2 (33.3%);  PNL+AZA: 2 (33.3%);  PNL+ HCQ: 1 (16.7%) | Epoprostenol: 6 (100%) | Anticoagulants: 6 (100%);  CCB: 4 (66.7%) | Epoprostenol: 6 (100%) | NA |

\# PH was defined when the diagnosis was made by echocardiography, and PAH was defined when the diagnosis was made by RHC.

† Clinical worsening was defined as hospitalisation, decrease in 6MWD or worsening of FC due to PH, start of new PH treatment or death.

¶ Adjudicated clinical failure was defined as the first occurrence of death, hospitalisation for worsening PAH , disease progression according to 6MWD and WHO functional class or unsatisfactory long-term clinical response according to 6MWD and WHO functional class.

‡ Satisfactory clinical response to therapy was defined as 10% improvement in 6MWD compared with baseline, with improvement to or maintenance of WHO functional class I or II symptoms, and no events of clinical worsening before or at the week 24 visit.

* Morbidity/mortality event was defined as disease progression or worsening of PAH that resulted in hospitalisation, initiation of parenteral prostanoid therapy or long-term oxygen therapy, the need for lung transplantation or balloon atrial septostomy, or death from any cause. Disease progression was defined as a ≥15% decrease in 6MWD from baseline, confirmed by a second test on a different day, and worsening in WHO functional class (for patients in functional class II/III at baseline) or need for additional PAH therapy (for patients in functional class III/IV at baseline).

⁑ The proactive intense care strategy includes (i) an education and training course on the topic of SLE-PAH for emergency department (ED) physicians; (ii) a SLE-PAH patient triage protocol with prompt specialist consultation and admission; and (iii) intensive care with prompt initiation of combination PAH-targeted therapy, that is, at least two drugs from the three categories as represented by iloprost, bosentan and sildenafil.

$ Clinical worsening was defined as the combined end point of death, lung transplantation, hospitalisation for pulmonary hypertension, lack of clinical improvement or PAH worsening leading to discontinuation, need for epoprostenol treatment.

\# Reduction of SPAP from baseline to 6 months including 3 categories: (a) satisfactory if the decrease in SPAP was ≥20% or if this pressure reached values below 30mmHg, b) null response if the decrease in SPAP was < 20% and this pressure did not reach normal values, and c) worsening if there was an increase in SPAP.

Δ TGA was defined as when all of the following four aspects were reached: 1) Clinical symptoms: no signs of right heart failure, syncope or progression; 2) WHO FC I or II or 6MWD >380–440 m; 3) serology: BNP <50 ng/L or NT-proBNP <300 ng/L; and 4) cardiac imaging: normal right atrial area according to echocardiography.

& Short-term therapeutic response was defined as WHO Functional Class I or II with at least 1 class improvement 3 months after the introduction of first-line IS treatment.

§ Responder was defined as patients classified within NYHA FC I or II, showing hemodynamic improvement after receiving the last dose of cyclophosphamide. Hemodynamic improvement was defined as mPAP of ≤40 mm Hg and/or normal CI.

** Response to the treatment of PH was defined as: markedly effective when post-RVSP was ≤ 40 mm Hg; moderately effective when post-RVSP > 40 mm Hg and ΔRVSP < 0.5; slightly effective when ΔRVSP was between 1.5 and 0.5, but clinical signs and symptoms disappeared; no change, when ΔRVSP was between 1.5 and 0.5, and clinical signs and symptoms neither disappeared nor deteriorated; deteriorated, when ΔRVSP was ≥ 1.5 or clinical signs and symptoms deteriorated.

\## Responders to immunosuppressive therapy were defined as patients who remained in NYHA functional class I or II and showed continuous hemodynamic improvement after at least 1 year of immunosuppressive therapy without addition of PAH-targeted therapy.

§§ PAH reversal was defined by normalisation of sPAP (<40mmHg) on TTE or of mPAP<20mmHg on RHC after IS therapy. Based on this reversal criterion, patients were classified into reversion and non-reversion groups.

**Abbreviation:** AZA: azathioprine; CCB: calcium channel blockers; CsA: cyclosporin; CTD: connective tissue disease; CYC: cyclophosphamide; CVVH: continuous veno-venous haemofiltration; F: female; FC: functional class; HCQ: hydroxychloroquine; IABP: intra-aortic balloon pump counter pulsation; ICU: intensive care unit; IV: intravenous; IS: immunosuppressant; LEF: leflunomide; methyl-PNL: methylprednisolone; MCTD: mixed connective tissue disease; MMF: mycophenolate; mPAP: mean pulmonary arterial pressure; MTX: methotrexate; 6MWD: 6-minute walk distance; NA: not applicable; ND: not described; NSAIDs: non-steroids anti-inflammatory drugs; NT-proBNP: N-terminal pro brain natriuretic peptide; PAH: pulmonary arterial hypertension; PAP: pulmonary arterial pressure; PBC: primary biliary cirrhosis; PH: pulmonary hypertension; PNL: prednisolone; pSS: primary SjÖgren’s syndrome; PVR: pulmonary vascular resistance; RA: rheumatoid arthritis; RHC: right heart catheter; RTX: rituximab; RVSP: right ventricular systolic pressure; SLE: systemic lupus erythematosus; SSc: systemic sclerosis; Tac: tacrolimus; WHO: World Health Organisation.

**Supplementary Table 5.** **Pulmonary arterial hypertension-treatment response and outcomes**

| **Study ID** | **Treatment response** | | | | | | **Recurrence** |
| --- | --- | --- | --- | --- | --- | --- | --- |
|  | **Functional class** (I, II, III, IV) | **6MWD** (m) | **PAP** (mmHg) | **CO** (L/min) or **CI** (L/min/m2) | **BNP/NT-proBNP** (pg/mL) | **Other endpoints** |  |
| **RCTs or Post-hoc study/ subgroup analysis of RCTs** | | | | | | | |
| Oudiz et al., 2004  Multinational [37] | ND | Chane from baseline to 12 weeks in CTD-PAH patients: +24±12 vs +3±8, p=0.055 | Change in mPAP from baseline to 12 weeks in CTD-PAH patients: -3±1 vs -1±1, p=0.095 | Change in CI from baseline to 12 weeks in CTD-PAH patients: 0.2±0.1 vs -0.1±0.1, p=0.007 | ND | Change in Borg dyspnea score from baseline to 12 week in CTD-PAH patientss: -0.6±0.5 vs +0.2±0.5, p=0.168;  Change in Dyspnea-fatigue rating in CTD-PAH patients: 0.9±0.2 vs 0±0.3, p=0.014;  Proportion of achieving improvement in 6MWD,  Borg dyspnea score, or both at 12 weeks in CTD-PAH patients: greater in the treprostinil group, p=0.022;  Change in physical dimension score of the Minnesota  Living with Heart Failure Questionnaire in CTD-PAH patients: -5.6±1.8 vs -1.1±1.5, p=0.075  Three patients in the treprostinil group presented with a gastrointestinal hemorrhage without adverse clinical consequences. | ND |
| Gonzalez-Lopez et al., 2004  Mexico [38] | FC improved from baseline to 6 months in SLE-PAH patients: CYC group: yes (p=0.02) vs Enalapril group: no (p=0.08) | ND | Decrease of eSPAP from baseline to 6 months in SLE-PAH patients: 15 (­ 8 to 28) mmHg vs 7 (­20 to 20) mmHg, p=0.04;  Satisfactory response in reduction of eSPAP in SLE-PAH patients: 12/15 (80%) vs 12/18 (66.7%);  Satisfactory response in reduction of eSPAP in SLE patients with SPAP≥35 at baseline: 11 (100%) vs 3/9 (30%). | ND | ND | AE (CYC vs Enalapril) in SLE-PAH patients:  infections (RR 1.6; 95% CI 1.001–2.47);  gastrointestinal side effects (RR 14.6; 95% CI 2.15–99.68). | ND |
| Denton et al., 2006 (Study 351 & BREATH)  Multinational [39] | Extension trial:  FC improved: 10/40 (25%) CTD-PAH patients on Bosentan monotherapy | RCT stage: Change from baseline to 12 or 16 weeks in CTD-PAH patients: +19.5 (95% CI -3.2 to 42.2) vs -2.6 (95%CI -54.0 to 48.7), p=NS;  Extension trial: +14.7 (95% CI -11 to 40) | ND | ND | ND | RCT stage:  Cumulative event free survival in CTD-PAH patients: 95.4% vs 90.9% at 12 weeks; 90.3% vs 86.4% at 16 weeks;  Extension trial: survival in CTD-PAH patients: 85.9% at 1 year and 73.4% at 2 years | ND (1.8±0.8 years) |
| Badesch et al., 2007 (SUPER-1)  Multinational [40] | FC improved by at least 1 class from baseline to 12 weeks (20mg, 40mg, 80mg vs placebo) in CTD-PAH patients: 29%**, 40%**, 42%** vs 5%, **p<0.003 vs placebo | Mean increase from baseline to 12 weeks (20mg, 40mg, 80mg vs placebo) in CTD-PAH patients: 42** (95% CI 20-64), 36** (95% CI 14-58), 15 (95% CI -24-54) vs 13 (95%CI -36-10),  **p<0.01 vs placebo | mPAP change from baseline to 12 weeks (20mg, 40mg, 80mg vs placebo) in CTD-PAH patients:  -4.6** (-8.7, -0.6), -2.8 (-5.4, -0.1), -3.2 (-7.9, 1.5) vs 1.4 (-0.8, 3.6),  ** p<0.05 vs placebo | CO change from baseline to 12 weeks (20mg, 40mg, 80mg vs placebo) in CTD-PAH patients:  0.8 (-0.02, 1.5), 0.4 (-0.5, 1.3), 0.2 (-0.4, 0.8) vs 0.08 (-0.3, 0.4) | ND | PVR change from baseline to 12 weeks (20mg, 40mg, 80mg vs placebo) in CTD-PAH patients: -243** (-408, -77), -144 (-278, -10), -156 (-267, -46) vs -19 (-106, 68),  **p<0.05 vs placebo | ND |
| Girgis et al., 2007 (STRIDE-1 and STRIDE-1X)  USA & Canada [41] | STRIDE-1:  FC improved in CTD-PAH patients: 8/33 (24%) vs 1/9 (11%) (p=0.14); FC deteriorated: 0/33 (0%) vs 1/9 (11%).  STRIDE-1X:  FC improved in CTD-PAH patients: 16/41 (39%); FC deteriorated: 2/41 (4.9%); FC I/II 12/41 (29%) at baseline vs 26/41 (63%) at the end of the extension trial, p<0.001 | Change of 6MWD from baseline to 12 weeks in CTD-PAH patients: +20±52 vs -38±84, p=0.027 | Change of mPAP from baseline to 12 weeks in CTD-PAH patients: -4 vs +4, p=0.05 | Change of CI from baseline to 12 weeks in CTD-PAH patients: -0.6 vs +3.6, p<0.01 | ND | Change of SF-36 physical functioning domain score from baseline to 12 weeks in CTD-PAH patients: +3.5±7.5 vs -2.2±9.3, p=0.042;  Mortality during the extension trial in CTD-PAH patients: 2/41 (4.9%). One died of myocardial infarction and the other died of infection and acute  hypoxaemic respiratory failure.  Elevation of hepatic  transaminase levels occurred in the extension stage in two patients. | Recurrence in CTD-PAH patients: 1/41 (2.4%) during the extension trial (26 weeks, range 13 days - 56 weeks) |
| Coghlan et al., 2017 (AMBITION)  Multinational [42] | ND | Increase of 6MWD in CTD-PAH patients: 42.0 vs 24.3;  ≥15% decrease of 6MWD from baseline to 24 weeks in CTD-PAH patients:  28/99 (28%) vs  29/81 (36%) | ND | ND | NT-proBNP decrease from baseline to 24 weeks in CTD-PAH patients: −60.4% vs −43.1% | Primary endpoint: clinical failure in CTD-PAH patients: 20/103 (19%) vs 30/84 (36%), HR: 0.43 (95% CI 0.24 to 0.77);  Secondary: Rate of satisfactory clinical response at week 24 in CTD-PAH patients: 35% vs 29%, OR 1.321 (95% CI 0.680 to 2.565);  Death in CTD-PAH patients: 6/103 (6%) vs 6/84 (7%), HR: 0.89 (0.29-2.77) | ND |
| Gaine et al., 2017  (GRIPHON)  Multinational [43] | ND | Median change from baseline to 26 weeks in SLE patients: 16 (-10-35) vs 1 (-100-21).  Treatment effect (95% CI) in SLE patients: 17 (-8, 44) m | ND | ND | NT-proBNP change from baseline to 26 weeks in SLE patients: -63.0 (-380.0, -15.0) vs -36.5 (-93.5, 93.0).  Treatment effect (95% CI) in SLE patients: -64.5 (-280, 20) | Composite endpoint of morbidity/mortality in SLE patients: 11 (24.4%) vs 13 (35.1%), HR 0.66 (0.30–1.48);  All-cause death in SLE patients: 4 (8.9%) vs 7 (18.9%) | ND |
| Humbert et al., 2017 (PATENT-1 and 2)  Multinational [44] | PATENT-1 trial:  FC improved from baseline to 12 weeks in CTD-PAH patients: 28 % vs 17%;  stablised: 75% vs 58%; worsened: 3% vs 25%  PATENT-2 trial: FC  improved/stabilised/worsened in 36%/59%/6% of CTD-PAH patients, respectively at 2 years | Mean change of 6MWD from baseline to 12 weeks: 18±51 vs -8±110;  PATENT-2 trial: mean 6MWD further improved within 2 years. | Mean change of mPAP from baseline to 12 weeks in CTD-PAH patients: -7±7.1 vs -4.6±10.9 | Mean change of CI from baseline to 12 weeks in CTD-PAH patients: 0.5±0.5 vs -0.2±0.6 | Mean change of NT-proBNP from baseline to 12 weeks in CTD-PAH patients: 274±2576 vs 54±778 | Mean change of EQ-5D from baseline to 12 weeks in CTD-PAH patients: -0.01±0.24 vs -0.02±0.40 | In PATENT-2: clinical worsening (hospitalisation, decrease in  6MWD or worsening of FC due to PH, start of new PH treatment or death) in CTD-PAH patients: 27/94  (29%) within 2 years |
| **Non-randomised trial** | | | | | | | |
| Chen et al., 2015  China [45] | FC change favoured the proactive intense care (6, 54.5% with class improved by one grade in SLE-PAH patients; details in historical control group ND) | ND | ND | ND | ND | Proactive intense care vs historical control in SLE-PAH patients: Survival at 3 months: 8/11 (72.7%) vs 3/11 (27.3%), p=0.033; Death in ED within 24 h: 1/11 (9.1%) vs 3/11 (27.3%), p=0.586. Combination therapy vs non-combination therapy: Survival at 3 months: 7/8 (87.5%) vs 4/14 (28.6%), p=0.0099 | ND |
| **Prospective noncontrolled cohort study** | | | | | | | |
| Cozzi et al., 2006  Italy [46] | WHO FC at baseline in CTD-PAH patients:  borderline between II and III: 6 (46.2%);  III: 7 (53.8%)  WHO FC at 1 year in CTD-PAH patients:  II: 4 (30.8%);  borderline between II and III: 6 (46.2%);  III: 3 (23.1%) | 6MWD improved at 1 year in CTD-PAH patients: 7 (54%) including 2 SLE patients whose 6MWD improved from 672 m and 464 m at baseline to 675 and 520 at 6 months, respectively | mPAP decreased at 1 year in CTD-PAH patients: 6/11 (54.5%) including 2 SLE patients whose mPAP decreased from 34 and 33 mmHg at baseline to 30 and 30 at 6 months, respectively | ND | ND | RVSP decreased at 1 year in CTD-PAH patients: 9 (69%) including 2 SLE patients whose RVSP decreased from 63 mmHg and 82 mmHg at baseline to 54 and 70 at 6 months, respectively | ND |
| **Observational studies** | | | | | | | |
| Tanaka et al., 2002  Japan [47] | ND | ND | ND | ND | ND | Treatment response in SLE-PAH patients with right heart failure: 7/8 (87.5%) including i) marked response: 4/8 (50%); ii) moderate response: 3/8 (37.5%); iii) slight response: 1/8 (12.5%)*.  One SLE-PAH patient died of infection at 136 months. | 1 SLE-PAH patient relapsed once at 4 months and 1 SLE-PAH patient relapsed twice at 15 and 24 months, respectively. Both patients were successfully treated with pulsed methyl-PNL, oral PNL and CYC. |
| Sanchez et al., 2006  France [48] | Among 8 (28.6%) responders (5 SLE and 3 MCTD): FC I: 0 (0%) at baseline to 4 (50%) after IS treatment; FC II: 3 (38%) to 4 (50%); FC III: 5 (62%) to 0 (0%), all p values <0.05 | Among 8 responder (5 SLE and 3 MCTD), 294±118 at baseline to 482±113 after IS treatment, P<0.05 | Among 8 responders (5 SLE and 3 MCTD), mPAP: 49±16 at baseline to 34±11 after IS treatment, p<0.05 | ND | Among 8 responders (5 SLE and 3 MCTD), CI: 3.1±0.5 at baseline to 3.5±0.5 after IS treatment | Responder in CTD-PAH patients: 5/13 (38%) in SLE; 3/8 (37.5%) in MCTD; and 0/6 (0%) in SSc.  Death in CTD-PAH patients: 11/28 (39.2%) including right heart failure (6), sepsis (2), stroke (2), and sudden death (1). All death happened in non-responders. | 16/28 (57.1%) CTD-PAH patients required second-line PAH target therapy due to persistent severe PAH including 1/8 (12.5%) responder to first-line treatment and 15/20 (75%) non-responders. |
| Heresi et al., 2007  USA [49] | FC I/II in SLE-PAH patients: 0 (0%) at baseline to 8/12 (66.7%) at the end of follow-up (mean 41±25 months) | Median increase of 6MWD at the end of follow-up (mean 41±25 months) in SLE-PAH patients: 139 m (95% CI 36-259)  6MWD improvement in SLE-PAH patients: 6/7 (85.7%) | Median decrease of mPAP at the end of follow-up (mean 41±25 months) in SLE-PAH patients: 18 mmHg (95% CI 8-29) (p=0.014)  mPAP improvement in SLE-PAH patients: 4/5 (80%) | Median increase of CI at the end of follow-up (mean 41±25 months) in SLE-PAH patients: 1.44 (95% CI 0.76-2.08) (p=0.016)  CI improvement: 4/5 (80%) | ND | Median decrease of RVSP at the end of follow-up (mean 41±25 months) in SLE-PAH patients: 22mmHg (95% CI 6-36) (p=0.012)  Mortality during follow-up in SLE-PAH patients: 0 (0%) | ND |
| Jais et al., 2008  France [50] | ND | ND | ND | ND | ND | Responder (immunosuppressive alone group vs combination group) in SLE/MCTD-PAH patients: 8 (50%) vs 4 (57.1%).  In immunosuppressive therapy alone group, SLE/MCTD-PAH patients in functional class I/II and/or a CI >3.1 at baseline more likely to benefit from immunosuppressive therapy. | ND |
| Miyamichi-Yamamoto et al., 2011  Japan [51] | ND | ND | mPAP decreased from 39.5±9.1 at baseline to 28.9±11.0 at the end of follow-up in IIT group (CTD-PAH), p<0.01; and increased from 43.5±17.4 at baseline to 50.3±18.8 at the end of follow-up in the control group (CTD-PAH), p=0.08 | CI kept stable from baseline to the end of follow-up for both groups (CTD-PAH): 2.87±1.37 at baseline and 2.79±0.65 at the end for IIT group, p=0.82; and 2.52±0.75 at baseline and 2.71±0.83 at the end for control group, p=0.63 | BNP decreased from 201±397 at baseline to 15±16 at the end of follow-up for IIT group (CTD-PAH), p=0.11; changed from 303±290 at baseline to 361±281 at the end for the control group (CTD-PAH), p=0.66 | Responders (IIT vs conventional): 3/13 (23.1%) including 1 SLE, 1 pSS, and 1 MCTD vs 0 (0%);  Mortality (IIT vs conventional) over 30-48 months in CTD-PAH patients: 7.7% vs 62.5%;  Long-term survival significantly  better in the IIT group than in the  historical control group in CTD-PAH patients, p=0.0072 | Follow-up  Period: 914±456 days for the historical control group  and 565±431 days for the IIT group. |
| Shirai et al., 2013  Japan [52] | Epoprotenol group:  FC improved or remained from baseline to 6 months in CTD-PAH patients: 12/12 (100%)  FC II: 1 (8.3%) to 7 (58.3%);  FC III: 7 (58.3%) to 6 (50%);  FC IV: 4 (33.3%) to 1 (8.3%) | ND | Epoprotenol group:  mPAP level decreased by 26% from baseline to 6 months in CTD-PAH patients,  p= 0.006  mPAP decrease in CTD-PAH patients: 10/12 (83.3%) | Epoprotenol group (CTD-PAH):  CI level increased by 36 % from baseline to 6 months, p= 0.02  CI improvement: 10/12 (83.3%) | Epoprotenol group (CTD-PAH):  BNP level showed a trend of decrease over 6 months, p=0.06  BNP decrease: 5/12 (41.7%) | Death in Epoprotenol group (CTD-PAH): 11 (68.8%) during mean 52 ± 27 (range 9–90) months of follow-up;  Cumulative survival rates at 1,2 and 3 years between  Epoprotenol group vs control group in CTD-PAH patients: 69%, 69% and 55% vs 56%, 25%, and 6%, respectively, log rank p=0.02 | ND |
| Kommireddy et al., 2015  India [53] | FC improved from baseline to 6 months in SLE-PAH patients: 22/24 (91.7%)  FC I: 5 (20.8%) to 14 (58.3%);  FC II: 6 (25%) to 7 (29.2%);  FC III: 8 (33.3%) to 1 (4.2%);  FC IV: 5 (20.8%) to 0 (0%)  Asymptomatic: 0 (%) to 2 (8.3%) | mean 6 MWD improved from 312.04 at baseline to 420.16 at 6 months in SLE-PAH patients | eSPAP decreased from baseline in SLE-PAH patients: 21/24 (87.5%); mean eSPAP decreased from 59.3±18.7 at baseline to 43.3±20.9 at 6 months, p<0.0001  In SLE-PAH patients receiving immunosuppressive therapy only, mean eSPAP decreased from 39.8±7.0 to 34.3±8.3 at 6 months, p=0.04 | ND | ND | Responder in SLE-PAH patients: 11 (45.8%)  Responder in  immunosuppressive therapy only patients vs combination therapy patients: 0/4 (0%) vs 11/20 (55%)  Mortality in SLE-PAH patients: 2/24 (8.3%) during median 12 months (range 6-53) of follow-up. One was due to PAH and the other was due to septic shock. | No recurrence reported, but 1/24 PAH-related death within median 12 (range 6-53) of follow-up |
| Yasuoka et al., 2018  Japan [54] | FC improved at least 1 grade to Class I or II in SLE/MCTD/pSS patients: 16 (53%) | ND | mPAP decreased from baseline to 3 months in SLE/MCTD/pSS patients: from 48.6±7.8 to 33.6±10.7 vs from 52.1±10.7 to 48.9±5.1 (9 short-term responders vs 7 non-responders) | CI increased from baseline to 3 months in SLE/MCTD/pSS patients: from 2.6±1.0 to 3.4±1.6 vs from 1.9±0.6 to 2.4±2.3 (9 short-term responders vs 7 non-responder) | BNP decreased from baseline to 3 months in SLE/MCTD/pSS patients: from 545±546 to 122±180 vs from 1,460±2,150 at to 603±599 (9 short-term responders vs 7 non-responders) | Short-term responders in SLE/MCTD/pSS patients: 16 (53%);  All-cause death in SLE/MCTD/pSS patients: 16 (53%), 6/16 (37.5%) vs 10/14 (71.4%) (short-term responder vs non-responder), log-rank test p=0.06.  PAH-related death in SLE/MCTD/pSS patients: 14 (46.7%), 5/16 31.3%) vs 9/14 (64.3%) (short-term responder vs non-responder), log rank test p=0.04. | No recurrence reported, but 14 PAH-related death within 5.5±4.3 years of follow-up |
| Qian et al., 2019  China [55] | ND | ND | ND | ND | ND | Treatment goal achievement (TGA) within median 24 months of follow-up in SLE-PAH patients: 115 (43.7%)  Mortality within 24 months of follow-up in SLE-PAH patients: 42 (14.9%);  Estimated 1-, 3- and 5-year survival in SLE-PAH patients: 92.1%, 84.8% and 72.9%, respectively | ND |
| Distler et al, 2024 Europe [56] | ND | ND | ND | ND | ND | SLE-PAH patients:  Overall mortality over 5 years: 8 (23.5%);  Estimated 1-, 3-, and 5-year survival: 97.1%, 77.3% and 60.6%;  Estimated mean survival time: 4.1 years (95% CI 3.6, 4.6) | ND |
| Li et al, 2024 China [57] | ND | ND | ND | ND | ND | Patients with mild SLE-PAH (mPAP 21–24 mmHg and PVR 2-3 wood units) had a better survival rate than those with conventional SLE-PAH (mPAP≥25 mmHg and PVR>3 wood units).  Among patients with mild SLE-PAH, no significant difference  in survival between patients with or without PAH targeted therapy. | ND |
| Luppino-Assad et al, 2025  Brazil [58] | Proportion in FC III/IV before vs after IS treatment in SLE-PAH patients:  80% vs 0% in the reversion group^§^ (p=0.003);  88.9% vs 23.5% in the non-reversion group^§^ (p<0.001) | ND | mPAP before vs after IS treatment in SLE-PAH patients:  28.0 (28.0-45.8) vs 18.5 (16.0-20.0) in the reversion group^§^ (p=0.004)  55.0 (50.0-57.5) vs 46.5 (43.0-53.0) in the non-reversion group^§^ (p=0.160) | CI before vs after IS treatment in SLE-PAH patients:  3.2 (1.9-3.7) vs 3.4 (1.8-4.2) in the reversion group^§^ (p=0.123)  1.9 (1.5-2.4) vs 2.1 (1.7-2.9) in the non-reversion group^§^ (p=0.433) | BNP before vs after IS treatment in SLE-PAH patients:  32.0 (16.5-81.8) vs 26.5 (15.5-45.5) in the reversion group^§^ (p=0.438);  421.0 (192.3-641.0) vs 111.0 (28.5-500.0) in the non-reversion group^§^ (p=0.018) | PAH reversal in SLE-PAH patients: 10 (35.7%) out of the total 28 patients;  Estimated survival over 10 years between reversion and non-reversion groups^§^ in SLE-PAH patients: 100% vs 66.7% (p=0.047) | ND |
| **Case series and case reports** | | | | | | | |
| Robbins et al., 2000  USA [59] | FC improved from III/IV at baseline to I/ II at the end of follow-up (9-16 months) in SLE-PAH patients: 6 (100%) | ND | Mean mPAP decreased from 57±9 at baseline to 33±14 at 9-16 months  mPAP improvement in SLE-PAH patients: 4/4 (100%) | Mean CO increased from 3.7±1.2 to 5.9±0.6 at 9-16 months  CO improvement in SLE-PAH patients: 4/4 (100%) | ND | PVR decreased from 14.0±6.6 at baseline to 4.0±1.5 at 9-16 months  PVR improvement in SLE-PAH patients: 4/4 (100%)  Mortality in SLE-PAH patients: 0/6 (0%) | No recurrence within 9-16 months |

* Treatment effectiveness was only assessed in 8 patients with right heart failure and immunosuppressive therapy.

§ PAH reversal was defined by normalisation of sPAP (<40mmHg) on TTE or of mPAP<20mmHg on RHC after IS therapy. Based on this reversal criterion, patients were classified into reversion and non-reversion groups.

**Abbreviation:** CI: cardiac index; 95% CI: 95% confidence interval; CO: cardiac output; CYC: cyclophosphamide; eSPAP: estimated systolic pulmonary arterial pressure; EQ-5D: EuroQol five dimensions questionnaire; FC: functional class; HR: hazard ratio; MCTD: mixed connective tissue disease; methyl-PNL: methylprednisolone; mPAP: mean pulmonary arterial pressure; 6MWD: 6-minute walk distance; ND: not described; NT-proBNP: N-terminal pro brain natriuretic peptide; PAH: pulmonary arterial hypertension; PAP: pulmonary arterial pressure; PNL: prednisolone; PVR: pulmonary vascular resistance; RCT: randomised clinical trial; RVSP: right ventricular systolic pressure; SF-36: short form (36) health survey; SLE: systemic lupus erythematosus; SSc: systemic sclerosis; Tac: tacrolimus; WHO: World Health Organisation.

**Supplementary Table 6. Pleuritis**

| **Study** | **Population** | **Intervention** | | | **Response** | **Recurrence within follow-up duration** |
| --- | --- | --- | --- | --- | --- | --- |
|  |  | **Immunosuppressive therapy** | **Other therapy** | **Maintenance** |  |  |
| **Observational studies** | | | | | | |
| Palavutitotai et al., 2014  Thailand [60] | N=119 (127 episodes of pleural effusion including 12 TB pleuritis, 9 parapneumonic effusions, 66 lupus pleuritis, 18 transudate, and 22 inconclusive)  Age: 29.7±12.5  F: 95% | Baseline treatment including antimalarial or immunosuppressive agents, corticosteroids, or NSAID for all patients: 60%  For lupus pleuritis:  corticosteroids: 93%;  NSAIDs: 7%;  For infectious pleuritis: specific antibiotics | For lupus pleuritis:  thoracocentesis, pleural biopsy, and bronchoscopy with lavage: 35/66 (53.3%) | ND | Lupus pleuritis:  response: 94%;  non-response: 1%;  unknown outcome: 5%  TB pleuritis:  response: 75%;  non-response: 25%  Parapneumonic effusions:  response: 100%  Transudate:  response: 89%;  unknown: 11%  Inconclusive:  response: 68%;  non-response: 9%;  unknown: 23% | Recurrence happened in 14% of patients with lupus pleuritis |

**Abbreviation:** F: female; ND: not described; NSAIDs: non-steroids anti-inflammatory drugs.

**Supplementary Table 7.** **Acute lupus pneumonitis**

| **Study** | **Population** | **Intervention** | | | **Response** | **Recurrence within follow-up duration** |
| --- | --- | --- | --- | --- | --- | --- |
|  |  | **Immunosuppressive therapy** | **Other therapy** | **Maintenance** |  |  |
| **Case series** | | | | | | |
| Wan et al., 2016  Malaysia [61] | N=5  Age: (mean) 20.8  F: 100% | Regimens:  High dose GC+AZA: 1 (20%);  IV methyl-PNL+ IV CYC: 1 (20%);  IV methyl-PNL+ IV CYC + IVIG: 3 (60%) | ICU admission: 3/5 (60%);  Ventilation: 2/5 (40%) | Corticosteroids and CYC: 1/3 (33.3%);  Corticosteroids and AZA: 2/3 (66.7%) | Responded and survived during follow-up: 3/5 (60%);  Mortality: 2/5 (40%) died of hospital-acquired pneumonia and lupus pneumonitis | No recurrence reported in 3/3 survived patients during follow-up |

**Abbreviation:** AZA: azathioprine; CYC: cyclophosphamide; F: female; IVIG: intravenous immunoglobulin.

**Supplementary Table 8. Diffuse alveolar haemorrhage**

| **Study** | **Population** | **Intervention** | | | **Response/Mortality** | **Recurrence within follow-up duration** |
| --- | --- | --- | --- | --- | --- | --- |
|  |  | **Immunosuppressive therapy** | **Other therapy** | **Maintenance** |  |  |
| **Observational studies** | | | | | | |
| Barile et al., 1997  Mexico [62] | N=34  Age: (mean) 34.5  F: 94.1% | GC: 34 (100%) including three regimens:  i) oral prednisone (1mg/kg/d): 9 (26.5%)  ii) conventional IV methyl-PNL pulse (1000mg/d for 3 days): 13 (38.2%);  iii) massive IV methyl-PNL pulse (1000mg/d for 4-8 days) (for those who did not respond to three-day pulses, treatment continued to 4 to 8 days): 12 (35.3%)  IV CYC: 2 (5.9%);  PE: 2 (5.9%) (one course for each, and received  methyl-PNL pulse before and after the procedure)  Regimens: GC alone (30, 88.2%); GC+CYC (2, 5.9%); GC+PE (2, 5.9%) | Antibiotics: 32 (94.1%);  Mechanical ventilator: 34 (100%);  ICU admission: 20 (58.9%) | ND | Mortality within 60 days: 21 (61.8%) and all died of DAH;  Survival according to corticosteroids treatment regimens:  i) oral prednisone: 0/9 (0%);  ii) conventional Methyl-PNL pulse: 4/13 (30.8%);  iii) massive Methyl-PNL pulse: 9/12 (75%)  Three patients (3/13, 23.1%) died of non-pulmonary causes during follow-up (mean 2.5 years, range 1 month-8 years) | ND |
| Badsha et al., 2004  Singapore [63] | N=22  Age: 31.6±12.0  F: 91% | High dose GC: 22 (100%) including 19 (86.3%) IV methyl-PNL pulse;  IV CYC (500mg/m^2^): 19 (86.3%)  PE (2-6 cycles): 11 (50%)  Regimens: ND | Mechanical ventilation: 14 (63.6%)  ICU admission: 22 (100%) | ND | Mortality: 8 (36%), all died of DAH  Treatment difference between Survivors (n=14) vs Death (n=8):  i) methyl-PNL pulse: 13/14 (93%) vs 6/8 (75%);  ii) IV CYC: 13/14 (93%) vs 6/8 (75%);  iii) PE: 6/14 (43%) vs 5/8 (63%);  iv) Mechanical ventilation: 6/14 (43%) vs 8/8 (100%), p=0.02 | No recurrence within mean 24 months of follow-up, but 3/14 (21.4%) developed ILD |
| Kazzaz, et al. 2015  USA [70] | N=22  Age: (average) 37  F: ND | GCs: 21 (95.5%), including methylprednisolone pulse: 10 (45%), oral prednisone (1mg/kg/d): 4 (18%), and increased prednisone dose: 7 (32%);  CYC: 11 (50%), including 2 on CYC and RTX combination;  MMF: 5 (23%)  RTX: 3 (14%) (all of them presented after 2007).  IVIG: 3 (14%);  PE: 4 (18%) | ND | ND | Short-term survival before hospital discharge: 22 (100%);  Mortality after discharge: 6 (27.3%)  Estimated long-term survival: significantly worse in the SLE-DAH group than the control SLE without DAH group. | Recurrence: 6 (27.3%), and 5 of them happened within 6 months of the first episode of DAH. |
| Andrade et al., 2016  Portugal [64] | N=7 (10 episodes)  Age: 42.8±18.9  F: 100% | IV methyl-PNL pulse (500-1000mg/d for 3 days): 7 (100%);  IV CYC (single dose 500-800 mg/m^2^): 2 (28.6%);  MMF: 1 (14.3%);  IVIG: 1 (14.3%);  PE: 2 (28.6%)  Regimens: pulse GC alone (3, 42.9%), pulse GC+IS (CYC or CYC+MMF) (2, 28.6%), pulse GC+PE (1, 14.3%), pulse GC+PE+IVIG (1, 14.3%), | Mechanical ventilation: 5/8 episodes (62.5%);  ICU admission: 7 (100%);  Empirical antibiotics: 4/8 episodes (50%). | ND | Within mean 33 (1-78) hospital days:  Improvement: 5 (71.4%);  Infection: 6 (85.7%);  Mortality: 2 (28.6%), due to respiratory failure | ND |
| Wang et al., 2018  Taiwan [65] | N=17  Age: 38.2±15.1  F: 88.2% | High dose GC: 17 (100%) including IV methyl-PNL pulse (12, 70.6%);  IV CYC pulse: 6 (35.5%);  RTX: 4 (23.5%);  PE: 7 (41.2%)  Regimens: ND | Mechanical ventilation: 12 (70.6%);  ECMO: 3 (17.6%) | ND | Mortality: 6 (35.3%), died of respiratory failure  Treatment difference between Survivors (n=11) vs Death (n=6):  i) Methyl-PNL pulse: 7/11 (63.6%) vs 5/6 (83.3%);  ii) IV CYC: 5/11 (45.5%) vs 1/6 (16.7%);  iii) RTX: 4/11 (36.4%) vs 0/6 (0%);  iv) PE: 5/11 (45.5%) vs 2/6 (33.3%);  v) Ventilation: 6/11 (54.6%) vs 6/11 (100%)  vi) ECMO: 0/11 (0%) vs 3/6 (50%) (p=0.0294) | ND |
| Sun et al., 2020  China [66] | N=94  Age: 29 (range 21–42)  F: 87.2% | GC: 94 (100%)  including pulse methyl-PNL (74, 78.7%); high-dose IV GC (20, 21.3%)  IV CYC: 71 (75.5%);  IVIG: 67 (71.3%);  PE: 36 (38.3%)  Regimen: GC+IS: 94 (100%) | Mechanical ventilation: 50 (53.2%) | ND | Mortality within median 75 (IQR 1-83) months of follow-up: 36 (38.3%)  Mortality of patients on CYC: 18/71 (25.4%), HR 0.3 (95% CI 0.2-0.6), p<0.001  Treatment difference between Survivors (n=58) vs Death (n=36):  i) methyl-PNL pulse: 43/58 (74.1%) vs 31/36 (86.1%);  ii) IV CYC: 53/58 (91.4%) vs 18/36 (50.0%) (p<0.0001);  iii) IVIG: 43/58 (74.1%) vs 24/36 (66.7%);  iv) PE: 20/58 (34.5%) vs 16/36 (44.4%);  v) ventilation: 26/58 (44.8%) vs 24/36 (66.7%) | ND |
| Li et al, 2023  China [71] | N=124  Overall age and gender disparity was not described.  Bacterial infection vs non-bacterial infection vs non-infection groups:  Age: 37.2 ± 16.2 vs 35.1 ± 11.5 vs 32.9 ± 10.9  F: 89.7% vs 87.5% vs 88.2% | Bacterial infection (n=58) vs non-bacterial infection (n=32) vs non-infection(n=34) groups:  HCQ: 22 (37.9%) vs 5 (15.6%) vs 12 (35.3%)  GC pulse therapy: 39 (67.2%) vs 17 (53.1%) vs 18 (52.9%), p=0.274  CYC: 32 (55.2%) vs 21 (65.6%) vs 23 (67.6%), p=0.471;  Other IS included MMF, Tac, and CsA.  RTX: 2 (3.4%) vs 0 (0%) vs 0 (0%);  IVIG: 36 (62.1%) vs 17 (53.1%) vs 19 (55.9%), p=0.681;  PE: 27 (46.6%) vs 6 (18.8%) vs 12 (35.3%), p=0.032 | Overall prophylaxis antibiotic therapy: 50%  Bacterial infection (n=58) vs non-bacterial infection (n=32) vs non-infection (n=34) groups:  No significant difference in Anti-bacteria, viral, and fungal prophylaxis and empirical treatments among the three groups. | ND | Bacterial infection (n=58) vs non-bacterial infection (n=32) vs non-infection(n=34) groups:  Mortality within 180 days  after DAH occurrence:  35 (60.3%) vs 11 (34.4%) vs 10 (29.4%), p=0.006 | ND |
| Garcia-Villanueva, et al. 2024  Spain [72] | N=14  Age: 31.2 ± 15.5  F: 92.9% | GC: 12 (85.7%)  CYC (IV for 8 patients with dose of 500-1000mg, and oral for 1 patient): 9 (69.2%)  RTX: 2 (15.4%)  IVIG: 8 (57.1%)  PE (3-28 sessions): 5 (35.7%) | Concomitant antibiotics: 10 (83.3%)  ICU admission: 12 (85.7%)  Mechanical ventila­tion: 11 (84.6%) | GC in combination with HCQ, CYC or MMF | Mortality: 5/14 (35.7%), 3 (21.4%) died of infection, 1 (7.1%) died of DAH itself, and 1 (7.1%) died of catastrophic anti-phospholipid syndrome.  Survivor vs Death (9 vs 5):  Infection: 44.4% vs 60%, p=0.03;  CYC use: 62.5% vs 80%, p=0.03;  PE use: 37.5% vs 40%, p=0.685;  Mechanical ventilation: 87.5% vs 80%, p=0.03;  ICU: 88.9% vs 80%, p=0.03 | Radiological sequelae (with infiltrates on plain radiography or CT) and functional sequelae (restrictive pattern and abnormalities in carbon monoxide diffusion) in 2/14 (14.3%) within 6 months of follow-up. |
| Law, et al. 2023  Singapore [73] | N=35  Age; 40 (25–54)  F: 71.4% | GC: 35 (100%), including 28 (80%) methyl-PNL pulse 500-1000mg for 3 days), and 7 (20%) oral PNL 1mg/kg/d.  CYC: 28 (80%), including 24 (68.6%) IV and 4 (11.4%) oral.  RTX: 16 (45.7%)  IVIG: 13 (37.1%)  PE (4-28 sessions): 23 (65.7%)  Autologous haematopoietic stem cell transplant: 2 (5.7%)  Regimen:  GC+CYC+RTX+PE: 14 (40%)  GC+CYC+PE: 4 (11.4%)  GC+CYC: 8 (22.8%)  GC+PE: 4 (11.4%)  Others: 5 (14.3%) | Mechanical ventilation: 22 (62.9%) | ND | Clinical remission of DAH: 26 (74.3%)  Time to DAH remission: 12 (6-46) days  Mortality: 14 (40%), 8 (57.1%) died of DAH, 4 (28.6%) died of infection, and 2 (14.3%) died of other causes.  Time to death from DAH: 10.5 (2–57) days  Patients receiving GC+CYC+RTX+PE vs Patients receiving GC+PE:  Time to death: 162 vs 14 days  (p = 0.0026)  Survivor vs Death (patients who survived or died of DAH):  Pulse GC: 23 (85.2%) vs 5 (62.5), p=0.31  CYC use: 24 (88.9%) vs 3 (37.5%), p=0.007  RTX use: 13 (48.1%) vs 3 (37.5%), p=0.700  IVIG use: 11 (40.7%) vs 2 (25.0%), p=0.680  PE use: 17 (63.0%) vs 6 (75.0%), p=0.690  Autologous haematopoietic stem cell transplant: 1 (3.7%) vs 1 (12.5%), p=0.41  Mechanical ventilation use: 16 (59.3%) vs 6 (75.0%), p=0.680 | ND |
| **Case series and case reports** | | | | | | |
| Zamora et al., 1997  USA [67] | N=15 (19 episodes)  Age: 27 (range 19-44)  F: 66.7% | Pulsed methyl-PNL (500-2000mg/d for 2-6 days): 18/19 episodes (94.7%);  IV CYC (500-2000mg, single dose): 13/19 (68.4%) episodes;  AZA: 5/19 (26.3%) episodes;  PE: 7/19 (36.8%)  Regimens: GC alone (1, 5.3%), IS alone (1, 5.3%), GC+IS (10, 52.6%), GC+PE (2, 10.5%), GC+IS+PE (5, 26.3%) | ND | ND | Mortality during hospital days: 8/15 patients (53.3%), 7 (46.7%) died of DAH including 1 with recurrent DAH and 1 died with unknown causes.  Mortality between those with and without CYC: 70% vs 11% (p=0.02);  Mortality between those with and without PE: 57.1% vs 33.3%  Mortality between those with and without ventilation: 62% vs 0% (p=0.02);  Mortality between those with and without infection: 78% vs 20% (p=0.02) | DAH recurrence happened in 3/15 (20%) patients within follow-up duration (range 1-108 months) |
| Santos-Ocampo et al., 2000  USA [68] | N=7 (11 episodes)  Age: 31.1 (range 19-44)  F: 85.7% | i) Prednisone (1-3mg/kg/d): 2/11 (18.2%);  ii) IV methyl-PNL pulse (240-1000mg/d for 3 days): 9/11 episodes (81.8%) including 2 (18.2%) with repeated methyl-PNL pulse;  iii) IV or oral CYC (2-5mg/kg/d): 8/11 (72.7%) episodes;  iv) PE (3-4 courses): 5/11 (45.5%)  Regimens: GC alone (3, 27.3%), GC+CYC (3, 27.3%), GC+CYC+PE (5, 45.4%) | Mechanical ventilation: 4/11 episodes (36.3%);  Concurrent antibiotics: 10/11 (90.9%) episodes | PNL (40-60mg/d): 11/11 (100%) episodes;  CYC (50-150mg/d): 7/11 (63.6%) episodes;  AZA (50-200mg/d): 2/11 (18.2%) episodes | Survival within hospital days: 11/11 (100%) episodes with a mean duration of 7 days in DAH (range 2-21 days) | no DAH recurrence within mean 7.8 months of follow-up  (range 1-22 months)  : 7/7 (100%) |
| Canas et al., 2007  Colombia [69] | N=7  Age: 24.3 (range 4–  51)  F: 71.4% | i) IV methyl-PNL pulse (10mg/kg/d for 3 days): 7 (100%);  ii) IV CYC pulse (750mg/m^2^): 7 (100%);  iii) PE: 4 (57.1%)  Regimens: pulse GC+CYC (3, 42.9%), pulse GC+CYC+PE (4, 57.1%) | Mechanical ventilation: 6 (85.7%);  ICU admission: 7 (100%) | ND | Mortality: 1 (14.3%) died of recurrent DAH. | ND |

**Abbreviation:** AZA: azathioprine; CYC: cyclophosphamide; DAH: Diffuse alveolar haemorrhage; ECMO, extracorporeal membrane oxygenation; F: female; HR: hazard ratio; ICU: intensive care unit; IV: intravenous; IS: immunosuppressant; IVIG: intravenous immunoglobulin; methyl-PNL: methylprednisolone; MMF: mycophenolate; ND: not described; PE: plasma exchange; PNL: prednisolone; RTX: rituximab.

**Supplementary Table 9. Interstitial lung disease**

| **Study** | **Population** | **Comparator**  **(patient number in each group)** | **Intervention** | | | **Response** | **Recurrence within follow-up duration** |
| --- | --- | --- | --- | --- | --- | --- | --- |
|  |  |  | **Immunosuppressive therapy** | **Other therapy** | **Maintenance** |  |  |
| **Prospective controlled cohort study** | | | | | | | |
| Wang et al.., 2022  China [74] | N=111 CTD-ILD including 30 SSc, 51 IIM, 17 RA, and 13 other CTDs (SLE, pSS, UCTD) | Pirfenidone group vs control group  ---overall CTD-ILD: 56 vs 55  ---SSc: 14 vs 16  ---IIM: 25 vs 25;  ---RA: 10 vs 7;  Other CTD including SLE: 7 vs 6 | GC: 53 (94.6%) vs 53 (96.4%);  median (range) GC dose: 15 (5-32.5) mg/d vs 15 (7.5-20.0) mg/d;  HCQ: 37 (66.1%) vs 46 (83.6%);  IS including  i) MMF: 18 (32.1%) vs (30.9%); ii) Tac: 12 (21.4%) vs 22 (40%); iii) Jaki: 12 (21.4%) vs 7 (12.7%);  iv) others: 6 (10.7%) vs 9 (5.1%) | Pirfenidone (initial 300 mg/day, and increased to the maximum tolerable dosage or a maximum of 1,800 mg/day) for 24 weeks | ND | Pirfenidone group vs control group:  SSc: (FVC% change from baseline to 24 weeks) + 6.60% (3.10–8.46%) vs +0.55% (−6.80 -5.35%), p=0.042;  IIM: (FVC% change from baseline to 24 weeks) +7.50% (0.55–14.45%) vs + 1.00% (−4.65-7.43%), p=0.016;  RA (<70%-baseline DLCO% subset): (DLCO% change from baseline to 24 weeks) +7.40% (2.18–14.03%) vs – 6.60% (−8.60-2.13%), p=0.011  other CTDs: no significant difference between pirfenidone group and control group (numbers ND) | ND |
| **Observational studies** | | | | | | | |
| Fischer et al.., 2013  UK [75] | N=125 CTD-ILD including 44 SSc, 32 PM/DM, 19 lung-dominant CTD, 18 RA, 5 pSS, 4 SLE, and 3 MCTD  Age: 60.4±11.6  F: 42% | no | MMF (300mg/d in 65% of subjects): 125 (100%) including 62 (50%) as the first GC-sparing agent, 36 (29%) as replacement of CYC (because of CYC associated toxicity for the majority), and 15 (12%) as replacement of AZA (due to ILD progression for the majority)  Prednisone with moderate dose at MMF initiation (median 20 mg, IQR 10–35) | ND | Tapered prednisone and MMF (median duration 2.5 (IQR 1.3-4.2) years)  Median prednisone dose at 26 and  52 weeks after MMF initiation: 12mg/d and 5 mg/d, respectively (p < 0.0001 for each  compared to 20 mg at MMF initiation). | %FVC change from MMF initiation to 104 weeks: +6.1±1.8, p=0.0008;  %FVC change from MMF initiation to 156 weeks: +7.3±2.6, p=0.004;  %DLCO change from MMF initiation to 104 weeks: +7.1±2.8, p=0.001;  %DLCO change from MMF initiation to 156 weeks: +7.8±4.1, p=0.05;  %FVC significantly improved at 52, 104, and 156 weeks from MMF initiation for patients with non-UIP ILD and stablised for patients with UIP ILD.  Discontinuation of MMF: 13 (10%) due to gastrointestinal intolerance (3), ILD progression (2), hepatic ransaminase elevation (2), recurrent infections (1), cytopenias (1), and nonspecific symptoms (4) | ND |
| Oldham et al., 2016  USA [76] | N=97 fibrotic CTD-ILD including 22 PM/DM, 19 MCTD, 23 RA, 16 SSc, 13 pSS, and 4 SLE.  Age (CTD-ILD patients): 54.1±12.8 vs 52.7±11.1  F (CTD-ILD patients): 77.8% vs 67.4% | AZA vs MMF ---CTD-ILD: 54 vs 43  ---SLE-ILD: 3 vs 1 | AZA (median 125mg/d) + GC (≥20mg/d: 22.2%, <20mg/d: 59.3%)  vs  MMF (median 2000mg/d) + GC (≥20mg/d: 4.7%, <20mg/d: 65.1%) | ND | ND | Mortality (AZA vs MMF): 3 (5.6%) vs 4 (9.3%);  Transplant rate (AZA vs MMF): 2 (3.7%) vs 1 (2.3%);  Respiratory hospitalisation (AZA vs MMF): 17 (31.5%) vs 11 (25.6%);  Total adverse outcomes* (AZA vs MMF): 22 (40.7%) vs 16 (37.2%)  Adverse outcome* incidence rate ratio (IRR) for CTD-ILD patients (AZA vs MMF): 1.23 (0.49-3.12), p=0.66;  Adverse outcome IRR for UIP patients (AZA vs MMF): 0.83 (0.21-3.31), p=0.79  Other adverse events in AZA group: 6 (11.1%) including elevated transaminases (4), pancreatitis (1) and recurrent infection (1)  Yearly change of %FVC:  AZA 1.46% (0.12%-2.79%), p=0.033;  MMF -0.52% (-1.51%-0.47%), p=0.3;  Yearly change of %DLCO:  AZA 4.50% (1.25%-7.75%), p=0.007;  MMF -2.0% (-4.41%-0.41%), p=0.1  Treatment discontinuation due to clinical failure: 10% in both groups | ND |
| Wang et al., 2023  China [77] | N=245 CTD-ILD including 64 RA, 58 pSS, 49 PM/DM, 31 lung dominant CTD, 23 SLE, 12 SSc, and 8 MCTD.  Age (CTD-ILD patients): 57.0±4.8 vs 57.5±5.6  F (CTD-ILD patients): 42% vs 41% | MMF vs CYC ---CTD-ILD patients: 105 vs 140  ---SLE-ILD patients: 9 vs 14 | MMF (500mg BD) +PNL (40-80mg/d) vs CYC (oral 50mg once every other day) +PNL (40-80mg/d) | ND | Tapered PNL+ MMF (for 2 years) or CYC (cumulative dose should not exceed 10g) | Comparison within cohort (before vs after treatment):  MMF cohort:  i) %FVC improved from 64.9±7.2 at baseline to 71.0±5.2 after complete treatment, p<0.001;  ii) %DLCO improved from 62.3±8.7 at baseline to 71.4±8.4 after complete treatment.  CYC cohort:  i) %FVC improved from 66 (57.5-71) at baseline to 74 (70-79.5) after complete treatment, p<0.001;  ii) %DLCO improved from 63.2±8.6 at baseline to 73.0±7.5 after complete treatment.  Comparison between MMF and CYC cohorts after treatment:  i) change in %FVC: 8.57(11.57–4.55) vs 15.07(20–9.79), p<0.001;  ii) change in %DLCO: 15(17.32–11.43) vs 15.94(18.58–12.33), p>0.05;  iii) AE: leukopenia: 8% vs 39%, p<0.0001; thrombocytopenia: 2% vs 8%, p=0.046; serious AE related to treatment: 6% vs 14%, p=0.036;  iv) mortality due to any cause: 1% vs 6%, p=0.047 | ND |
| Feng, et al. 2024  China [78] | N=120 CTD-ILD including 40 SSc, 26 RA, 22 pSS, 24 PM/DM, and 8 SLE.  Age (CTD-ILD patients): 49.8±10.3 vs 50.6±10.3  F (CTD-ILD patients): 68.3% vs 61.7% | Oral CYC vs IV CYC ---CTD-ILD patients: 60 vs 60  ---SLE-ILD patients: 5 vs 3 | Oral CYC group: CYC (1-2mg/kg/d for one year, every 2 weeks of treatment followed by one week of discontinuation) +PNL (20-60mg/d)  IV CYC group: IV CYC (400-600mg fortnightly×3 months, and then prolonged to monthly for one year) +PNL (20-60mg/d) | ND | PNL tapered to <10mg/d in both groups  Cumulative CYC dose in oral and IV groups: 8.75 (7.30-11.50) g vs 8.20 (6.70-9.00) g, p<0.001 | Oral CYC group vs IV CYC group  i) Warrick score in HRCT:  Baseline: 20.3±5.1 vs 20.2±4.9 , p=0.927  6 months: 18.5 (9-28) vs 16 (7-27), p=0.037  ii) FVC:  Baseline: 67.6±9.8 vs 68.6±9.3, p=0.564  6 months: 68.9±9.4 vs 72.8±8.7, p=0.02  iii) DLCO:  Baseline: 57.1±9.7 vs 58.0±9.4, p=0.607  6 months: 59.2±8.5 vs 62.5±8, p=0.035  iv) 6MWD:  Baseline: 397.7±32.6 vs 389.5±31.4, p=0.162  6 months: 411.8±37.8 vs 427.8±37.7, p=0.021  v) AE: total AE 36.7% vs 18.3%, p=0.040; GI reaction 16.7% vs 10.0%, p=0.200; URTI: 5% vs 3%, p>0.999; Herpes zoster infection: 6.7% vs 3%, p=0.679; Leukocytopenia: 8.3% vs 1.7%, p=0.619 | ND |
| Liu, et al. 2024  China [79] | N=13 SLE-ILD  Age: 42.2 ± 17.0  F: ND | no | GC: 13 (100%);  CYC (IV monthly): 12 (92.3%) MMF: 1 (7.7%)  Regimen:  GC+CYC: 12 (92.3%);  GC+MMF+Pirfenidone: 1 (7.7%) | Pirfenidone (600 mg/d): 1 (7.7%) | ND | HRCT after 6 months of treatment: improvement in 8/13 (61.5%), including the patient receiving pirfenidone, no change in 3/13 (23.1%), and progression in 2/13 (15.4%) patients;  PFT after 6 months of treatment: improvement in 5/10 (50%) patients | ND |

* AEs were defined as death, lung transplantation and respiratory hospitalization.

**Abbreviation:** AE: adverse event; AZA: azathioprine; CTD: connective tissue disease; CYC: cyclophosphamide; DLCO: diffusing capacity of the lung for carbon monoxide; F: female; FVC: forced vital capacity; HR: hazard ratio; IIM: idiopathic inflammatory myositis; ILD: interstitial lung disease; IQR: interquartile range; IV: intravenous; IS: immunosuppressant; MCTD: mixed connective tissue disease; methyl-PNL: methylprednisolone; MMF: mycophenolate; ND: not described; PM/DM: polymyositis/dermatomyositis; PNL: prednisolone; pSS: primary SjÖgren syndrome; RA: rheumatoid arthritis; SLE: systemic lupus erythematosus; SSc: systemic sclerosis; UIP: usual interstitial pneumonitis.

**Supplementary Table 10. Shrinking lung syndrome**

| **Study** | **Population** | **Intervention** | | | **Response** | **Recurrence within follow-up duration** |
| --- | --- | --- | --- | --- | --- | --- |
|  |  | **Immunosuppressive therapy** | **Other therapy** | **Maintenance** |  |  |
| **Observational studies** | | | | | | |
| Deeb et al., 2018  Canada [83] | N=20  Age: 35.7±14.6  F: 90.9% | GC (average prednisone dose 28.7±14.7 mg/day): 19 (95%);  IS: 18/20 (90%) including AZA (100-150mg/day): 10 (50%);  MTX (15-20mg/week): 5 (25%);  MMF (2-3g/day): 3 (15%)  Antimalarials: 19 (95%)  Regimens: GC+ antimalarials (2, 10%); GC+ antimalarials+ IS (16, 80%); GC+ IS (1, 5%); antimalarials+ IS (1, 5%) | Inhaled β2-agonists: 4/20 (20%) | ND | Clinical improvement after an average of 2.8 months: 19/20 (95%);  Spirometrical improvement (mostly partial improvement): 14/16 (87.5%);  Mortality: 4/20 (20%) including one died of respiratory failure at 1 year and the other three died of other causes at 7-11 years. | Recurrence happened in 2/20 (10%) patients at 2 and 9 years, respectively; and responded to high dose GC and IV CYC |
| **Case series** | | | | | | |
| Karim et al., 2002  UK [84] | N=7  Age: range 14-47  F: 100% | GC: 7 (100%);  IS: 7 (100%) including AZA (4, 57.1%), CYC (2, 28.6%), MTX (1, 14.3%)  Regimens: GC dose increased to 40mg/d: 3/7 (42.9%);  New IS added on original GC dose (1 MTX and 1 IV CYC followed by MTX): 2/7 (28.6%);  Continuing original medications and dose (GC, HCQ, AZA): 2/7 (28.6%) | Theophylline: 1/7 (14.3%) | ND | Clinical improvement: 4/7 (57.1%);  Clinical stability: 2/7 (28.6%);  PFT improvement: 3/7 (42.9%);  PFT stability: 2/7 (28.6%)  Clinical and PFT deteriorated: 1/7 (14.3%) (case with IV CYC followed by MTX, without increasing PNL dose) | No recurrence reported during follow-up in the five patients with stable condition or improvement |
| Oud et al., 2005  Netherland [85] | N=5  Age: range 21-47  F: 100% | methyl-PNL pulse (1000mg/d for 3 days for 3 courses) followed by oral PNL (20mg/d): 2 (40%);  Oral PNL (40mg/d): 3 (60%);  IS (AZA, 175mg/d): 1 (20%)  Regimens: methyl-PNL followed by oral PNL and IS (1, 20%); methyl-PNL followed by oral PNL (1, 20%); oral PNL alone (3, 60%) | ND | PNL tapered to 7.5-10mg/d | Clinical improvement: 5/5 (100%)  PFT improved: 5/5 (100%), vital capacity (VC) improved from 27%-51% at baseline to 52%-87% at 6 weeks-4 months | No recurrence happened in 8-12 months of follow-up |
| Borrell et al., 2016  Spain [86] | N=9  Age: range 30-64  F: 100% | GC (increased dose): 9 (100%);  IS: 5 (55.6%) including MMF (4, 44.4%), MTX (1, 11.1%);  RTX: 5 (55.6%);  IVIG: 1 (11.1%)  Regimens: GC alone (3, 33.3%); GC+IS (1, 11.1%); GC+RTX (1, 11.1%); GC+IS+RTX (3, 33.3%); GC+IS+RTX+ IVIG (1, 11.1%) | β -agonists: 6/9 (66.7%);  Theophylline: 1/9 (11.1%) | ND | Within the follow-up duration (range 6-96 months)  Cured without functional sequelae: 1/9 (11.1%)  PFT restrictive defect improvement (defined as an increase in FVC ≥10% or DLCO≥15%): 2/9 (22.2%)  Stability (defined as changes in FVC <10% or DLCO<15%): 6/9 (66.7%) with 1 developing ILD 4 years later. | No recurrence reported in 6-96 months of follow-up |
| Duron et al., 2016  France [87] | N=15  Age: 27 (range 17-67)  F: 100% | GC: 11 (73%) including 6 with 1mg/kg/d and 5 with<0.5mg/kg/d);  IS: 7 (46.7%) including MMF 2 (13.3%), CYC followed by MMF 2 (13.3%), MTX 2 (13.3%), AZA 1 (6.7%);  RTX: 2 (13.3%) including one used after low-dose GC failure and one used as combination with GC and MMF;  Colchicine: 1 (6.7%)  Regimens: GC alone (4, 26.7%); IS alone (1, 6.7%); GC+ IS (5, 33.3%); GC+ RTX (1, 6.7%); GC+IS+RTX (1, 6.7%); colchicine alone (1, 6.7%). | β -mimetics: 2/15 (13.3%);  Physiotherapy: 3/15 (20%); | ND | Clinical outcome: improvement: 9/12 (75%); stability: 3/12 (25%)  PFT outcome: improvement: 7/10 (70%); stability: 2/10 (20%); mild impairment: 1/10 (10%)  Imaging outcome: improvement: 3/5 (60%); stability: 2/5 (40%)  Lost to follow-up: 3/15 (20%) | ND |

**Abbreviation:** AZA: azathioprine; CYC: cyclophosphamide; F: female; FC: functional class; HCQ: hydroxychloroquine; ILD: interstitial lung disease; IV: intravenous; IS: immunosuppressant; IVIG: intravenous immunoglobulin; methyl-PNL: methylprednisolone; MMF: mycophenolate; MTX: methotrexate; ND: not described; NSAIDs: non-steroids anti-inflammatory drugs; PFT: pulmonary function test; PNL: prednisolone; RTX: rituximab.

**Supplementary Table 11. RCTs of new SLE treatment that reported treatment response of** **cardiopulmonary manifestations**

| **Study** | **Design** | **Intervention** | **N** | **Patients with cardiopulmonary manifestations** | **Results** | **Comment** |
| --- | --- | --- | --- | --- | --- | --- |
| Wallace *et al* 2013 (ALLEVIATE 1 + 2 + Open label)  Multinational [93] | RCT in moderate/severe flaring SLE patient | Epratuzumab  360 mg/m^2^,  or 720 mg/m^2^,  or placebo.  Open label = 360 mg/m^2^ for all | 53 in Epratuzumab arms vs 37 in placebo arm | 30 (56.6%) vs 24 (64.9%) patients had cardiopulmonary manifestations in Epratuzumab arms vs placebo arm. | Better BILAG response for epratuzumab vs placebo at  week 48 (p = 0.028).  In the open-label phase, 29 patients had cardiopulmonary manifestations, 2 had BILAG A/B and both improved to BILAG C/D grade; however, 8/27 (29.6 %) patients worsened from BILAG C/D/E to grade A/B. | Efficacy on cardiopulmonary manifestations was uncertain |

Abbreviations: BILAG: British Isles Lupus Assessment Group; RCT; randomized clinical trial.
